# Estimating the accumulation and re-accumulation of commercial tobacco, electronic cigarette, and cannabis waste based on a stratified random sample of census blocks

**DOI:** 10.1371/journal.pone.0313241

**Published:** 2025-01-06

**Authors:** Georg E. Matt, Lydia Greiner, Kristina Tran, Joseph Gibbons, Michael Vingiello, Paula Stigler Granados, Ronald Shadbegian, Thomas E. Novotny

**Affiliations:** 1 Department of Psychology, San Diego State University, San Diego, California, United States of America; 2 Center for Tobacco and the Environment, San Diego State University, San Diego, California, United States of America; 3 Department of Sociology, San Diego State University, San Diego, California, United States of America; 4 School of Public Health, San Diego State University, San Diego, California, United States of America; 5 Department of Economics, San Diego State University, San Diego, California, United States of America; University of Alcala Faculty of Medicine and Health Sciences: Universidad de Alcala Facultad de Medicina y Ciencias de la Salud, SPAIN

## Abstract

We investigated the amount and distribution of waste generated by commercial tobacco, electronic cigarette, and cannabis (TEC) use to inform policy options aimed at mitigating the environmental harm caused by these products. Using disproportionate stratified random sampling, we selected 60 census blocks from the eight largest cities in San Diego County, California. We twice surveyed publicly accessible areas in these blocks to quantify TEC waste accumulation and its re-accumulation. All collected items were photographed, classified, geocoded, counted, and properly discarded. We identified demographic, land use, and behavioral data from public sources and direct observations. We modeled total cigarette butt quantities for all census blocks across the eight cities and found similar results for Round 1 (8.63 million) and Round 2 (8.66 million) collections. Single-use plastic cigarette filters were the primary contributor to TEC waste (94%). Total TEC waste counts and cigarette butt counts showed strong linear associations (r = +0.86 and r = +0.91). The area surveyed, land use category, resident demographics, smoking prevalence, and walkability explained 78% of the variance in cigarette butt count. The interval between Round 1 and 2 counts did not affect re-accumulation counts, suggesting that baseline TEC waste levels were re-established within 1–2 months after cleanup. Annually, we estimate up to 200 million cellulose acetate plastic filters may be discarded in public areas of the eight cities. Given the continuous deposition, vast quantity, heterogeneous distribution, and rapid re-accumulation of TEC waste after cleanup, increasing removal efforts alone are financially untenable and impractical downstream solutions for TEC waste. Community-wide policies (e.g., filter bans, outdoor smoking restrictions) and individual behavior changes (e.g., reduced smoking rates, proper disposal of cigarette butts) are necessary to effectively mitigate the environmental impact of TEC waste in urban settings.

## Introduction

The World Health Organization estimates that globally, in 2022, 34.4% of men and 7.4% of women aged 15 years and older used some form of commercial tobacco [1]. This translates into 1.245 billion commercial tobacco users, of which 890 million smoked cigarettes and consumed approximately 4.8 trillion cigarettes. In the United States, the overall consumption in 2020 is estimated at 216 billion cigarettes [2]. While California’s adult cigarette smoking prevalence is relatively low compared to the overall prevalence in the United States (6.2% vs. 12.5% in 2021), cigarette consumption in 2020 was approximately 14 packs per capita, which equates to 11 billion cigarettes or approximately 12 cigarettes per day per smoker in California [3]. While the WHO does not provide comparable global figures for electronic tobacco products, the observed global trends are alarming. The most recent data for the United States indicates that in 2023, 6.6% of adults (17.2 million), 10% of high school students (1.56 million), and 4.6% of middle school students (0.55 million) were current users of e-cigarettes [4, 5].

More than 90% of cigarettes smoked worldwide have filters made of cellulose acetate, a type of plastic. That number is even higher in the United States, where 99% of cigarettes smoked have such filters [6]. It is estimated that from 25% to more than two-thirds of cigarettes smoked are discarded into the environment as part of habitual behavior [7]. Bio- and photodegradation of filters is limited by environmental conditions. Over time and through exposure to the elements, the plastic filters break up into smaller pieces and, eventually, microplastics. The harmful chemicals accompanying them may then leach into the environment [8, 9], and include tobacco-specific nitrosamines, heavy metals, and nicotine [7, 10, 11]. Toxicological studies indicate the potential for bioaccumulation and biomagnification of some toxins in marine and freshwater organisms, which could affect human and wildlife health through the food chain [12–14].

With the increased popularity of vaping, heated tobacco, smokeless tobacco, and other non-combusted tobacco products, as well as the legalization of recreational cannabis in some states, the environmental impact of discarded products is becoming an important consideration. According to the Centers for Disease Control and Prevention (CDC) data for the United States from January 2020 to May 2022, e-cigarette unit sales increased 67.2% from 15.5 million to 25.9 million units per month [15]. Thereafter, from May to December 2022, total sales decreased by 12.3% to 22.7 million units per month, and from January 2020–December 2022, the percentage of total e-cigarette sales that were prefilled cartridges decreased from 75.2% to 48.0%. In contrast, the percentage of total sales that were disposable e-cigarette sales more than doubled, from 24.7% in January 2020 to 51.8% in December 2022. Of note is the nicotine content of vaping products. As of March 2022, 80.9% had >5% nicotine content, and 97% had ≥2% nicotine content [16].

With the increasing use of disposable e-cigarettes, there is potential for added environmental harm if these products are discarded similarly to cigarette butts. All electronic devices share major components and design characteristics that present potential chemical hazards (e.g., nicotine, heavy metals, tobacco-specific nitrosamines) and electronic waste hazards (e.g., circuit boards, batteries). Components include a cartridge or pod holding the nicotine vaping solution, a heating element, a battery, a microprocessor, and a metal or plastic enclosure that houses these components. Of these components, the nicotine-containing cartridge, battery, and microprocessor are regulated under municipal, state, and federal hazardous and electronic waste regulations. In 2015, the U.S. Environmental Protection Agency (EPA) ruled that because e-cigarettes include cartridges that contain nicotine, “e-cigarettes therefore may be regulated as acute hazardous waste P075 when disposed” [17]. The disposal of nicotine-containing cartridges or pods falls under the federal EPA rules for "unused, discarded nicotine products." That is, if a cartridge or pod has not been completely used and still contains nicotine, it is considered an acute hazardous waste under federal regulations, and disposal should occur at a commercial facility or retail location. If a pod or cartridge has been consumed, and nicotine has been depleted, it is no longer a hazardous waste under federal regulations, although stricter state-level standards may apply [18, 19]. Increasing trends in the use of these products suggest that the billions sold each year will contribute tens of thousands of tons of non-biodegradable waste into the environment [20]. A 2023 study involving 23 countries conducted by the United Nations Institute for Training and Research reported that 844 million vaping devices are discarded each year, amounting to 42,000 metric tons of e-waste, which is not generally recycled [21].

Cigarette filters are the single most littered item worldwide, representing 20–40% (by count) of all debris items found on beaches and urban settings [7]. The 314,000 volunteers for the 2022 International Coastal Cleanup (ICC) event collected 1.9 million cigarette butts. By comparison, the ICC reports that 1.2 million beverage bottles, 1 million food wrappers, and 0.8 million bottle caps were collected. Cigarette butts outranked other littered items in North America, Latin America, Europe, and Africa. In California, 39,170 volunteers for the 2023 Coastal Cleanup Day picked up 138,277 cigarette butts, followed by 45,539 food wrappers, 23,643 bottle caps, 13,540 plastic bottles, and 11,965 straws/stirrers [22].

Studies have found tobacco contaminants present in various environmental settings, including water, soil, dust, and plants. There is evidence that drinking water could be a significant exposure route to toxic chemicals due to discarded TEC waste. Other contaminants and plastic particles may leach into the environment from cigarettes and e-cigarette components (e.g., metals, PAHs, TSNAs, and plastic nanoparticles). These leachates have been shown in laboratory studies to have toxic effects on a wide variety of cellular, vertebrate, and invertebrate organisms. Some may bioaccumulate in plants and animals and pose additional exposure risks to humans consuming them; there is no definitive evidence for the public health implications of this potential. Some recent studies using mice and human cell-based assays suggest that tobacco waste pollution is toxic to humans, though the potential pathways of exposure to tested pollutants is not known. Accidental ingestion of cigarette butts is hazardous to humans due to nicotine poisoning, especially among children [23]. As described above, unused nicotine-containing products are listed as hazardous wastes under the US Environmental Protection Agency’s regulatory system [24].

**Fig 1:**
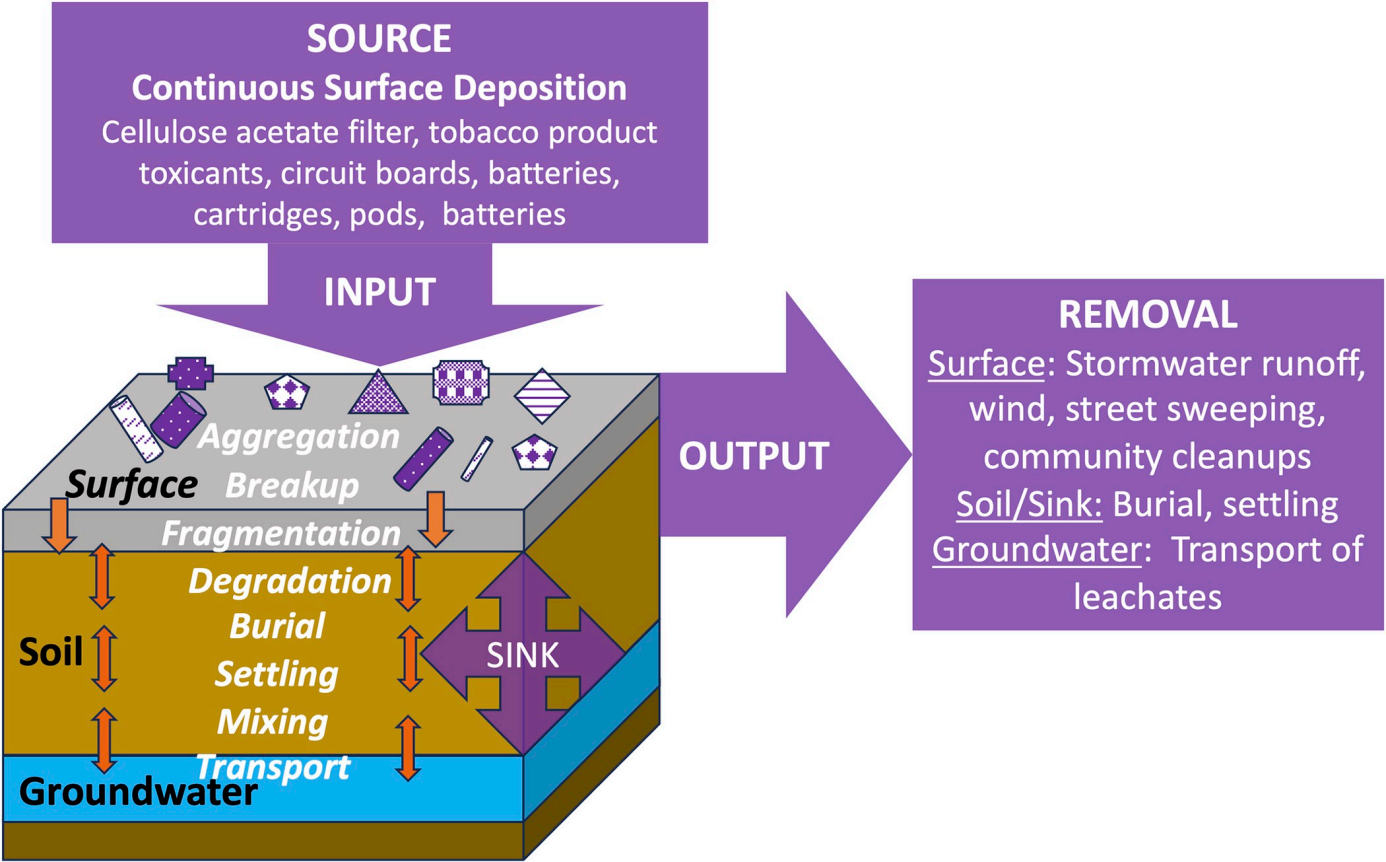
Input-output box model of TEC waste surface accumulation.

We developed an input-output box model to show how commercial tobacco, electronic cigarettes, and cannabis (TEC) waste accumulates over time (Fig 1). This model shows how TEC waste items are discarded by users (i.e., input) at a particular rate (i.e., source rate) and are removed at a particular rate (removal rate) through multiple processes (i.e., output). This input-output system is in a steady state when the source rate equals the removal rate, resulting in a constant amount of TEC waste on the surface. When the source rate is higher than the removal rate, TEC waste accumulates in the environment. If the removal rate is larger than the source rate, TEC waste in the environment declines. Removal processes include runoff in municipal stormwater systems, trash cans, butt receptacles, street sweeping, and community clean-ups. In addition, discarded TEC waste undergoes various transformations, including breakup, fragmentation, degradation, burial, settling, and mixing, leading to deposition in the various sub-surface environmental compartments where transformed products may remain for extended periods before decomposing or being removed.

While community cleanups draw attention to the magnitude and ubiquity of the TEC waste, they collect only a fraction of the total discarded TEC products and thus do not represent total environmental TEC waste. Community cleanups rely on volunteers without specific training in identifying and classifying TEC waste, lack standardized collection protocols, and do not represent all areas where TEC waste is present. Moreover, all cleanups are limited to visible evidence of TEC waste and cannot collect disintegrated waste products.

To address some of the existing gaps in research on TEC waste, we designed this study to: (a) develop a reproducible protocol for identifying and classifying TEC waste, (b) estimate the amount and types of TEC waste in a Southern California urban setting, and (c) model the occurrence of cigarette butt waste at the census block level as a function of land use and resident characteristics. Because we repeated the TEC waste survey in each selected census block, we also evaluated (d) the impact of a thorough TEC waste cleanup on the re-accumulation of such waste in a follow-up survey. This repeated measurement provides insights into the impact and sustainability of downstream mitigation efforts.

## Methods

### Census block sampling

We selected the eight largest cities in San Diego County for this study: Carlsbad, Chula Vista, El Cajon, Escondido, Oceanside, San Diego, San Marcos, and Vista. These cities are part of the Centers for Disease Control’s (CDC) PLACES data set that includes detailed local information about smoking prevalence and other health behaviors. Based on their 2010 boundaries, these cities comprise 16,914 census blocks with 2.39 million residents in 2022. Census blocks are the smallest geographic area for which the U.S. Bureau of the Census collects and tabulates decennial census data [25]. Census blocks were stratified by six land use and two community-level socioeconomic categories. Based on the San Diego Area Governments (SANDAG) land-use coding system [26], we created the following six census block-level land use categories: 1- Low-Density Residential Alone; 2- Mixed High/Low Density Residential; 3- Mixed Entertainment Residential; 4- Mixed Park Residential; 5- Mixed Residential Miscellaneous; 6- Mixed Nonresidential Miscellaneous. We used a principal component analysis of socioeconomic characteristics collected in the 2015–2019 American Community Survey (ACS) to create an index of socioeconomic status (see the online supplemental material S1 Table for additional information) [27]. Areas with index scores above the mean were identified as having “high,” while those at or below the mean were identified as having “low” socioeconomic status. Following a disproportionate stratified sampling design, five census blocks were randomly selected from each of the 12 strata for a total sample size of 60 census blocks. Two census blocks were found inaccessible for data collection and were replaced with randomly selected blocks from the same strata. We determined population sampling weights to estimate mean and total counts in linear models.

### Community engagement

We approached this project as an opportunity to engage communities in identifying and addressing the neighborhood problem of tobacco product waste. To do so, we developed and implemented a strategy to involve a broad range of stakeholders in the project. In each of the eight cities in which data collection was planned, we sent an introductory e-mail and invited city council members, city managers, and relevant city offices (e.g., Public Works, Parks and Recreation) to meet and learn more about the project. In addition, we invited community groups, tobacco control programs, environmental organizations, business districts, and other stakeholders. Regular one-on-one meetings with stakeholders and annual updates maintained interest in the project. Throughout the study, volunteer participation in data collection was actively encouraged. All volunteers received an online orientation to the project and on-site training in advance of TEC waste collection that was led by trained research staff. At the conclusion of data collection, we prepared a summary and hosted a series of community discussions of preliminary findings and possible solutions.

### TEC waste survey protocol

We developed the collection protocol based on a review of previous tobacco product waste studies in urban and coastal areas [28–31] to establish reproducible methods for locating, identifying, and quantifying TEC waste across different urban environments. The complete protocol is available in the online supplemental material (S1 File) and addresses the following steps: 1) Training of staff and volunteers; 2) Employing tools and equipment for locating, identifying, and recording TEC waste; 3) Assessing safety and census block characteristics; 4) Determining boundaries and measuring areas to be surveyed; 5) Developing strategies to survey different types of surface areas; 6) Establishing guidelines for locating TEC waste in identified areas; 7) Developing guidelines for identifying and recording different types of TEC waste and their locations; 8) Establishing guidelines for quantifying the amount of TEC waste; and 9) Establishing guidelines for reporting TEC waste.

### TEC waste definitions

We assigned each item identified as TEC waste to one of the following six categories:

#### Cigarette butts

This category refers to the remains of a smoked or partially smoked commercial tobacco cigarette, whether filtered or non-filtered, regardless of the state of degradation or fragmentation.

#### Other tobacco products

This category includes any discarded cigar components, including tips; smokeless tobacco waste products, such as snus pouches; the packaging from any tobacco product, including tins and plastic wrapping; and commercial tobacco advertising elements and coupons.

#### E-cigarettes

This category includes any part of vaping or other electronic delivery systems, regardless of the product vaporized in the device (tobacco, cannabis, nicotine, THC, other material), as well as packaging, warning labels, and advertising materials.

#### Cannabis products

This category includes any commercial cannabis product, including cannabis-containing cigarettes, edibles, and their packaging and advertising.

#### Dual use

This category includes any products used to smoke tobacco, cannabis, or other materials, including lighters, rolling papers, and hand-rolled butts.

#### Unsure

This category includes suspected TEC waste that could not be assigned to one of the other categories.

### Data collection

Each census block was surveyed twice. The Round 1 survey evaluated surface accumulated TEC waste and included the removal of all TEC waste items. This survey involved: a) pre-assessment to determine feasibility and boundaries; b) active assessment of block characteristics and surface area measurement; and c) collection, geocoding, identification, recording, and disposal of TEC waste items. The Round 2 survey assessed the re-accumulation of TEC waste since the Round 1 survey, repeating steps b) and c).

#### Pre-assessment

Each census block was first inspected using Google Maps to determine its boundaries (i.e., each street on the perimeter of the block), any interior areas that could be surveyed, and if TEC waste could be safely collected in publicly accessible areas. Census block boundaries were recorded using ArcGIS [32, 33]. Each census block could contain two types of survey areas–regular or irregular. Regular areas were defined as the sidewalk, curb strip, and gutter of streets on the perimeter or inside of the census block, as well as any alleys inside the census block. Irregular areas were defined as any additional publicly accessible, walkable areas inside the census block that could be safely surveyed, including parking lots, shopping areas, picnic areas, and hiking trails.

#### Active assessment

Prior to the TEC waste collection, each regular and irregular area was visually inspected to determine certain block characteristics, henceforth referred to as ‘Points of Interest.’ These included land use (e.g., housing, entertainment, parks, retail); city services (e.g., bus stops, storm drains, trashcans); and tobacco-related behaviors (e.g., observed smoking, smoking signage, butt receptacles). Research staff walked the perimeter and inside survey areas to photograph, classify, and record Points of Interest using ArcGIS and to take coordinates of the inside and outside boundaries of each surveyed area for surface area calculations.

#### TEC waste survey

All surfaces within the boundaries designated in the active assessment as regular and irregular areas were surveyed for TEC waste. Loose debris, including leaves or trash, was raked and inspected for any hidden items. Research assistants and volunteers worked in small teams of 4–6 using either the "snowplow" or the "lawnmower" methods (See the online supplemental material S1 File) [34]. For "regular areas", every TEC waste item was photographed and geocoded where it was found; the type and number of item(s) on each photograph was entered into ArcGIS using the QuickCapture app; each item was picked up and sorted into containers by type; and each container was weighed. For "irregular areas", each TEC waste item was picked up, sorted by type, and counted. The total count of each type for an "irregular area" was entered into ArcGIS.

### Measures

#### Characteristics of census blocks and their residents

Data for census block area (m^2^), population count, population density, age, sex, race, and ethnicity were obtained from the 2010 decennial census [35]. Data for census tract educational attainment, income, rent, home value, poverty level, cash-based public assistance, (e.g., *Temporary Assistance for Needy Families*, the *Supplemental Nutrition Assistance Program*, and *Supplemental Security Income*), unemployment, and foreign-born status were obtained from the 2019 *American Community Survey* [27]. *The National Walkability Index*, developed by the US EPA, ranks groups of census blocks according to their relative walkability [36]. The raw score and a normalized 0 to 1 score were used in our analysis.

Smoking prevalence data were obtained from the *Behavioral Risk Factor Surveillance System* [37]. This index estimates the percentage of residents aged ≥18 years who report having smoked ≥100 cigarettes in their lifetime and currently smoke every day or some days [37].

Land use category was based on the SANDAG land-use coding system [26] as described above.

#### TEC waste-related characteristics

As summarized above and described in detail in the online supplement (S1 File), we surveyed TEC waste in two types of public spaces within each census block: *regular* and *irregular* areas. The regular area included (1) the space from the inside edge of the sidewalk to the gutter, including the curb strip (landscaped area between the edge of the sidewalk and curb) along the perimeter of the census block and any streets within the perimeter, and (2) any alleys within the perimeter, which were measured from "edge to edge." The irregular area included any other public space inside the perimeter, such as parking lots, shopping plazas, or other businesses. Area sizes (m^2^) were determined using ArcGIS and combined to create the total surface area surveyed for each block.

Municipal street sweeping schedules, posted on city websites or street signs, were used to calculate the number of days from the last street sweeping to the TEC waste survey on each street in each census block. Street sweeping schedules varied widely, from never to three times each week. TEC waste surveys were scheduled to maximize the length of time between sweeping and survey. The number of days since the last rain event was calculated for each TEC waste survey. Rainfall data were obtained from the weather station closest to the census block centroid through an interactive map at wundermap.com. The dates of the TEC surveys were used to calculate the number of days between the first and second rounds of data collection. The first round of data collection started in July 2021 and ended in October 2022; the second round started in May 2022 and ended in February 2023. The second round was conducted in reverse order (i.e., round two assessments began with the blocks most recently completed in round one). The number of trashcans and the number of butt receptacles per block were determined based on observations during the collection events.

We photographed and categorized each TEC waste item in the field as one of the six defined land use categories. We counted each type of waste collected and summed these counts to create a total count per census block.

#### TEC waste count validation

To determine the proportion of TEC waste items potentially missed during the two Rounds of data collection, we re-surveyed a sample of both Round 1 and Round 2 data collection areas. In Round 1, we reassessed 12 of the 60 census blocks and observed a 12% undercount of collected TEC waste. In Round 2, we reassessed 26 of the 60 census blocks and observed an 8% undercount. Based on these findings, we adjusted our observed counts of TEC waste by 10% to correct for this predictable undercounting.

### Data analysis

We estimated the population-weighted *mean counts per block* for each type of TEC waste and for the sum of all types. We also estimated the TEC waste *total counts* expected for the population of census blocks of the eight cities. Because cigarette butts comprised >94% of TEC waste, we limited our multivariable modeling to only cigarette butt counts. We considered count models (Poisson and negative binomial) and OLS linear regression models with log-transformed counts. Because of overdispersion, we ruled out Poisson models, and we found negative binomial and log-linear regression models to provide equivalent results. For ease of interpretation, we report findings from the log-linear models. We used a mixed-linear random effects model to examine changes in TEC waste counts between Rounds 1 and 2. For each model, we started by first including the survey surface area for each block as an explanatory variable. We then added variables describing residents and land use of the census block or tract in which the block was located. We evaluated interaction effects to examine if associations between cigarette butt count and resident characteristics are conditional on land use. Applying a Type I error rate of 5%, none of the interaction effects were statistically significant. To protect against the undue influence of unusual census blocks and overfitting, we applied robust standard errors and conducted k-fold cross-validation of the final model and estimates. All analyses were conducted using Stata V.18 [38].

## Results

### Census block characteristics

The census blocks varied considerably in area (1,417–399,448 m^2^), population size (4–881), and sociodemographic characteristics (e.g., Median age: 23–73 years; foreign-born: 5–54% % White: 1–76%; poverty level: 3–15%), median income, rent, and property values, and walkability (Table 1). Similarly, the areas within each census block that were surveyed for TEC waste ranged from 212 m^2^ to 57,040 m^2^, with a median of 2,994 m^2^. The time intervals between Round 1 and 2 surveys ranged from 36 days to 592 days, with a median of 266 days. The median number of days since the last street sweeping were 7 and 9 days in Rounds 1 and 2, respectively. The last rain event occurred a median of 8 and 10 days before the Round 1 and 2 surveys, respectively.

**Table 1:** Census block (n = 60), resident, and TEC waste survey characteristics.

	Mean	SD	Min	Q1	Median	Q3	Max
**Characteristics of Census Blocks and their Residents**							
**Block Area (m** ^**2**^ **)** ^**a**^	39,783	61,112	1,417	9,947	16,911	40,652	399,448
**Mean Population** ^**a**^	129.33	181.99	4	40.5	112.5	226.25	881
**Population Density (persons/km** ^**2**^ **)** ^**a**^	4,408	6,964	551	2,885	6,618	7,452	45,408
**Median Age (years)** ^**a**^	29.72	19.48	22.8	28.73	35.9	44.7	72.5
**Female (%)** ^**a**^	48.03	10.63	0	45.37	49.25	53.53	66.67
**Race (%)** ^**a**^							
** White (non-Hispanic)**	50.97	27.52	1.39	26.15	56.58	75.67	100
** Black(non-Hispanic)**	4.98	10.6	0	0	1.39	4.81	0.33
** Asian**	9.13	12.32	0	0	4.83	11.8	57.26
** Hispanic**	32.13	26.52	0	10.98	21.88	55.4	93.97
**Foreign Born (%)** ^**b**^	22.38	12.57	4.79	10.94	19.57	30.93	51.75
**Educational Attainment (% college)** ^**b**^	42.98	20.44	0.68	21.50	39.6	58.4	90.09
**Median Income ($)** ^**b**^	79,615	27,697	32,087	63,369	79,692	90,659	186,116
**Median Rent ($)** ^**b**^	1,818	425.2	1,062	1,530	1,717	2,135	3,351
**Median Home Value ($)** ^**b**^	584,239	303,998	73,200	407,000	473,800	679,900	1,711,000
**Poverty (%)** ^**b**^	10.94	6.85	2.52	5.94	8.69	15.47	35.11
**Public Assistance (%)** ^**b**^	2.50	2.89	0	0.78	1.58	2.8	18.9
**Unemployment (%)** ^**b**^	3.52	2.5	0.54	2.07	3.04	4.36	13.87
**Smoking Prevalence (%)** ^**c**^	11	3.11	7	8.68	10	12.3	20
**Trash Cans (Number)**	1.45	3.27	0	0	0	1	16
**Butt Receptables (Number)**	0.38	1.61	0	0	0	0	10
**Raw Walkability Index** ^**d**^	14.25	2.96	7.17	13.08	14.83	16.42	19.17
**Normalized Walkability Index (scale of 0–1)** ^**d**^	0.71	0.15	0.36	0.65	0.74	0.82	0.96
**Characteristics of TEC Waste Surveys**							
**Area Surveyed (m** ^**2**^ **)** ^**e**^							
** Regular Area**	2,930	3,223	212	1,057	1,995	3,437	18,026
** Irregular Area**	4,180	9,735	0	0	105	2,843	50,682
** Total**	7,110	11,524	212	1,687	2,994	5,637	57,040
**Days Between Collections Round 1 and Round 2** ^**e**^	285.57	166.71	36	132.5	265.5	432	591
**Days Since Last Street Sweeping** ^**f**^							
** Round 1**	16.38	17.92	0	2	6.5	31.5	55
** Round 2**	15.74	16.36	0	2.38	9.25	27	58
**Days Since Last Rain** ^**g**^							
** Round 1**	19.16	37.68	1	3	8	18	258
** Round 2**	29.89	34.51	1	3	9.5	48.5	111

Min: minimum; Q1: first quartile; Mdn: Median; Q3: third quartile; Mean: arithmetic mean; GMean: geometric mean; CI confidence interval

^a^ Block-level data from the 2010 U.S. Census [35].

^b^ Tract-level data from the 2019 American Community Survey.

^c^ tract-level data from the Behavioral Risk Factor Surveillance System [37].

^d^ EPA National Walkability Index [36].

^e^ GIS data collected for N = 60 census blocks.

^f^ Street sweeping data obtained from local city governments.

^g^ Rainfall data obtained from the closest weather station.

### Accumulated TEC waste count estimates

#### Mean count estimates per census block

The overall mean TEC waste count was 494 items per block (Table 2). By far the highest arithmetic mean count of any TEC waste type was cigarette butts (Mean = 464), followed by other tobacco product waste (Mean = 19), dual-use waste (Mean = 4), e-cigarette waste (Mean = 3), and cannabis waste items (Mean = 3).

**Table 2:** Accumulated TEC waste (Round 1) counts per block and density per 100m^2^.

Type of TEC Waste	Min	Q1	Mdn	Q3	Max	Mean	95% CI		GMean	95% CI	
**Count per Block**											
** Cigarette Butts**	1	89	266	602	2886	464.1	[121.7;	809.1]	150.9	[86.1;	264.4]
** Other Tobacco Product**	0	3	6	19	200	19.5	[2.7;	30.9]	6.3	[2.7;	10.7]
** Cannabis Product**	0	0	1	3	28	2.6	[0.8;	5.3]	1.0	[0.4;	1.9]
** E-cigarette**	0	0	1	3	26	3.0	[0.2;	6.9]	0.3	[0.1;	1.1]
** Dual-Use**	0	1	3	10	88	4.1	[1.8;	7.4]	1.1	[0.3;	2.2]
** Unsure**	0	0	0	1	9	0.4	[0;	3.4]	0.2	[0.1;	0.4]
** All TEC Waste**	4	98	286	622	3135	493.7	[130.0;	859.2]	171.2	[102.1;	287.0]
**Density (Count per 100m** ^**2**^ **)**											
** Cigarette Butts**	0.1	3.5	6.9	14.5	43.2	10.9	8.2;	13.7	7.1	[5.5;	9.3]
** Other Tobacco Product**	0	0.1	0.2	0.5	4.7	0.4	1.2;	1.6	0.3	[0.2	0.4]
** Cannabis Product**	0	0	<0.1	0.1	1.0	0.1	<0.1;	0.1	<0.1	[<0.1;	0.1]
** E-cigarette**	0	0	<0.1	0.1	1.9	0.1	<0.1;	0.1	<0.1	[<0.1;	0.1]
** Dual-Use**	0	<0.1	0.1	0.2	1.3	0.2	0.1	0.3	0.2	[0.1	0.2]
** Unsure**	0	0	0	<0.1	0.3	<0.1	<0.1;	<0.1	<0.1	[<0.1;	<0.1]
** All TEC Waste**	0.3	3.8	7.4	15.6	45.5	11.7	8.8	14.6	7.7	[5.9	10.0]

Min: minimum; Q1: first quartile; Mdn: Median; Q3: third quartile; Mean: arithmetic mean; GMean: geometric mean; CI confidence interval

#### Population-based estimated counts of accumulated TEC waste

Based on the probability weights from the disproportional sampling design, we estimated the total number of TEC waste items that could be collected based on the populations inhabiting the 16,912 blocks in the eight cities of this study. We project 7.85 million cigarette butts, 330,000 other tobacco-related waste items, 69,000 dual-use items, 50,000 e-cigarette items, and 45,000 cannabis waste items to accumulate as a steady state total on the publicly accessible surfaces surveyed in this study (Table 3). The total count for all TEC waste items is projected at 8.35 million items, of which cigarette butts accounted for 94%. Given the estimated 10% survey undercount for the covered surface area, the adjusted total count for cigarette butts is estimated at 8.63 million, and for all TEC waste items, the adjusted total is estimated at 9.18 million.

**Table 3:** Population-based estimated counts of accumulated TEC waste (Round 1) by type.

Type of TEC Waste	Estimated Count	95% CI	
**Cigarette Butts**	7,848,690	[3,553,121;	12,144,259]
**Other Tobacco Product Waste**	330,317	[67,817;	592,818]
**Cannabis Product Waste**	44,537	[4,605;	84,468]
**E-cigarette Waste**	50,313	[12,403;	88,223]
**Dual-Use Waste**	69,375	[39,111;	99,639]
**Unsure**	5,925	[2,076;	9,773]
**All TEC Waste**	8,349,157	[3,730,059;	12,968,255]

Estimated counts are not adjusted for 10% undercount.

### Factors affecting cigarette butt accumulation

We developed weighted OLS regression models with robust variance estimates to examine the associations between cigarette butt counts per block and the characteristics of the blocks. The included variables were surface area surveyed, land use type, walking index, number of men and women living in the block, number of trash cans and butt receptacles, SES, smoking prevalence, foreign-born, ethnicity/race, municipal street sweeping, and rain events. The overall model shows an excellent fit with R^2^ = 0.78 (F(12,47) = 8.69, p<0.0001) (Table 4). SES, race/ethnicity, number of foreign-born residents, trash cans, and butt receptacles were not associated with cigarette butt counts after controlling for the area surveyed and smoking rate. Similarly, the number of rain events and the amount of rainfall were not associated with cigarette butt counts. While the number of days since the last street sweeping was not associated with cigarette butt counts, whether or not streets were subject to municipal street sweeping was associated. We also tested for interaction effects with SES, land use, and smoking prevalence, but none were statistically significant.

**Table 4:** Population-weighted OLS regression model of Round 1 cigarette butt counts per block.

Explanatory Variables	$\hat{\beta }$	p-value	ΔR^2^
**Area Surveyed (100 m** ^**2**^ **)** ^**a**^	0.0026	<0.001	0.1861
**Male Residents (N)**	0.0105	0.013	0.0315
**Female Residents (N)**	-0.0097	0.018	0.0291
**Smoking Prevalence (%)**	0.0564	0.025	0.0417
**Land Use (Ref: Residential low-density)**		0.005	0.1388
** Residential low/high-density**	-0.0743	0.749	
** Residential and entertainment**	0.5729	0.028	
** Residential and parks**	0.4956	0.075	
** Residential and miscellaneous**	0.4131	0.180	
** Nonresidential and miscellaneous**	0.6480	0.011	
**EPA Walkability Index**	0.0696	0.018	0.0614
**Street Sweeping**		0.018	0.0940
** Not Subject to Sweeping**	-0.7524	0.005	
** Days Since Last Sweeping** ^**a**^	-0.0008	0.909	
**Constant**	0.2974	0.610	

^a^ Counts were log10 transformed. Days since last street sweeping was mean-centered. Model estimates with hc2 robust standard errors. p-values refer to the H_0_: *β* = 0 of the corresponding parameter. ΔR^2^ reflects the unique proportion of variance accounted for by a particular variable; i.e., it is the change in R^2^ if a variable is omitted from the model.

The area surveyed accounted for 18.6% of the total variance, followed by land use (13.9%), street sweeping (9.4%), walkability index (6.1%), smoking prevalence (4.2%), number of male residents (3.2%), and number of female residents (2.9%). Larger surface areas surveyed; more male residents; higher smoking prevalence; residential land use mixed with entertainment, parks, commercial, and nonresidential uses; and higher walkability index were all associated with higher cigarette butt counts. In contrast, more female residents, residential-only land use, and blocks not subject to street sweeping all showed lower cigarette butt counts.

### Re-accumulated TEC waste counts

#### Mean counts per census block

We found that the mean re-accumulated TEC waste was 495 items per block. Cigarette butts were by far the most common type of TEC waste (Mean = 465), followed by other tobacco product waste (Mean = 17), dual-use waste (Mean = 5), e-cigarette waste (Mean = 4), and cannabis waste (Mean = 3). The Round 2 mean counts for different types of TEC waste (Table 5) were within five items of their Round 1 means (Table 2).

**Table 5:** Re-accumulated TEC waste (Round 2) counts per block and density per 100m^2^.

Type of TEC Waste	Min	Q1	Mdn	Q3	Max	Mean	95% CI		GMean	95% CI	
**Count per Census Block**											
**Cigarette Butts**	2	60	231	555	3729	465.4	[121.7;	809.1]	104.9	[121.7;	809.1]
**Other Tobacco Product Waste**	0	3	8	14	167	16.8	[2.7;	30.9]	4.3	[2.0;	7.8]
**Cannabis Product Waste**	0	0	1	3	23	3.0	[0.8;	5.3]	0.3	[0.1;	1.7]
**E-cigarette Waste**	0	0	1	3	38	3.6	[0.2;	6.9]	0.2	[0.1;	1.3]
**Dual-Use Waste**	0	1	3	7	76	4.6	[1.8;	7.4]	0.9	[0.1;	2.2]
**Unsure**	0	0	0	0	27	1.3	[0;	3.4]	0.1	[0;	0.74]
**All TEC Waste**	2	69	255	601	3984	494.6	[130.0;	859.2]	117.1	[130.0;	859.2]
**Density (Count per 100m** ^**2**^ **)**											
**Cigarette Butts**	0.2	2.5	5.8	11.7	44.3	8.5	[6.3;	10.7]	5.6	[4.3;	7.3]
**Other Tobacco Product Waste**	0	0.1	0.2	0.4	3.1	0.4	[0.3;	0.5]	0.3	[0.2;	0.4]
**Cannabis Product Waste**	0	0	<0.1	0.1	1.0	0.1	[<0.1;	0.1]	0.1	[<0.1;	0.1]
**E-cigarette Waste**	0	0	<0.1	0.1	1.9	0.1	[<0.1;	0.2]	0.1	[<0.1;	0.1]
**Dual-Use Waste**	0	<0.1	0.1	0.1	1.3	0.2	[0.1;	0.2]	0.1	[0.1;	0.2]
**Unsure**	0	0	0	<0.1	0.3	<0.1	[0;	<0.1]	<0.1	[0;	<0.1]
**All TEC Waste**	0.2	2.6	6.6	12.9	46.6	9.2	[6.9;	12.6]	6.1	[4.7;	9.0]

Min: minimum; Q1: first quartile; Mdn: Median; Q3: third quartile; Mean: arithmetic mean; GMean: geometric mean; CI confidence interval

#### Population-based re-accumulated TEC waste counts

Based on the probability weights from the disproportional sampling design, we project the total number of re-accumulated TEC waste items that could be collected in the publicly accessible areas of all 16,912 blocks of the eight cities to be 8.37 million. Of this projected total, 7.87 million (94.1%) are cigarette butts, 284,000 (3.4%) are other tobacco-related items, 78,000 (0.9%) are dual-use items, 61,000 (0.7%) are e-cigarette items, and 51,000 (0.6%) are cannabis items (Table 6). Given the estimated 10% undercount for the surveyed area, the adjusted population-based total count for all TEC waste items is 9.20 million and for cigarette butts 8.66 million.

**Table 6:** Population-based estimated total counts of re-accumulated TEC waste (Round 2) by type.

Type of TEC Waste	Estimated Count	95% CI	
**Cigarette Butts**	7,870,929	[2,057,482;	13,684,375]
**Other Tobacco Product Waste**	283,655	[45,073;	522,237]
**Cannabis Product Waste**	50,971	[12,834;	89,107]
**E-cigarette Waste**	60,584	[3,908;	117,260]
**Dual-Use Waste**	78,169	[30,642;	125,696]
**Unsure**	21,202	[1;	57,465]
**All TEC Waste**	8,365,509	[2,199,110;	14,531,908]

Estimated counts are not adjusted for a 10% undercount.

### Correlation of Round 1 and Round 2 cigarette butt counts

**Fig 2:**
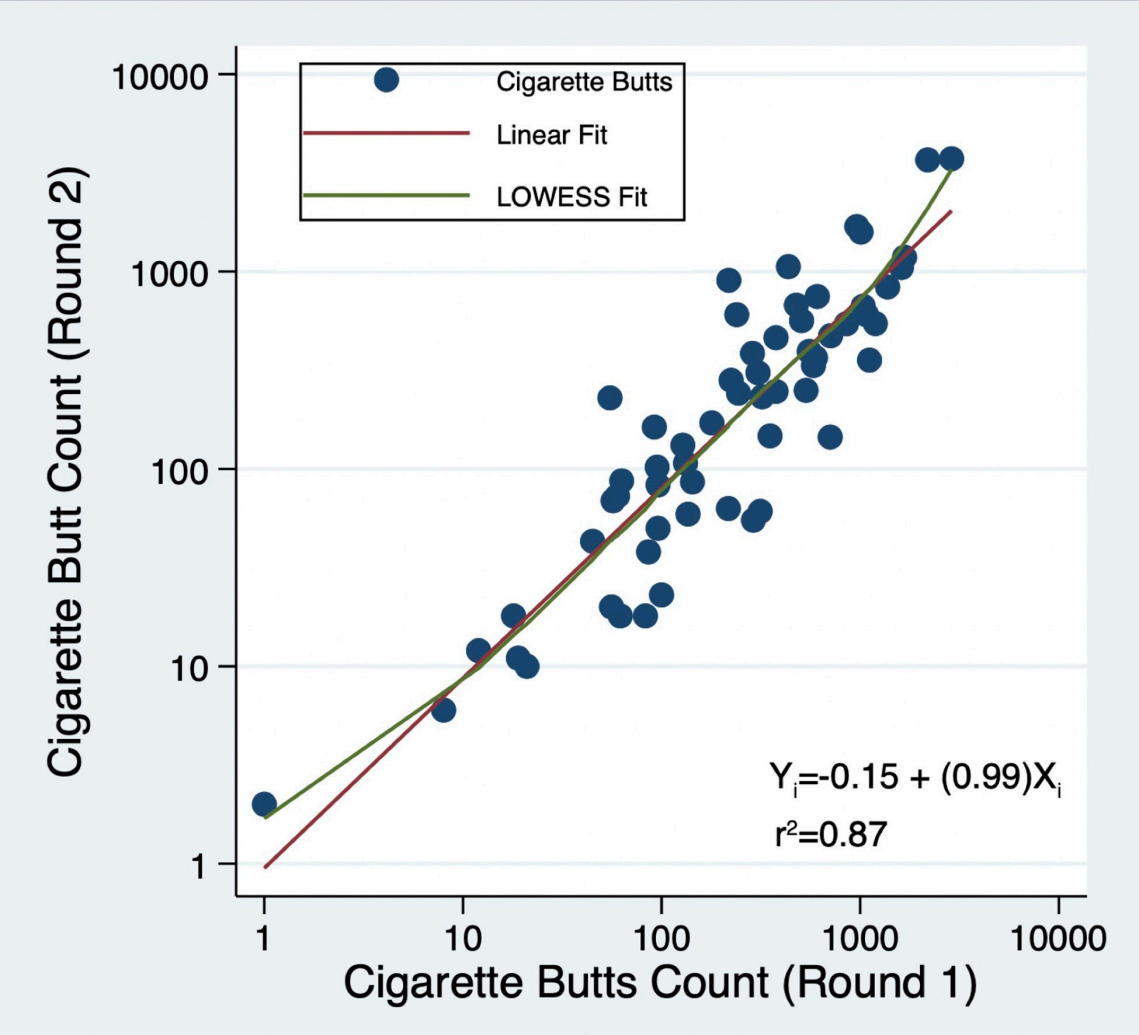
Correlation of cigarette butts counted and removed in Round 1 with cigarette butts counted in Round 2.

The Round 1 and 2 counts were strongly correlated (r^2^ = 0.87; F(1,58) = 132.6, p<0.0001) (Fig 2). The linear fit slope was 0.99 (95% CI: [0.82; 1.17]), and the intercept was -0.15 (95%CI = [-0.54, 0.25], indicating cigarette butt counts in Round 1 and Round 2 were at equivalent levels across the range of block counts. Such a strong association is particularly noteworthy considering that the Round 2 surveys were conducted between 36 and 591 days after the Round 1 survey.

### Factors affecting cigarette butt re-accumulation

Round 2 counts are likely to reflect newly discarded cigarette butts (i.e., re-accumulation since Round 1). Similar to the modeling of cigarette butt count in Round 1, we examined the determinants of re-accumulated cigarette butts in census blocks, including the characteristics of the blocks, the demographics and smoking prevalence of people living in the blocks, the presence of trash cans and butt receptacles, the history of municipal street sweeping, and the occurrence of rain events. In addition, linear and quadratic polynomial terms were added for days between the Round 1 and 2 surveys to examine the rate at which cigarette butts re-accumulated. Again, SES, race/ethnicity, smoking rates, trash cans, and butt receptacles were not associated with cigarette butt counts after controlling for the number of male, female, and foreign-born residents. Similarly, rain events were not associated with cigarette butt counts, but municipal street sweeping was associated. We also tested for interaction effects with SES, land use, and smoking prevalence, but none were statistically significant.

**Fig 3:**
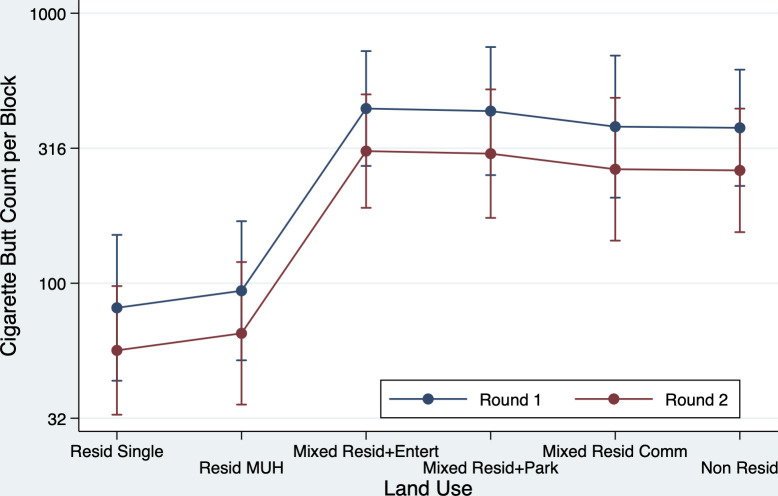
Geometric mean cigarette butt counts for different land use categories in Rounds 1 and 2.

The full model describing days since the Round 1 cleanup showed excellent fit with R^2^ = 0.79 (F(13,46) = 21.33, p<0.0001), but no association was found for cigarette butt re-accumulation and the number of days since TEC waste was collected in Round 1 (F(2,46) = 0.003, p = 0.970) (Table 7). The overall fit of the reduced model (without days since Round 1) shows that the explanatory variables account for 78% of the variance of re-accumulated cigarette butts (F(11,48) = 24.58, p<0.0001). The surveyed surface area uniquely accounted for 21.1% of the total variance, followed by land use (12.3%), walking index (6.7%), street sweeping (4.5%), and number of male (3.0) and female residents (2.4%). Larger areas surveyed; more male residents; residential land use mixed with entertainment, parks, commercial, and nonresidential uses; and higher walking index were all associated with higher cigarette butt counts. In contrast, more female residents, residential-only land use, and blocks not subject to street sweeping all showed lower cigarette butt counts. Fig 3 shows the predicted counts by land-used category.

**Table 7:** Population-weighted OLS regression model of Round 2 cigarette butt counts per block.

	Full Model: R^2^ = 0.7860 (F(13,46) = 21.33, p<0.0001			Reduced Model: R^2^ = 0.7829 (F(11,48) = 24.58, p<0.0001		
Explanatory Variables	$\hat{\beta }$	p-value	ΔR^2^	$\hat{\beta }$	p-value	ΔR^2^
**Area surveyed (100m** ^**2**^ **)**	0.0030	<0.001	0.2116	0.0029	<0.001	0.2114
**Male residents (N)**	0.0118	0.002	0.0320	0.0111	0.001	0.0304
**Female residents (N)**	-0.0098	0.011	0.0246	-0.0093	0.005	0.0237
**Land Use (Ref: low-density residential)**		0.005	0.1239		0.006	0.1226
** Residential low/high-density**	-0.0077	0.937		-0.0047	0.985	
** Residential and entertainment**	0.6536	0.004		0.6387	0.003	
** Residential and parks**	0.5349	0.033		0.5185	0.038	
** Residential and miscellaneous**	0.4278	0.142		0.4052	0.147	
** Nonresidential and miscellaneous**	0.7396	0.001		0.7301	0.001	
**EPA Walkability Index**	0.0880	0.019	0.0864	0.0883	0.014	0.0666
**Last Street Sweeping**		0.005	0.0463		0.006	0.0450
** Not Subject to Sweeping**	-0.5776	0.006		-0.5606	0.008	
** Days Since Last Sweeping**	-0.0063	0.496		-0.0061	0.461	
**Days Since Cleanup**		0.970	0.0031			
** Linear**	0.0003	0.639				
** Quadratic**	-0.0000	0.921				
**Constant**	0.4787	0.399		0.4791	0.406	

Counts were log10 transformed. The area surveyed, days since the last street sweeping, and days since round 1 cleanup were mean-centered. Model estimates with hc2 robust standard errors. P-values refer to the H0: *β* = 0 of the corresponding parameter. ΔR^2^ reflects the unique proportion of variance accounted for by a particular variable; i.e., the change in R^2^ if a variable is omitted from the model. The full model includes linear and quadratic terms for days since cleanup, which the reduced model omitted.

### Mean change in cigarette butt counts

To determine the overall change in mean cigarette butt counts between Round 1 and Round 2 surveys, we examined a mixed linear model in which census block was a random factor, Rounds 1 and 2 were the repeated data collections, and the characteristics of blocks and their residents as well as the time interval between Rounds 1 and 2 were the explanatory variables (Table 8). The interval between Rounds 1 and 2 was not associated with cigarette butt counts, nor was the interaction of land use and time interval. The number of male and female residents, smoking prevalence, street sweeping, and days since cleanup were not significant predictors of butt counts. The area surveyed, the number of foreign-born residents, mixed residential and nonresidential land use, and walking index were associated with higher butt counts. In contrast, residential areas and Round 2 counts showed significantly lower levels of butt count changes. The model showed an excellent fit with *χ*^2^(9) = 124.63, p<0.0001. The Round 2 coefficient of -0.1577 (log10 scale) indicates an overall decline in cigarette butt counts of approximately 30% independent of the area surveyed, foreign-born residents, land use, and walkability (Fig 2).

**Table 8:** Population-weighted mixed linear effects model of cigarette butt counts in rounds 1 and 2.

Explanatory Variables	$\hat{\beta }$	p-value
**Area surveyed (100m** ^**2**^ **)**	0.0027	<0.001
**Foreign Born Residents (N)**	0.0144	<0.001
**Land Use (Ref: Residential low-density)**		<0.001
** Residential low/high-density**	0.0822	0.678
** Residential and entertainment**	0.7461	<0.001
** Residential and parks**	0.7077	<0.001
** Residential and miscellaneous**	0.6253	0.001
** Nonresidential and miscellaneous**	0.6624	<0.001
**EPA Walkability Index**	0.0690	0.001
**Round 2 (Ref: Round 1)**	-0.1577	0.001
**Constant**	0.5657	0.103

Counts were log10 transformed. The area surveyed was mean-centered. Model estimates with robust standard errors. P-values refer to the H_0_: *β* = 0 of the corresponding parameter.

## Discussion

We found that cigarette butts were the single most commonly collected type of TEC waste, accounting for 94% of all items collected in 60 census blocks in San Diego, California. This is consistent with previous studies conducted in multiple settings [39, 40]. Our Round 1 TEC waste collection in 60 San Diego County census blocks revealed an average of 464 butts collected per census block (Median = 266), which translates into a mean of 10.9 cigarette butts collected per 100 m^2^ of surveyed area (Median = 6.9 per 100 m^2^). E-cigarette and cannabis waste were collected at rates of <0.1 items per 100 m^2^. Thus, the overwhelming proportion of TEC waste that is improperly discarded in the sampled census blocks is from filtered commercial cigarette smoking. Webler & Jakubowski have argued that improperly discarding cigarette butts after smoking is part of the smoking ritual [41]. The continuation of this ritualistic behavior may stem from the habitual nature of the behavior as well as from a lack of information about cigarette butt toxicity and its potential impacts on the environment. An increase in indoor smoking bans may also contribute to an increase in TEC waste in outdoor environments [7]. In addition, unless cannabis products involve a filter attachment of other non-organic components, they may more readily decompose and be more difficult to locate.

We observed large differences in the collection of cigarette butts across census blocks, of which 78% could be accounted for by characteristics of the census blocks and their residents. Census blocks with mixed residential and nonresidential use showed counts 4.6 to 5.6 times higher than those with low-density residential land use and 3.8 to 4.6 times higher than high-density residential blocks. Independent of land use, higher numbers of male residents, higher smoking prevalence, and higher walkability were associated with increased cigarette butt collection counts. In contrast, higher numbers of female residents and streets not subject to street sweeping showed lower collection counts. All of these variables reflect human activities (e.g., entertainment, parks, walkability) and characteristics of residents known to be associated with commercial tobacco use (e.g., smoking prevalence, gender). Controlling for land use, smoking prevalence, and gender, conventional sociodemographic characteristics (e.g., age, race, ethnicity, income, education) did not account for differences in cigarette butt collection. This suggests that while there are large differences in commercial tobacco use among sociodemographic groups, the more proximal factors (smoking prevalence, human activity, land use) contributing to smoking behavior and the habitual discarding of cigarette butts are shared across different sociodemographic environments. The finding that the presence of trash cans and butt receptacles were not associated with TEC waste counts may reflect a bidirectional causal relationship. That is, the accumulation of discarded TEC waste may cause collection devices to be placed in a particular location, the use of which may in turn lower the number of discarded TEC waste items. Based on our findings, there is no noticeable impact of such devices on discarded TEC waste. The lack of an association also suggests that the habitual and perhaps ritualist behavior of discarding cigarette butts may be difficult to modify with the placement of waste receptacles.

Our data collection protocol called for removing all TEC waste during the first survey so that the counts during the second survey might reflect newly discarded items. The total number of TEC waste items overall, and cigarette butts in particular, were virtually identical in Rounds 1 and 2, with the population total counts projected at 9.18 million and 9.20 million for all census blocks in the eight cities. The TEC waste estimates we report are likely still undercounts of the total numbers discarded in the environment of these eight cities.

Undercounting may be due to several different issues. First, our survey was based on fragments large enough to be detected by the human eye and recognizable as TEC waste. Over time, these items break up into fragments too small to identify by sight, change in appearance, and become buried or mixed with soil and debris; therefore, many were not included in our count. Second, our human efforts to detect all TEC waste items are known to be imperfect, and our own validation exercise suggested that we missed approximately 10% of identifiable TEC waste items in the areas surveyed. Third, our survey was limited to publicly accessible areas of a census block. With a median census block area of 16,911 m^2^ (Mean 39,783 m^2^) and a median survey area of 2,994 m^2.^(Mean = 7,110 m^2^), our count estimates represent approximately 18% of the total census block area. Fourth, what can be counted and collected at any point in time is the net difference between deposition and removal and not the sum of all discarded TEC waste. The unknown number of TEC items removed by municipal and private efforts is not included in this count. Finally, there is also a possibility of slight overcounting, which may have occurred if TEC waste items disintegrated into multiple parts and each part was counted as a separate item.

The strong linear association (r^2^ = 0.87) between Round 1 and 2 counts suggests a high degree of stability in TEC waste surface accumulation at the census block level. Surprisingly, we did not find any associations (linear or quadratic) between the time interval and the repeat collection counts. Because these repeated counts were stable over intervals ranging from 36 to 591 days between cleanups, our data suggest that existing source deposition and removal processes across the 60 census blocks may have re-established an equilibrium of about 30,000 cigarette butts within a few weeks to months of the initial cleanup.

While the total count was very similar for the two TEC waste collections, it is important to note that the centers of the two distributions describing the typical census block showed a significant reduction of about 30% in the geometric means and 11% in median counts. This was possible because the distributions of TEC waste have a strong positive skew with medians and geometric means lower than the arithmetic means. The observed reduction in the center of the distributions (i.e., geometric mean and medians) while the arithmetic mean remained stable has two important implications. First, the reduction in TEC waste for typical blocks was compensated by increases in blocks with relatively high counts, thus canceling each other out in the overall total count estimation. Second, the intensive removal of TEC waste in Round 1 had a persistent impact on the center of the count distribution, as indicated by lower collection geometric mean and median counts in Round 2.

Similar to Round 1, we observed large differences between blocks in cigarette butt re-accumulation counts, of which characteristics of census blocks and their residents accounted for 78%. With the exception of smoking prevalence, the same predictors accounted for differences in Round 1 and 2 collections. In Round 2, the cigarette butt counts were 4 to 5 times greater in nonresidential blocks than in low- or high-density residential blocks. Independent of land use category, blocks with higher numbers of male residents and higher walkability showed significantly greater butt re-accumulation. In contrast, blocks with higher numbers of female residents and blocks excluded from municipal street sweeping had lower re-accumulation counts. All of these factors were independent of the time interval between the two surveys, and we did not observe any interactions between land use and Round 1 vs 2 surveys. This suggests that the re-accumulation of cigarette butt counts did not depend on land use and that TEC waste at Round 2 returned to Round 1 baseline levels within a relatively short period of time for all land use categories.

As an additional verification of our results, it is informative to compare the direct count estimates offered in this study to estimates based on California’s adult smoking rate (6.2% in 2021) [3], the eight cities’ adult population (1.892 million in 2022) [42], and the average daily adult cigarette consumption of 6–12 cigarettes per day [43–45]. Based on the eight cities’ adult smoker populations (117,293) and a discard rate of 25% [7], we estimate that 64–128 million cigarettes are improperly discarded annually in our study area. For a discard rate of 67% [7], we estimate that 171–342 million cigarettes were improperly discarded annually in the eight communities. These figures are within the range of the population-based projections based on direct TEC waste counts in our 60 census blocks of 55–110 million cigarettes discarded annually, assuming re-accumulation of 9.2 million cigarette butts every 1–2 months. Given that our surveys examined only 18% of the total census block surface areas, the total burden of discarded cigarette butts in the eight cities may well exceed 200 million per year.

It is essential to consider what environmental consequences might be possible, given 100–200 million discarded cigarette butts per year in the eight communities included in this study. Cigarette butts consist mainly of plastic filters that can release numerous toxic chemicals into the environment, including carcinogens and reproductive toxicants such as nicotine, tobacco-specific nitrosamines (TSNAs), polycyclic aromatic hydrocarbons (PAHs), and heavy metals [23, 46]. There are numerous laboratory-based studies reporting the adverse effects of cigarette butt leachates on fresh and saltwater organisms, including single-celled organisms, invertebrates such as mussels, and vertebrates such as fish [46]. Plants show alterations in growth patterns due to exposure to cigarette butt leachates. In addition, human and animal poison control centers report adverse effects among children and pets that accidentally ingest butts [47]. Gong et al. examined the airborne emissions of styrene, 2-methyl-2 cyclopenten-1-one, naphthalene, triacetin, and nicotine from cigarette butts at different temperatures [48]. Their study showed the emitted nicotine mass from a butt over five days was similar to the mass emitted from mainstream and sidestream smoke of one cigarette. Roder-Green et al. examined the release of nicotine from butts in standing water and estimated that 7.3 mg of nicotine per gram of butt was eluted, the majority within the first 27 minutes [40]. They also reported that one discarded cigarette butt could contaminate 1,000 L of surface water with nicotine concentrations that are above established toxicity levels. Roadside cigarette butt litter has been shown to disburse nicotine, metals, and PAHs [49]. Moreover, leachates from discarded cigarette butts are one of several sources that may contribute nicotine pollution to sensitive ecosystems through stormwater discharges [49, 50].

There is also a growing concern that cellulose acetate plastic filter waste contributes to microfibers and microplastics in the aquatic environment. Belzagui et al. found that each smoked filter released approximately 100 microfibers per day in two weeks of observation and that an estimated 0.3 million tons of microfibers might be reaching aquatic environments per year [51]. Cellulose acetate (most likely from discarded filters) has been detected in urban stormwater runoff in the San Francisco Bay Area, suggesting that filters should be regulated as a source of plastic pollution [52]. There is increasing concern and evidence that microfibers, in general, have adverse effects on human health as they are inhaled, consumed, or otherwise introduced into the human body [53].

### Limitations

This study is based on a disproportionate stratified random sample of census blocks to represent the diversity of land use and sociodemographic characteristics of census blocks in San Diego County. Available financial resources limited the total sample size to 60 blocks, reducing statistical power and the precision of the estimates, especially those for population subgroups in census blocks. Because the unit of analysis of this study was the census block, our focus was necessarily on explanatory variables at the census block level. Future studies should investigate littering behavior, attitudes, and knowledge among individuals to better understand how to change behavior among TEC users. The focus of our TEC waste survey was on publicly accessible areas, excluding private properties, and was limited to street surfaces within approximately 50 cm of the curb. Thus, our projections of potential TEC waste underestimate the actual burden of discarded TEC waste in the census blocks sampled. The research design did not allow us to systematically vary or randomize the interval between the round 1 and 2 data collections. Round 2 data collection was scheduled in approximately reverse order to the Round 1 data collection, but weather and staffing considerations required making exceptions to this rule. Thus, there is the possibility of confounding due to irregular inter-collection time intervals. The rapid re-accumulation of TEC waste and the lack of association between collection and inter-collection time intervals suggests that future studies should examine repeated collections over short time intervals (i.e., days to week) to better understand the time course of re-accumulation. We could not evaluate the dynamic input-output processes, especially the types of removal processes, that allowed the re-accumulation of cigarette butts found in survey Round 2. The relatively small sample size of census blocks raises the concern of model overfitting. To protect against capitalizing on chance, overfitting, and the undue influence of unusual data points, we used robust standard errors and subjected final models to cross-validation. While the overall proportions of variance showed the expected shrinkage, the statistical significance of the model predictors remained. Field data collection for this study took place between July 2021 and February 2023, when COVID-19 restrictions were gradually lifted. It is likely that our count estimates are affected by COVID-19-related changes in human activity and smoker behavior. The combined impact of these changes is unknown as smoking prevalence during COVID appears to have increased [54] and reduced human activity in public areas (e.g., shopping malls, entertainment districts) may have lowered the amount of accumulated TEC waste.

## Conclusions

Our findings about the amount and type of surface accumulation and re-accumulation of TEC waste are important in considering policies to address TEC waste in urban environments. This is the first study to investigate the surface accumulation and re-accumulation of TEC waste across different urban land-use categories. While the results of this study are likely to underestimate the total burden of TEC waste, we believe that our methods may be used to predict the quantity of discarded cigarette butts in similar urban areas. Such findings may then be used to model the economic costs of the manual removal of this waste and to inform policymakers and the public about the persistent, ubiquitous, and preventable toxic wastes that result from cigarettes and other forms of tobacco and cannabis use [55]. Given the continuous deposition, vast quantity, heterogeneous distribution, and rapid re-accumulation of TEC waste after cleanups, intensive surface abatement efforts alone are impractical and prohibitively costly. This study may inform policy options regarding the current negotiations on an international treaty to reduce plastic pollution [20, 21]. Community-wide policies (e.g., filter bans, outdoor smoking restrictions) and individual behavior changes (e.g., reduced smoking rates, proper disposal of cigarette butts) may be effective in mitigating the environmental impact of TEC waste in urban settings.

## Supporting information

S1 Table Index of socioeconomic characteristics derived from the 2015–2019 American Community Survey (ACS). (PDF)

S1 File Data collection protocol for the assessment of waste discarded in urban environments from commercial tobacco, electronic cigarette, and cannabis use. (PDF)

S1 Fig Map of San Diego County showing the 60 census blocks randomly selected from the eight largest cities. (PDF)

S2 Fig Example census block showing TEC waste items recorded in regular and irregular areas surveyed by research staff. (PDF)

## References

[1] World Health Organization. WHO global report on trends in prevalence of tobacco use 2000–2030 Geneva: World Health Organization, 2024.

[2] Drope J, Hamill S, Chaloupka F, Guerrero C, Lee HM, Mirza M, et al. The Tobacco Atlas. New York: Vital Strategies and Tobacconomics, 2022.

[3] California Department of Public Health CTCP. California Tobacco Facts and Figures 2016. Sacramento, CA: California Department of Public Health, 2016.

[4] Norris T, Adjaye-Gbewonyo D, Bottoms-McClain L. Early Release of Selected Estimates Based on Data From the 2023 National Health Interview Survey. U.S. Department of Health and Human Services, Centers for Disease Control and Prevention, National Center for Health Statistics, 2023.

[5] Birdsey J., Cornelius M., Jamal A., Park-Lee E, Cooper MR, Wang J, et al. Tobacco Product Use Among U.S. Middle and High School Students—National Youth Tobacco Survey, 2023. http://dxdoiorg/1015585/mmwrmm7244a1. 2023;27:1173–82. 10.15585/mmwr.mm7244a1. 37917558 PMC10629751

[6] United States Federal Trade Commission. Federal trade commission cigarette report for 2020 and smokeless tobacco report for 2020. Washington, DC: United States Federal Trade Commission, 2021.

[7] Novotny TE, Slaughter E. Tobacco Product Waste: An Environmental Approach to Reduce Tobacco Consumption. Current environmental health reports. 2014;1:208–16. Epub 2014/08/26. doi: 10.1007/s40572-014-0016-x ; PubMed Central PMCID: PMC4129234. 25152862 PMC4129234

[8] Schneider JE, Peterson NA, Kiss N, Ebeid O, Doyle AS. Tobacco litter costs and public policy: a framework and methodology for considering the use of fees to offset abatement costs. Tobacco Control. 2011;20(Suppl 1):i36–i41. doi: 10.1136/tc.2010.041707 21504923 PMC3088473

[9] Hon NS. Photodegradation of cellulose acetate fibers. Journal of Polymer Science: Polymer Chemistry Edition. 1977;15(3):725–44. doi: 10.1002/pol.1977.170150319

[10] Booth DJ, Gribben P, Parkinson K. Impact of cigarette butt leachate on tidepool snails. Mar Pollut Bull. 2015;95(1):362–4. Epub 20150422. doi: 10.1016/j.marpolbul.2015.04.004 . 25913792

[11] Slaughter E, Gersberg RM, Watanabe K, Rudolph J, Stransky C, Novotny TE. Toxicity of cigarette butts, and their chemical components, to marine and freshwater fish. Tobacco control. 2011;20 Suppl 1:i25–9. Epub 2011/04/29. doi: 10.1136/tc.2010.040170 ; PubMed Central PMCID: PMC3088407. 21504921 PMC3088407

[12] Xu EG, Richardot WH, Li S, Buruaem L, Wei HH, Dodder NG, et al. Assessing Toxicity and in Vitro Bioactivity of Smoked Cigarette Leachate Using Cell-Based Assays and Chemical Analysis. Chem Res Toxicol. 2019;32(8):1670–9. Epub 2019/07/10. doi: 10.1021/acs.chemrestox.9b00201 . 31286770

[13] Richardot WH, Yabes L, Wei HH, Dodder NG, Watanabe K, Cibor A, et al. Leached Compounds from Smoked Cigarettes and Their Potential for Bioaccumulation in Rainbow Trout (Oncorhynchus mykiss). Chem Res Toxicol. 2023. Epub 20231012. doi: 10.1021/acs.chemrestox.3c00167 . 37827523 PMC10664143

[14] Alejo J. Cigarettes and Marine Systems: Exploring the Bioaccumulative Effects of Tobacco Waste on the Marine Bioindicator Species, Macoma nasuta. San Diego, California: San Diego State Univeristy; 2022.

[15] Ali FR, Seidenberg AB, Crane E, Seaman E, Tynan MA, Marynak K. E-cigarette Unit Sales by Product and Flavor Type, and Top-Selling Brands, United States, 2020–2022. MMWR Morb Mortal Wkly Rep. 2023;72:672–7. doi: 10.15585/mmwr.mm7225a1 37347717 PMC10328473

[16] Ali FRM, Seaman EL, Crane E, Schillo B, King BA. Trends in US E-cigarette Sales and Prices by Nicotine Strength, Overall and by Product and Flavor Type, 2017–2022. Nicotine & Tobacco Research. 2022;25(5):1052–6. doi: 10.1093/ntr/ntac284 36580384 PMC10077931

[17] U.S. Environmental Protection Agency. HAZARDOUS WASTE STATUS OF E-CIGARETTES UNDER RCRA. In: Agency USEP, editor. Washington, DC: OFFICE OF SOLID WASTE AND EMERGENCY RESPONSE; 2015.

[18] Hendlin YH. Alert: Public Health Implications of Electronic Cigarette Waste. Am J Public Health. 2018;108(11):1489–90. Epub 2018/10/12. doi: 10.2105/AJPH.2018.304699 ; PubMed Central PMCID: PMC6187764. 30303735 PMC6187764

[19] Hendlin YH. E-cigarettes and a new threat: How to dispose of them: Univeristy of California; 2018 [updated 10/3/18; cited 20120 1/10/20]. Available from: https://www.universityofcalifornia.edu/news/e-cigarettes-and-new-threat-how-dispose-them.

[20] Gutterman LR. Vape Waste—The environmental harms of disposable vapes.: U.S. PIRG Education Fund; 2023 [cited 2024 August 2024]. Available from: https://pirg.org/edfund/resources/vape-waste-the-environmental-harms-of-disposable-vapes/

[21] Baldé CP, Yamamoto T, Forti V. Statistical briefing on invisible e-waste for International E-waste Day. Bonn, Germany: United Nations Institute for Training and Research, 2023.

[22] Ocean Conservancy. #SeatheChange. International Coastal Cleanup. 2023Report. Washington DC: Ocean Conservancy, 2023.

[23] Beutel MW, Harmon TC, Novotny TE, Mock J, Gilmore ME, Hart SC, et al. A Review of Environmental Pollution from the Use and Disposal of Cigarettes and Electronic Cigarettes: Contaminants, Sources, and Impacts. Sustainability. 2021;13(23):12994. doi: 10.3390/su132312994

[24] Krause MJ, Townsend TG. Hazardous waste status of discarded electronic cigarettes. Waste Manag. 2015;39:57–62. Epub 20150304. doi: 10.1016/j.wasman.2015.02.005 . 25746178

[25] U.S. Bureau of the Census. Geographic Areas Reference Manual. In: Commerce USDo, editor. Washington, DC: U.S. Department of Commerce; 1994.

[26] San Diego Association of Governments (SANDAG). GIS land use Ccodes. San Diego, CA: San Diego Association of Governments, 2021.

[27] U.S. Census Bureau. American Community Survey (ACS) 2023 [1/21/23]. Available from: https://www.census.gov/programs-surveys/acs.

[28] Marah M, Novotny TE. Geographic patterns of cigarette butt waste in the urban environment. Tobacco control. 2011;20 Suppl 1:i42–4. Epub 2011/04/29. doi: 10.1136/tc.2010.042424 ; PubMed Central PMCID: PMC3088466. 21504924 PMC3088466

[29] Valiente R, Escobar F, Pearce J, Bilal U, Franco M, Sureda X. Estimating and mapping cigarette butt littering in urban environments: A GIS approach. Environ Res. 2020;183:109142. Epub 20200117. doi: 10.1016/j.envres.2020.109142 ; PubMed Central PMCID: PMC7167348. 32004828 PMC7167348

[30] Liu TK, Wang MW, Chen P. Influence of waste management policy on the characteristics of beach litter in Kaohsiung, Taiwan. Mar Pollut Bull. 2013;72(1):99–106. Epub 20130511. doi: 10.1016/j.marpolbul.2013.04.015 . 23673204

[31] Mock J, Hendlin YH. Notes from the Field: Environmental Contamination from E-cigarette, Cigarette, Cigar, and Cannabis Products at 12 High Schools—San Francisco Bay Area, 2018–2019. MMWR Morb Mortal Wkly Rep. 2019;68(40):897–9. Epub 20191011. doi: 10.15585/mmwr.mm6840a4 ; PubMed Central PMCID: PMC6788397. 31600185 PMC6788397

[32] Environmental Systems Research Institute. ArcGIS Pro Desktop. Release 3.0. Redlands, CA: Environmental Systems Research Institute; 2022. Available from: https://www.esri.com/en-us/arcgis/about-arcgis/overview.

[33] Environmental Systems Research Institute. ArcGIS 2019. Available from: https://www.esri.com/en-us/arcgis/about-arcgis/overview.

[34] Moore S, Hale T, Weisberg SB, Flores L, Kauhanen P. California Trash Monitoring Methods and Assessments Playbook. Richmond, CA: San Francisco Estuary Institute, 2020 Contract No.: SFEI Publication #1025.

[35] United States Census Bureau. Decennial Census by Decade, 2010 2010. Available from: https://www.census.gov/programs-surveys/decennial-census/decade.2010.html.

[36] U.S. Environmental Protection Agency. National Walkability Index. Methodology and User Guide. Washington, DC: U.S. Environmental Protection Agency, 2021.

[37] Centers for Disease Control and Prevention. PLACES: Local Data for Better Health. 2023 [cited 2023 10/16/23]. Available from: https://www.cdc.gov/places/index.html.

[38] StataCorp. Stata statistical software: Release 17. College Station, TX: Stata Corporation; 2021.

[39] Healton CG, Cummings KM, O’Connor RJ, Novotny TE. Butt really? The environmental impact of cigarettes. Tobacco control. 2011;20 Suppl 1:i1. Epub 2011/04/29. doi: 10.1136/tc.2011.043729 ; PubMed Central PMCID: PMC3088438. 21504916 PMC3088438

[40] Roder Green AL, Putschew A, Nehls T. Littered cigarette butts as a source of nicotine in urban waters. Journal of Hydrology. 2014;519:3466–74. doi: 10.1016/j.jhydrol.2014.05.046

[41] Webler T, Jakubowski K. Attitudes, Beliefs, and Behaviors about Cigarette-Butt Littering among College-Aged Adults in the United States. International journal of environmental research and public health. 2022;19(13). Epub 20220701. doi: 10.3390/ijerph19138085 ; PubMed Central PMCID: PMC9265565. 35805745 PMC9265565

[42] United States Census Bureau. QuickFacts 2023. Available from: https://www.census.gov/quickfacts/fact/table/US/PST045222.

[43] Pierce JP, Shi Y, McMenamin SB, Benmarhnia T, Trinidad DR, Strong DR, et al. Trends in Lung Cancer and Cigarette Smoking: California Compared to the Rest of the United States. Cancer Prev Res (Phila). 2019;12(1):3–12. Epub 20181010. doi: 10.1158/1940-6207.CAPR-18-0341 ; PubMed Central PMCID: PMC7389269. 30305281 PMC7389269

[44] Timberlake DS, Wu J, Al-Delaimy WK. Tribal casinos in California: the last vestige of indoor smoking. BMC Public Health. 2012;12:144. Epub 2012/03/01. doi: 10.1186/1471-2458-12-144 ; PubMed Central PMCID: PMC3306736. 22364487 PMC3306736

[45] American Lung Association. Trends in Average Number of Cigarettes Smoked Per Day: American Lung Association,; 2024 [cited 2024 1/24/24]. Available from: https://www.lung.org/research/trends-in-lung-disease/tobacco-trends-brief/overall-tobacco-trends.

[46] Torkashvand J, Farzadkia M, Sobhi HR, Esrafili A. Littered cigarette butt as a well-known hazardous waste: A comprehensive systematic review. Journal of hazardous materials. 2020;383:121242. Epub 2019/09/29. doi: 10.1016/j.jhazmat.2019.121242 . 31563043

[47] Novotny TE, Hardin SN, Hovda LR, Novotny DJ, McLean MK, Khan S. Tobacco and cigarette butt consumption in humans and animals. Tobacco control. 2011;20 Suppl 1:i17–20. Epub 2011/04/29. doi: 10.1136/tc.2011.043489 ; PubMed Central PMCID: PMC3088460. 21504918 PMC3088460

[48] Gong M, Daniels N, Poppendieck D. Measurement of chemical emission rates from cigarette butts into air. Indoor Air. 2020;30(4):711–24. doi: 10.1111/ina.12648 31955455 PMC11005109

[49] Hiki K, Nakajima F, Tobino T. Causes of highway road dust toxicity to an estuarine amphipod: Evaluating the effects of nicotine. Chemosphere. 2017;168:1365–74. Epub 20161202. doi: 10.1016/j.chemosphere.2016.11.122 . 27919536

[50] Masoner JR, Kolpin DW, Cozzarelli IM, Barber LB, Burden DS, Foreman WT, et al. Urban Stormwater: An Overlooked Pathway of Extensive Mixed Contaminants to Surface and Groundwaters in the United States. Environmental science & technology. 2019;53(17):10070–81. Epub 20190821. doi: 10.1021/acs.est.9b02867 ; PubMed Central PMCID: PMC7370854. 31432661 PMC7370854

[51] Belzagui F, Buscio V, Gutiérrez-Bouzán C, Vilaseca M. Cigarette butts as a microfiber source with a microplastic level of concern. Sci Total Environ. 2021;762:144165. Epub 20201217. doi: 10.1016/j.scitotenv.2020.144165 . 33360456

[52] Moran K, Miller E, Mendez M, Moore S, Gilbreath A, Sutton R, et al. A Synthesis of Microplastic Sources and Pathways to Urban Runoff. Richmond, CA: San Francisco Estuary Institute, 2021.

[53] Campanale C, Massarelli C, Savino I, Locaputo V, Uricchio VF. A Detailed Review Study on Potential Effects of Microplastics and Additives of Concern on Human Health. International journal of environmental research and public health. 2020;17(4). Epub 20200213. doi: 10.3390/ijerph17041212 ; PubMed Central PMCID: PMC7068600. 32069998 PMC7068600

[54] Jackson SE, Tattan-Birch H, Shahab L, Beard E, Brown J. Have there been sustained impacts of the COVID-19 pandemic on trends in smoking prevalence, uptake, quitting, use of treatment, and relapse? A monthly population study in England, 2017–2022. BMC Medicine. 2023;21(1):474. doi: 10.1186/s12916-023-03157-2 38093317 PMC10720231

[55] Lam J, Schneider J, Shadbegian R, Pega F, St Claire S, Novotny TE. Modelling the global economic costs of tobacco product waste. Bull World Health Organ. 2022;100(10):620–7. Epub 20220822. doi: 10.2471/BLT.22.288344 ; PubMed Central PMCID: PMC9511662. 36188014 PMC9511662

