# Supplementary material for: Estimating the accumulation and re-accumulation of commercial tobacco, electronic cigarette, and cannabis waste based on a stratified random sample of census blocks
Source: PLoS One. 2025 Jan 6;20(1):e0313241. doi: 10.1371/journal.pone.0313241 (PMC11703088; doi:10.1371/journal.pone.0313241)
Supplement: S1 Table — (PDF) [file pone.0313241.s001.pdf]

Supplemental Material

S1. Index of socioeconomic characteristics derived from the 2015-2019 American Community Survey (ACS).

We relied on tract-level data from the 2015-2019 American Community Survey to characterize census blocks with respect to their socioeconomic status. We used a Principal component analysis with varimax rotation of Median Household Rent, Median Household Income, Median Home, Percent Unemployed, Percent Living in Poverty, and Percent Requiring Public Assistance. Factor loadings are shown in the table below. Areas with principle component scores below the mean were classified as “low” SES while areas at or below the mean were classified as “high” SES.

S1 Table. Principal component analysis of socioeconomic characteristics

Variable	Loading
Median Household Income	0.904
Median Rent	0.835
Percent Poverty	-0.816
Median Home Value	0.749
Percent Public Assistance	-0.661
Percent Unemployed	-0.553

Notes: Varimax rotation
