# Supplementary material for: Estimating the accumulation and re-accumulation of commercial tobacco, electronic cigarette, and cannabis waste based on a stratified random sample of census blocks
Source: PLoS One. 2025 Jan 6;20(1):e0313241. doi: 10.1371/journal.pone.0313241 (PMC11703088; doi:10.1371/journal.pone.0313241)
Supplement: S1 File — (PDF) [file pone.0313241.s002.pdf]

Protocol for Assessing Commercial Tobacco, Electronic Cigarettes, and Cannabis Waste

Georg E. Matt[1,2*], Lydia Greiner[1,2], Kristina Tran[2], Joseph Gibbons[2,3], Michael Vingiello[2], Paula Stigler Granados[2,4], Ronald Shadbegian[2,5], Thomas E. Novotny[2,4]

[1]Department of Psychology, San Diego State University, San Diego, California

[2]Center for Tobacco and the Environment, San Diego State University, San Diego, California

[3]Department of Sociology, San Diego State University, San Diego, California

[4]School of Public Health, San Diego State University, San Diego, California

[5]Department of Economics, San Diego State University, San Diego, California

March 22, 2024

Center for Tobacco and the Environment

Funded by California Tobacco-Related Disease Research Program Grant # T31IR1649

*Corresponding author: gmatt@sdsu.edu

Contents

Introduction....4
Project Summary....4
Protocol Summary....4
Part 1: Pre-Assessment....5
STEP 1: Prepare Data Collection File and Map....5
STEP 2: Validate Census Blocks and Apply Inclusion Criteria....5
STEP 3: Add Street Names and Labels to the Data Collection Form....6
STEP 4: Create and Complete an Assessment Form for Each Census Block....7
STEP 4: Determine Street Sweeping Schedule....8
STEP 5: Schedule Active Assessment and Data Collection....8
Part 2: Active Assessment....9
STEP 1: Create QuickCapture and Survey123 in ArcGIS....9
STEP 2: Set Up Equipment....9
STEP 3: Gather Equipment and Supplies for Active Assessment....9
STEP 4: Set Up iPad and Bad Elf Surveyor....10
STEP 5: Record Boundaries and Coordinates of REGULAR AREAS on the Census Block....10
STEP 6: Record Perimeter of IRREGULAR AREAS in the Census Block....11
STEP 7: Record Points of Interest ON THE BLOCK: Land Use Categories....11
STEP 8: Record Points of Interest ON THE BLOCK: Tobacco-Related Behaviors & City Services Categories....14
STEP 9: Record Points of Interest INSIDE THE BLOCK....14
STEP 9: Record Points of Interest ACROSS THE STREET....15
STEP 10: Schedule Field Data Collection....15
Part 3: Field Data Collection....16
STEP 1: Review the Types of TEC Waste You Will Collect....16
STEP 2: Create QuickCapture and Survey123 in ArcGIS....16
STEP 3: Gather Equipment and Supplies....16
STEP 4: Set Up iPad and Bad Elf Surveyor at the Census Block....17
STEP 5: Equip Team Members at Census Block....17
STEP 6: Find and Identify TEC Waste in Regular Areas....17
STEP 7: Record TEC Waste....19
STEP 8: Complete Assessment of Census Block....21
STEP 9: Quality Control....21
STEP 10: Collect TEC Waste in Irregular Areas....21

STEP 11: Prepare to Transport TEC Waste and Equipment 22
STEP 12: Weigh TEC Waste 22
STEP 13: Complete Field Data Collection 22
Part 4: Archival Data Collection 23
STEP 1: Determine Surface Area of Regular and Irregular Areas 23
STEP 2: Calculate Rainfall 30 Days Prior to Data Collection 24
STEP 3: Determine Date of Most Recent Street Sweeping 24
Part 5: Data Management 25
STEP 1: Upload and Clean Data 25
Part 6: Data Reporting 26
STEP 1: Describe Census Block Characteristics 26
STEP 2: Describe Survey Characteristics 27
STEP 3: Determine Count and Density of TEC Waste 27
Appendix A: Acronyms and Terms 29
Appendix B: Points of Interest 31
Appendix C: TEC Waste Identification Guide 37
Appendix D: Volunteer Training 46

Introduction

Project Summary

Commercial tobacco, electronic cigarette, and cannabis (TEC) products generate large amounts of single-use plastic waste and toxic pollutants that are largely discarded unmitigated in the environment after use. While community cleanups have shown this to be a significant problem, the development of scientific methods to assess and investigate the distribution of this TEC waste has been limited. This study aimed to: (1) develop research protocols for the identification, classification, geolocation, and collection of TEC waste in urban environments; (2) estimate the amounts and types of TEC waste based on a random sample of census blocks in southern California; and (3) statistically model a specific type of TEC waste (i.e., cigarette butts) as a function of characteristics of the census blocks. The study design called for a disproportionate, random sample of 60 census blocks from the 17,000 census blocks of the eight largest cities of San Diego County.

Protocol Summary

This protocol describes the methods used to identify, classify, geolocate, and collect TEC waste in urban neighborhoods in the eight largest cities in San Diego County. Within these neighborhoods, data were collected at the census block level, the smallest geographic area for which the U.S. Bureau of the Census provided basic demographic data. The protocol has six parts, which should be completed sequentially.

Prior to implementing this protocol, institutional licenses for ArcGIS, the geographic information system software, were secured, and project staff were trained in ArcGIS. The protocol was developed and pilot-tested over a 6-month period, and research assistants and volunteers were trained to use the protocol (See Appendix D).

Part 1: Pre-Assessment. These activities use online resources to determine if the census block meets inclusion criteria and can be assessed safely.

Part 2: Active Assessment. These activities are carried out in the field and include determining the boundaries of the areas to be assessed within the census block and the characteristics of the census block, such as land use and city services.

Part 3: Field Data Collection. These activities are carried out in the field and include locating, identifying, classifying, geolocating, collecting, and weighing TEC waste.

Part 4: Archival Data Collection: These activities use archival records to determine rainfall and street sweeping.

Part 5: Data Management. These activities use standard data management practices to verify and clean all data.

Part 6: Data Reporting. These activities describe the minimum data that should be included in a report on TEC waste in an urban environment.

Part 1: Pre-Assessment

The goals of the Pre-Assessment are:

- create a spreadsheet that lists the census blocks selected for assessment and their relevant characteristics;
- determine if each census block matches the inclusion criteria of the study;
- create the assessment form for each census block with street names and labels;
- determine the street sweeping schedule for each street in the census block.

To complete the Pre-Assessment, you will need:

- Laptop or desktop with internet access
- Access to ArcGIS and Google My Maps
- Access to a publicly available map of San Diego census blocks - https://www.arcgis.com/apps/mapviewer/index.html?layers=267d4ce87b0d43e09216109209273eb0
- A list of the selected census blocks with latitude and longitude

STEP 1: Prepare Data Collection File and Map

Use this step to create the spreadsheet to record relevant information about each census block and map all census blocks in the sample.

1. Create an Excel spreadsheet (file name: **DataCollectionPlan.xls**) with the following variables for each census block in the sample (n=60): (1) block ID, (2) longitude and latitude, (3) land use category, (4) SES, and (5) city. This spreadsheet also includes alternate census blocks (n=60). Additional variables will be added to this spreadsheet.
2. Create a Google My Maps of the selected census blocks and selected alternate census blocks.
    a. Using the latitude and longitude coordinates from the **DataCollectionPlan.xls,** create a shapefile in ArcGIS of the selected census blocks and selected alternate census blocks.
    b. Download the shapefile from ArcGIS (i.e., a .shp file) and upload it to Google My Maps (i.e., a .kml file). (Google My Maps name is **TPWRP Selected Census Blocks**): https://www.google.com/maps/d/edit?mid=1P0JFcPsWNDHzH7ISClvD83qmqSOdnEw&ll=32.88486476323801%2C-117.152482&z=10

STEP 2: Validate Census Blocks and Apply Inclusion Criteria

Use this step to confirm the census block borders and determine which census blocks meet inclusion criteria.

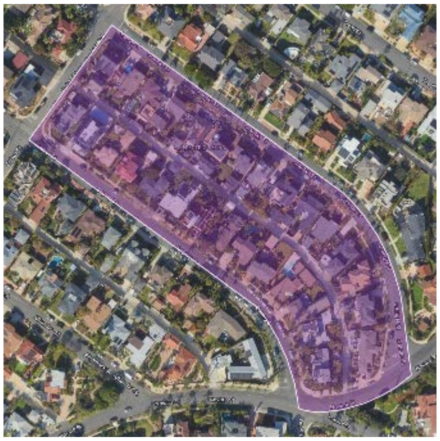

Fig. 1. Census block 115 meets inclusion criteria.

1. Validate each selected census block and alternate census block with a publicly available map of San Diego census blocks.
    a. Open the **DataCollectionPlan.xls** spreadsheet and locate each census block (n=60) on the **TPWRP Selected Census Blocks** My Maps.
    b. Confirm the census block borders on the San Diego census blocks map and the **TPWRP Selected Census Blocks** My Maps agree.
2. Inspect each selected census block and alternate census block to apply inclusion criteria. Inclusion criteria consider issues of safety and accessibility. (See Fig. 1 – Fig. 3.)
    a. Safety: Census blocks must have at least one <u>safe</u> walkable area on the perimeter. Most blocks have clear boundaries with perimeters following the shape of a publicly accessible street or an alley. (See Fig. 1 and Fig. 2 for examples of census blocks that meet criteria.) "Walkable" does not require

established sidewalks, but it does require sufficient distance from the roadway that pedestrians can safely walk (i.e., the edge of a freeway on-ramp is not "walkable").

b. Accessibility: Census blocks must have at least one publicly <u>accessible</u> walkable area on the perimeter. For census blocks that are located entirely on private property (i.e., open only to members or owners) or commercial property where paid admission is required (i.e., an amusement park or zoo), request permission from the property owner or managers to conduct assessments. If permission is not granted, replace this block with an alternate block. See Fig. 3 for an example of a census block that does not meet the criteria because it is not accessible.

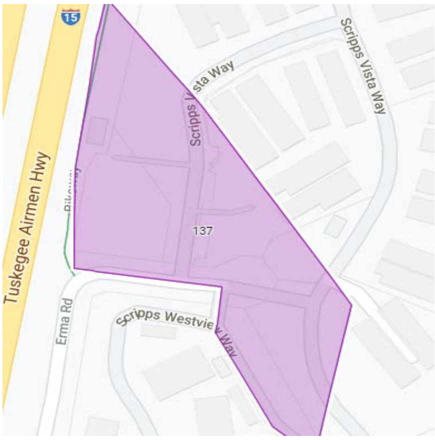

Fig. 2. Although two borders of this census block cut through private property, it meets inclusion criteria because its borders also include two streets and a bikeway, all of which are publicly accessible and walkable.

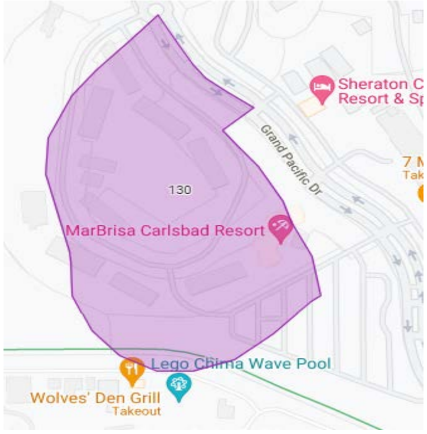

Fig. 3. This census block does not meet inclusion criteria. It is located on private property, and we could not obtain permission to collect data on this site. We selected an alternate block with the same land use and SES category.

3. Based on the inclusion criteria, choose an alternate census block if necessary. Document the need for an alternate block on the Data Collection Plan and select a new block from the list of alternates (i.e., select an alternate block with the same land use and SES categories).

STEP 3: Add Street Names and Labels to the Data Collection Form

Use this step to add the names of all publicly walkable, accessible streets on and within the perimeter of each census block to the **DataCollectionPlan.xls** spreadsheet and assign a letter to each street.

1. Open **DataCollectionPlan.xls** and add ten variables to the spreadsheet beginning with the letter A: "Street A," "Street B," "Street C," and so on.
2. Referring to **TPWRP Selected Census Blocks** My Maps, visualize a census block map. Enter the name of the northernmost street on the perimeter of the block (or most northwest) into the column labeled "Street A" on **DataCollectionPlan.xls.** Continue entering the names of perimeter streets into the spreadsheet in alphabetical order, moving in a clockwise direction. For example, if there are 4 streets, "Street A" would be to the north, "Street B" to the east, and so forth. See Fig.4.
    a. Treat alleys, bikeways, boardwalks or other publicly accessible walkways on the perimeter as if they were streets and assign a letter and name (i.e., "Alley" or "Bikeway").
    b. Assign any interior streets or alleys additional letters in a sequential fashion.

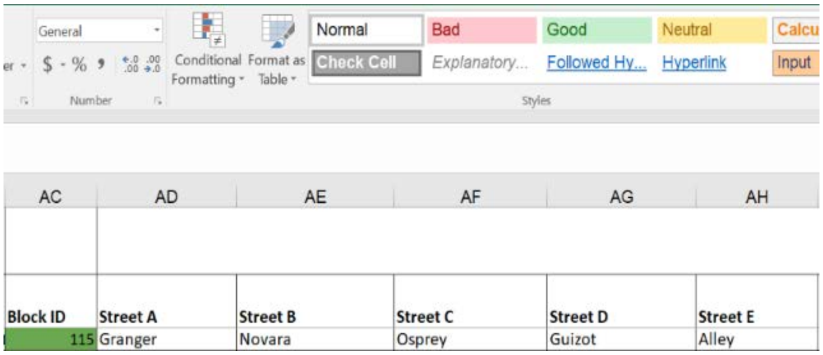

Block ID	Street A	Street B	Street C	Street D	Street E
115	Granger	Novara	Osprey	Guizot	Alley

Fig. 4. Street Letters and Names for Census Block 115

STEP 4: Create and Complete an Assessment Form for Each Census Block

Use this step to create and complete a form for each census block with information needed to collect data in the field.

1. Create a form for recording information needed to assess each census block. In the header of a word document, create fields for: (1) block ID, (2) navigation address, (3) street names, (4) parking options, (5) street sweeping information, and (6) additional notes. Name and save the document (**AssessmentForm.doc**). See Fig. 5.

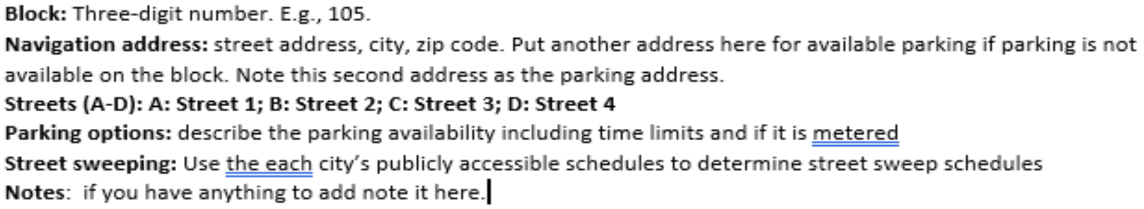
Block: Three-digit number. E.g., 105.
Navigation address: street address, city, zip code. Put another address here for available parking if parking is not available on the block. Note this second address as the parking address.
Streets (A-D): A: Street 1; B: Street 2; C: Street 3; D: Street 4
Parking options: describe the parking availability including time limits and if it is metered
Street sweeping: Use the each city's publicly accessible schedules to determine street sweep schedules
Notes: if you have anything to add note it here.

Fig. 5. **AssessmentForm.doc** header

4. To complete the Assessment Form for each census block:
   a. Open **AssessmentForm.doc.** Use **DataCollectionPlan.xls** to add the Block ID and street names to the Assessment Form. You will add the navigation address later in this step.
   b. Open **TPWRP Selected Census Blocks** My Maps and visualize the census block map. Take a screenshot of the census block map (Fig. 6.) and paste the screenshot onto **AssessmentForm.doc**. Orient the map so the north street is at the top of the page.
   c. Use **DataCollectionPlan.xls** to add street names and letters to each street on the screenshot. Place the textboxes outside the perimeter of the assessment area. (Fig. 7.)

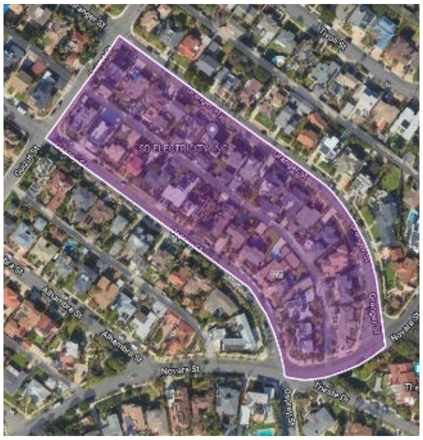

Fig. 6. Census block 115 screenshot from **TPWRP Selected Census Blocks**.

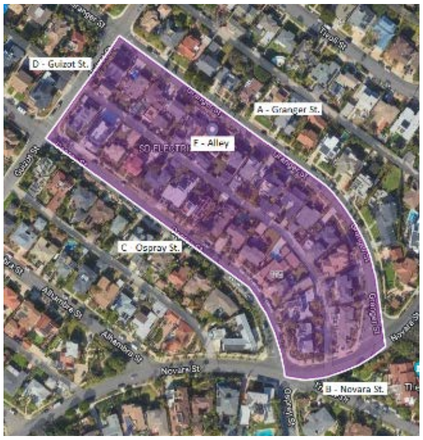

Fig. 7. Census block 115 screen shot with street names and labels.

d. Use **TPWRP Selected Census Blocks** My Maps to determine the intersection in the northernmost corner of the census block. Use the address of one building in this intersection as the navigation address for the research team when they travel to the site. Add navigation address to the **AssessmentForm.doc** and record it in the **DataCollectionPlan.xls**.

e. Save the **AssessmentForm.doc** by adding the BlockID to the file name (e.g., Block115_AssessmentForm.doc).

f. Print a copy of the census block's **AssessmentForm.doc** and highlight the perimeter streets and any interior streets or alleys using a yellow highlighter. Highlight the perimeter of any other public areas inside the perimeter of the census block (e.g., public parking areas, shopping centers, restaurants) using a red highlighter. (See Fig. 8.). Store in binder.

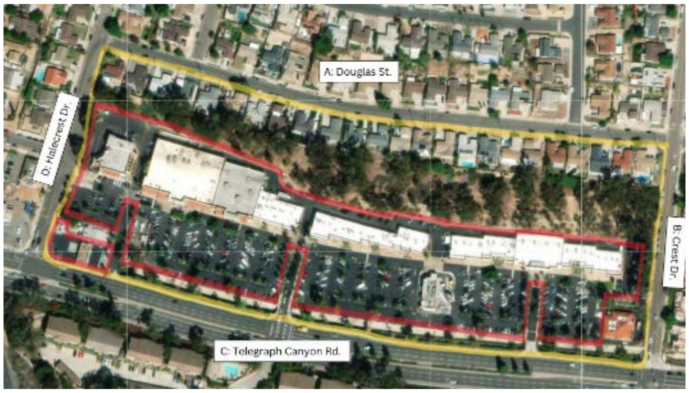

Fig. 8. Screenshot of Google Map of Census Block 107 with perimeter streets highlighted in yellow and interior public areas highlighted in red.

STEP 4: Determine Street Sweeping Schedule

Use this step to determine and record the street sweeping schedule for each street in each census block.

1. Obtain street sweeping schedule from municipal websites or observation of posted street sweeping schedules. For example: Both sides of Granger Street are swept on the 4th Wednesday of even months. See Fig. 9.
2. For each street in the census block, record the frequency and day(s) of street sweeping on the side of the street to be assessed in the **DataCollectionPlan.xls**. Add this information to the hard copy of the **AssessmentForm.doc** for each census block.

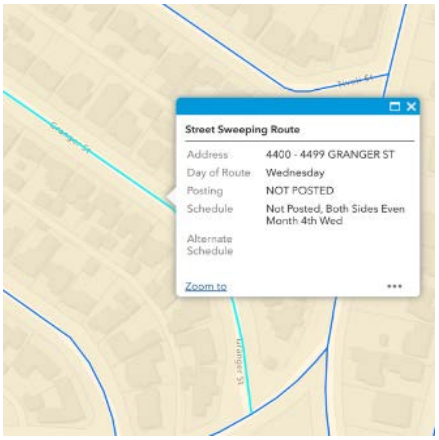

Fig. 9. Both sides of this street are swept the 4th Wednesday of every even month.

STEP 5: Schedule Active Assessment and Data Collection

Use this step to schedule active assessment and data collection, accounting for street sweeping.

1. Consult street sweeping schedule to determine best date(s) for Data Collection, and then schedule Active Assessment accordingly.
2. Schedule the data collection to maximize the number of days between street sweeping and collection date.
3. Schedule active Assessment 1 week prior to Data Collection.

Part 2: Active Assessment

Active Assessment takes place a few days before the collection of TEC waste. Conduct Active Assessment of the census block to confirm the areas to be surveyed and to determine the location of "points of interest" in the census block, such as tobacco retailers, bus stops, and storm drains. The goals of Active Assessment are:

- confirm the regular and irregular areas of the census block;
- collect GPS coordinates of all regular areas on the perimeter and within the census block;
- record perimeters of irregular areas within the census block; and
- record points of interest within and adjacent to the census block.

At the conclusion of the Active Assessment, schedule the census block for Data Collection (See Part 3). Data Collection should be completed within 5 days of Active Assessment.

STEP 1: Create QuickCapture and Survey123 in ArcGIS

Use this step to create the user interface in ArcGIS to record coordinates and points of interest. Overall points of interest categories (e.g., single-family housing, health care) are recorded in QuickCapture; subcategories are recorded in Survey123. See Appendix B for a detailed description of categories and subcategories.

1. Determine a list of categories and subcategories of points of interest to be collected. This decision should be driven by the research question and the time and resources available. Create a survey in QuickCapture to record the overall category and geolocation of each point of interest. (See Fig. 14.)
    a. Add the camera icon to those categories where a photo is to be collected (e.g., health care, multiunit housing).
    b. Do not add a camera icon to categories where photos should not be collected (e.g., people actively smoking, and single-family homes) due to privacy issues.
2. Create a survey in Survey123 to record subcategories. See Fig. 15 - Fig. 20.

STEP 2: Set Up Equipment

Use this step to install ArcGIS software needed to record data in QuickCapture and Survey123 and to pair the iPads with GPS Surveyors that will be used to improve the accuracy of geolocation. (This step will be done only one time.)

1. Following manufacturer's instructions, install ArcGIS QuickCapture and Survey123 on each iPad that will be used for data collection.
2. Following manufacturer's instructions, pair each Bad Elf Surveyor (Bad Elf GNSS Surveyor https://bad-elf.com/products/be-gps-3300) with an iPad. Place a piece of tape on the back of the Surveyor and iPad and label them with matching ID #s. This will make it easy to identify the pairs in the field.
3. Charge the iPads and Bad Elf Surveyors.
4. Following manufacturer's instructions, attach the clamp bracket for iPad surface mount to the monopod. The monopod should extend a total of 68". The clamp bracket should be attached to the monopod 34" above the ground.

STEP 3: Gather Equipment and Supplies for Active Assessment

Use this step to gather all equipment and supplies that will be needed to complete the Active Assessment in the field.

1. iPads (2) with QuickCapture and Survey 123 installed. Be sure iPad is fully charged
2. GPS Surveyors (2), also known as Bad Elf Surveyors. Be sure Surveyors are fully charged.
3. Monopods (2) fitted with a clamp bracket for iPad surface mount
4. Rolling cart with supplies:
    a. Two copies of the **AssessmentForm.doc** on clipboards.
    b. Writing instruments: colored pens, markers, and highlighters
    c. Orange safety vests

d. Box of nitrile gloves
e. Cleaning supplies (75% alcohol wipes, paper towels, trash bag)
f. Hand sanitizer
g. Depending on weather conditions: Water, sunscreen, snacks, hats.

STEP 4: Set Up iPad and Bad Elf Surveyor

Use this step to set up equipment in the field.

1. Check the labels on the iPad and Bad Elf Surveyor to make sure they are paired (i.e., have matching IDs). The iPad will not be able to "find" the Bad Elf Surveyor if the two devices are not paired.
2. Turn the Surveyor on and unlock the iPad.
3. Open QuickCapture on the iPad. QuickCapture should "find" the Bad Elf Surveyor. (Consult manufacturer's instructions to trouble-shoot any problems with pairing these devices.)
4. Fully extend the legs of the monopod. Be sure to do this every time to have a consistent distance of 40" between the iPad and the ground.
5. Place the Surveyor between the two clamps on the top of the monopod. Clip the Surveyor's lanyard to the lanyard on the top of the monopod for extra security.
6. Place the iPad inside the four clamps on the iPad surface mount on the monopod. Use caution: If the iPad's protective case is very thick, it may need to be removed to fit the iPad into the iPad may need to be removed from its protective case to fit into the monopod clips. Be careful not to drop it! (Fig. 10.)

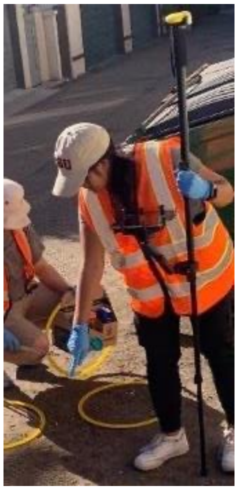

Fig. 10. iPad and Bad Elf on monopod.

STEP 5: Record Boundaries and Coordinates of REGULAR AREAS on the Census Block

Use this step to collect coordinates of "regular areas" in each census block. Regular areas are: (1) the space from the inside edge of the sidewalk to the gutter, including the curb strip (the landscaped area between the edge of the sidewalk and curb) along the perimeter of the census block and any streets within the perimeter, and (2) any alleys within the perimeter, which are measured from "edge to edge". Coordinates will be used to determine the surface areas area for which TEC waste will be assessed. Before beginning Step 5, refer to the **AssessmentForm.doc** to orient yourself to the streets to be included. You will begin on Street A and proceed in a clockwise fashion to determine boundaries and record coordinates (e.g., in a census block with 4 streets on the perimeter, you will begin on Street A, continue to Street B, then Street C, and end on Street D).

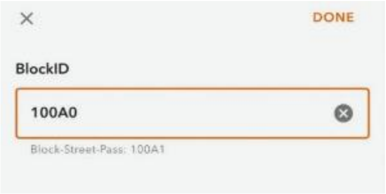

Fig. 11. BlockID, Street Letter, and Pass Number on QuickCapture screen.

1. Open QuickCapture and select your project.
2. Enter the BlockID, Street Letter, and Pass Number. [NOTE: For Active Assessment, the Pass Number is always 0.] See Fig. 11.
3. Check the accuracy of the GPS shown on the bottom of the iPad screen. Ideally, the GPS accuracy should be 3 feet or less.
4. Determine the "beginning" of Street A. Use permanent objects as reference points, such as the ADA Yellow Warning mats at most street corners, to distinguish between the beginning of one street and the end of another. Record reference points on the **AssessmentForm.doc** with any notes to assist on day of data collection (e.g., "end of street A is at end of red curb"). [NOTE: When indicating "left" or "right" always orient yourself facing away from the interior of the census block.] (See Fig. 12.)
5. At the beginning of Street A, place the monopod at inner edge of the sidewalk, and use QuickCapture to take a coordinate (inner coordinate).

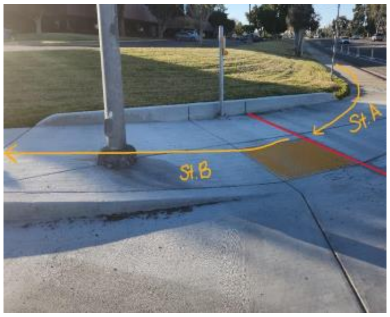

Fig. 12. Reference points mark end of Street A and start of Street B.

Walk to the edge of the curb and place the monopod at the edge of gutter. If there is no demarcation (e.g., the gutter is concrete and the street is blacktop) to indicate the edge of the gutter, estimate approximately 18 inches from the curb and use QuickCapture to take a coordinate (outer coordinate). [NOTE: Placement of the monopod should correspond to the reference points for Street A that are recorded on **AssessmentForm.doc**.]

6. Walk to the other end of Street A and repeat #5 and #6. The shape of the street will be visualized online using ArcGIS using these four coordinates unless the street is irregular. Take additional coordinates if:
    a. There are any unusual borders (e.g., sidewalk does not extend the length of the street). In this case, take a pair of coordinates at the beginning and end of the street and another pair of coordinates at the beginning and end of an unusual border. Record on the census block's **AssessmentForm.doc**.
    b. Areas inside the inner edge of the sidewalk are used by pedestrians, i.e., "functional sidewalk" (Fig. 13). Take a pair of coordinates at each end of functional sidewalk. Record on the census block's **AssessmentForm.doc**.
7. After all coordinates are taken for street A and their placement is recorded on the **AssessmentForm.doc** marked the paper map, you are ready to start street B.
8. In QuickCapture, change the street from A to B and enter pass 0.
9. Repeat # 5-7 for each street.
10. If you find another "street" inside the census block, such as an alley that was not noticed on the Pre-Assessment, assign the next street letter, note it on the **AssessmentForm.doc**, and take coordinates.
11. NOTE: Edit the census block's **AssessmentForm.doc** to add/delete irregular areas (e.g., private/public parking lot designation incorrect on Pre-Assessment).

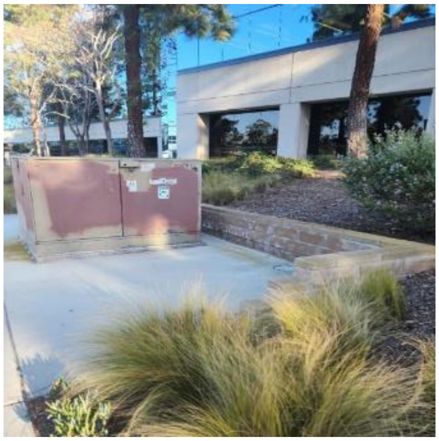

Fig. 13. Functional sidewalk around a utility box in inside edge of sidewalk.

STEP 6: Record Perimeter of IRREGULAR AREAS in the Census Block

Use this step to collect the perimeter of all "irregular areas" inside the census block. Irregular areas are any other public space inside the perimeter, such as parking lots, shopping plazas, or other businesses that are open to the public.

Before beginning Step 6, refer to the **AssessmentForm.doc** to orient yourself to the location of irregular areas, which are outlined in red on the map. Edit the **AssessmentForm.doc** to include any additional irregular areas noted in Step 5.

1. Starting on Street A, number each irregular area on the **AssessmentForm.doc** beginning with #1. Continue in a clockwise manner until all irregular areas have been numbered.
2. Start with Irregular Area #1, use QuickCapture to record a polygon of the irregular area while walking along the perimeter. Record continuously from the start until you return to the starting point.
3. Place a checkmark next to the # to indicate the polygon has been recorded.
4. Repeat #2 and #3 until a polygon of each irregular area has been recorded.

STEP 7: Record Points of Interest ON THE BLOCK: Land Use Categories

We record three types of Points of Interest: Land Use, Tobacco-Related Behaviors, and City Services. To improve accuracy, collecting Points of Interest is divided into two parts. Use Step 7 to record Land Use, or how the buildings on and adjacent to the census block are used. Use Step 8 to record Tobacco-Related Behaviors and City Services.

There are 12 categories of Land Use, shown in purple on the QuickCapture screen in Fig. 14. Six Land Use categories have subcategories shown on the Survey123 screens in Fig. 15 – Fig. 20.

You will use QuickCapture to record categories and Survey 123 to record subcategories. Points of Interest ON THE BLOCK are recorded on the perimeter and on streets and irregular areas (e.g., shopping centers) inside the census block.

1. In QuickCapture, enter the block ID (100-160), street letter (A-[unknown]), and pass number (0); press done. [For Active Assessment, the pass number is always 0.]
2. Open QuickCapture "Points of interest ON THE BLOCK" (See Fig. 14.)
3. Check the accuracy of the GPS shown on the bottom of the iPad screen. Ideally, the GPS accuracy should be 3 feet or less.
4. Starting at the northwest corner of street A, walk toward street B. When you reach the first structure, select the land use category that best describes it. The land use category is not always immediately apparent. You may need to Google search the building/business name to select the best category.
5. Select from one of the following categories (See Appendix B for more detail):
    a. Single-family housing: One unit.
    b. Multi-unit housing: Two or more units. Check mailboxes to be sure to correctly identify small multi-unit structures (i.e., a single-family home converted to a duplex)
    c. Parks and Recreation: Public or private parks, pools, picnic areas, hiking trails, or similar.
    d. Mixed-use: One building that contains different types of businesses <u>that you cannot categorize</u> (e.g., a large office building with no publicly accessible directory and no business signs). If you can categorize some of the businesses in the building, record all those you can and use "mixed use" to indicate there are other businesses that you cannot categorize. If you can categorize all the businesses in the building, record all the businesses--do not use "mixed use."
    e. Manufacturing: Industrial production.
    f. Parking lot: Public or private parking, including both indoor parking structures and outdoor lots.
    g. Health care: Clinics, medical provider offices, hospitals, behavioral health centers, and other related such as physical therapy.
    h. Services: Personal care, professional services, and trade.
    i. Retail: Grocery, department or specialty shop, convenience, tobacco retailer, alcohol.
    j. Education: Preschool, K-12, post-high school trade or college
    k. Hospitality: Hotel, bar, restaurant, coffee shop, gym, entertainment venues.
    l. Other: Construction, vacant lot, vacant building.
6. Click on the category. For all categories except single-family home, you will take a photo of the structure. <u>Do not take photos of single-family homes</u>. To take a photo or coordinate, stand on the sidewalk opposite the entrance to the structure. If there is no entrance, use the center of the structure.
7. Some categories require additional information. The following categories require selection of subcategories, shown in Fig. 15 – Fig. 20: (1) health care, (2) services, (3) retail, (4) education, (5) hospitality, and (6) other. Click on the category and to open the Survey 123 form. Take a photo and record the subcategory in Survey123.
8. When you have completed and saved Survey123, you will be re-directed to QuickCapture.
9. If the structure is mixed-use (i.e., multiple types in one building) search for a building/plaza sign with business names. Use a photo of the plaza sign to record each business in the mixed-use structure.
10. If the structure is located on a corner (e.g., on the corner of Street A and Street B, you will record this structure twice, once on Street A and once on Street B, following the procedure above.
11. If the structure is located between two streets, or between a street and an alley (e.g., the front of the home is on Street A and the back of the home is on an alley—Street E) you will record the structure twice, once Street A and once on Street E, following the procedure above.
12. After all Land Use categories have been recorded for street A, you are ready to start street B.
13. In QuickCapture, change the street from A to B and enter pass 0.
14. Repeat #4—12 for each street.

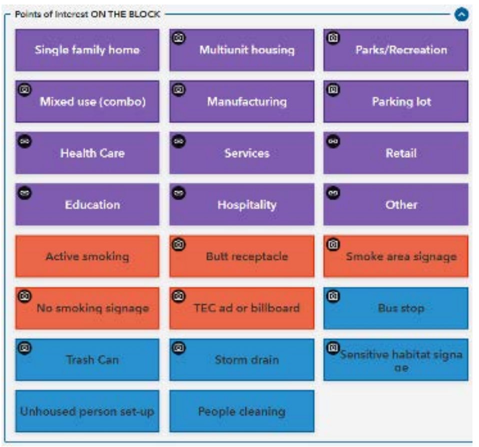

Fig. 14. QuickCapture screen with Land Use Categories (n=12) shown in purple.

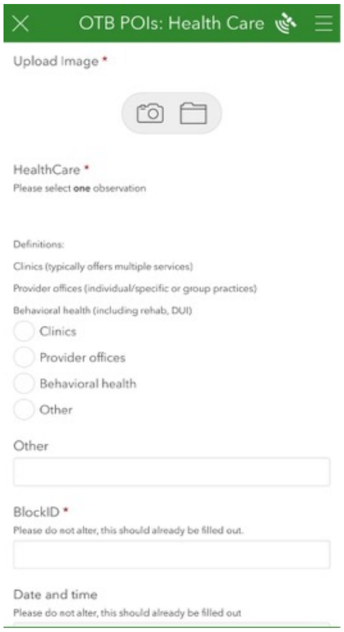

Fig. 15. Health care subcategories in Survey123.

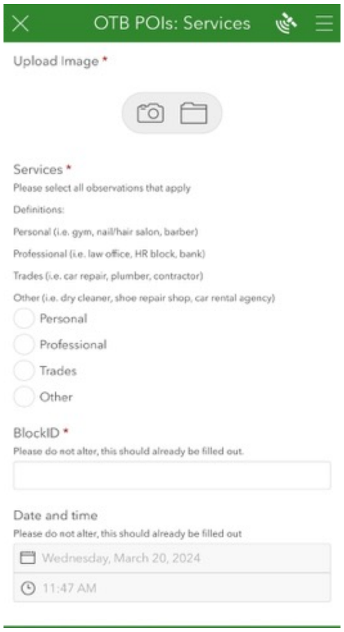

Fig. 16. Services subcategories in Survey123.

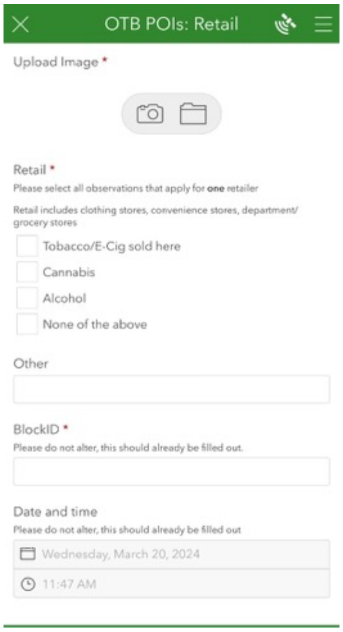

Fig. 17. Retail subcategories in Survey123.

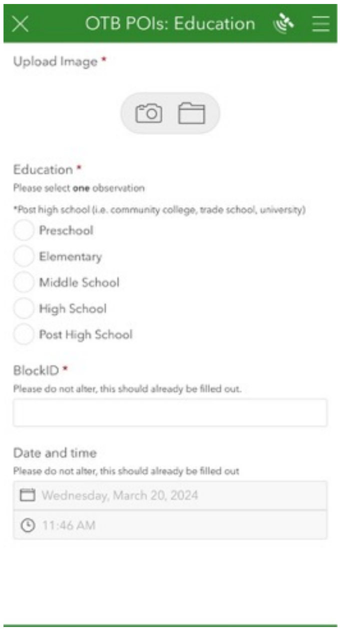

Fig. 18. Education subcategories in Survey123.

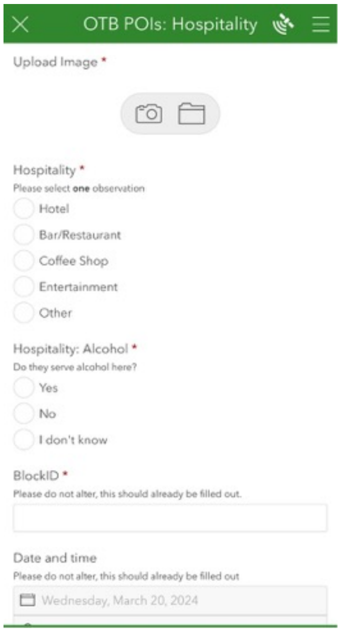

Fig. 19. Hospitality subcategories in Survey123.

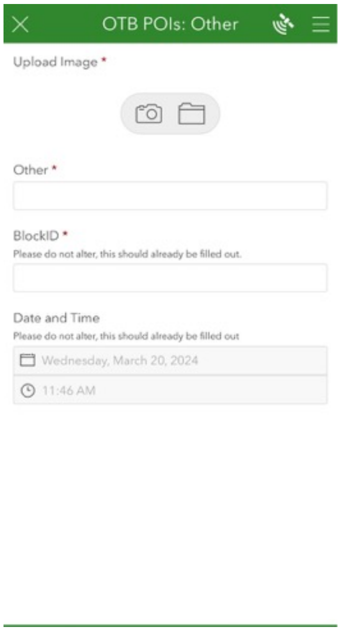

Fig. 20. Other subcategories in Survey123.

STEP 8: Record Points of Interest ON THE BLOCK: Tobacco-Related Behaviors & City Services Categories

Use this step to record two types of points of interest: tobacco-related behaviors (shown in red on Fig. 14) and city services (shown in blue on Fig. 14) on the block.

1. After you have recorded the Land Use categories on each street, you are ready to record Tobacco-Related Behaviors and City Services categories in QuickCapture.
2. Starting at the northwest corner of street A, walk toward street B. Observe for Tobacco-Related Behaviors and City Services. When you reach the first example, select the category that best describes it. All categories require a photograph except those that include people. Do not photograph people.
3. Select from one of the following categories (See Appendix B for more details):
    a. Tobacco-Related Behaviors (Shown in red on Fig. 14)
        i. Active smoking: Anyone observed smoking or vaping on the street or in their car. Enter one observation for each person (i.e., if a group of 3 people smoking, enter 3 observations). Do not photograph people.
        ii. Butt receptacle: A designated receptacle for discarding smoked TEC products located on public space. Do not include metal trashcans.
        iii. Smoking Area signs: Designated smoking areas in a public space or on a building that faces onto public space (e.g., faces onto a parking lot or sidewalk).
        iv. No Smoking signs: Signs that prohibit smoking in a public space or on a building that faces onto public space (e.g., faces onto a parking lot or sidewalk).
        v. TEC Advertising: Advertising in a public space or on a building that faces onto public space (e.g., faces onto a parking lot or sidewalk). Include billboard advertising that is visible from the census block.
    b. City Services (Shown in blue on Fig. 14)
        i. Bus stops: Regular bus stop marked by a posted sign. May contain a bus shelter and/or a bench. Take photograph from sidewalk facing the center of the bus stop.
        ii. Public trash can: Trash cans designed for public use; usually placed and maintained by City. Include trashcans placed in public areas by business or property owners (e.g., a trash can placed outside a market for patrons to use). Do not include dumpsters or household trash cans. Exception: Include dumpsters if they have been placed by City to maintain city streets (e.g., in an area with known encampments).
        iii. Storm drains: Marked by City as storm drains. Include unmarked storm drains in parking lots and alleys.
        iv. Sensitive habitats: Posted signs in Coastal California to mark designated protected areas.
        v. Encampments: Any space where unhoused persons (or person) are living temporarily on public areas. Include cars. Enter one observation, no matter how many people are in the encampment. Do not take any photographs.
4. After all Tobacco-Related Behaviors and City Services categories have been recorded for street A, you are ready to start street B.
5. In QuickCapture, change the street from A to B and enter pass 0.
6. Repeat #3—5 for each street.

STEP 9: Record Points of Interest INSIDE THE BLOCK

Use this step to record points of interest in any irregular areas inside the block. <u>This step is only necessary if there are irregular areas inside the block.</u>

1. After Points of Interest on all streets on the perimeter and inside the census block have been recorded in QuickCapture and Survey123, you are ready to collect Points of Interest INSIDE THE BLOCK.

2. Walk to irregular area #1 (Consult **AssessmentForm.doc**.) Using the street letter of the street adjacent to the irregular area, enter the BlockID, Street Letter, and Pass Number. [For Active Assessment, the pass number is always 0.] If the irregular area is on a corner of two streets on the census block, use the street letter for either street.
3. Open QuickCapture "Points of interest ON THE BLOCK" (See Fig. 14.)
4. Check the accuracy of the GPS shown on the bottom of the iPad screen. Ideally, the GPS accuracy should be 3 feet or less.
5. Walk the edge of the irregular area. When you reach the first structure, select the land use category that best describes it.
6. Repeat Step 7 #4—11 to record Land Use categories and subcategories.
7. Repeat Step 8 #1—3 to record Tobacco-Related Behaviors and City Services.
8. After you have recorded Points of Interest for the first irregular area, you are ready to repeat #5—#7 for any additional irregular areas inside the census block.
9. When you have recorded Points of Interest on all irregular areas inside the census block, you are ready to record Points of Interest ACROSS THE STREET.

STEP 9: Record Points of Interest ACROSS THE STREET.

Use this step to record points of interest on the opposite side of each perimeter street on the census block.

1. Enter the block ID (100-160), street letter (A-[unknown]), and pass number (0); press done. [For Active Assessment, the pass number is always 0.]
2. Open QuickCapture "Points of interest ACROSS THE STREET"
3. Starting at the northern corner of Street A, walk across the street.
4. Start at the corner of the sidewalk (or walkway) that is directly across the street from sidewalk assessed on Street A and walk toward Street B.
5. Repeat Step 7 and Step 8 to collect Points of Interest located on the sidewalk ACROSS THE STREET on Street A. Include only Points of Interest that are located on the sidewalk (i.e., if there is a parking lot of a shopping center adjacent to the sidewalk ACROSS THE STREET include only the parking lot, not the shops).
6. Include the sidewalk (or walkway) that is diagonally across the street from the intersections on the borders of the census block (e.g., include the sidewalk that is diagonally across the street from the intersection of the sidewalk on Street A and Street B.)
7. When you reach the end of Street A (including the corner of the sidewalk that is diagonally between A and B), you are ready to start street B.
8. In QuickCapture, change the street from A to B and enter pass 0.
9. Repeat # 2-8 until all streets are completed.

STEP 10: Schedule Field Data Collection.

Use this step to schedule Field Data Collection of TEC waste.

1. Record date the Active Assessment was completed in **DataCollectionPlan.xls**
2. Set the date and time of Field Data Collection no more than one week after Active Assessment.

Part 3: Field Data Collection

The goals of Field Data Collection are to:

- identify TEC waste in the assessment areas within the census block;
- photograph, categorize, count, and weigh TEC waste found within the census block; and
- record GPS coordinates of TEC waste using QuickCapture and Survey123.

STEP 1: Review the Types of TEC Waste You Will Collect

Use this step to learn about the categories of TEC waste that will be collected and determine the time and resources available to collect it.

1. Review the TEC Waste Guide for an understanding of the range of products that are included in each category (n=6) of TEC waste (Appendix C).
2. Decide on your research question and assess the time and resources available. If resources and time are limited,
   a. Consider reducing the # of categories of waste collected.
   b. Do not consider revising the definitions of the categories of waste.

STEP 2: Create QuickCapture and Survey123 in ArcGIS

Use this step to create the user interface in ArcGIS to record TEC waste.

1. Following manufacturer's instructions, create a survey in QuickCapture to record each observation of TEC waste (i.e., category, geolocation, and #). See Fig. 21.
2. Create a survey in Survey123 to record total weight of TEC waste by type and street.

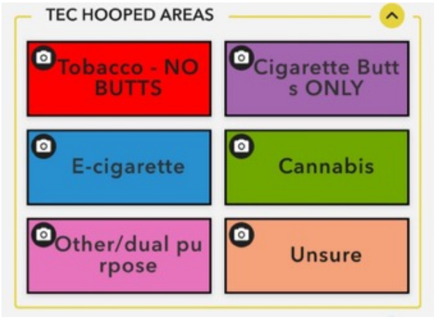

Fig. 21. TEC waste categories in QuickCapture

STEP 3: Gather Equipment and Supplies

Use this step to gather all equipment and supplied needed to complete Field Data Collection. NOTE: This Step assumes one team or 3-4 persons to observe, record, and pick up TEC waste. The list of equipment and supplies is sufficient for 1 team. Extra equipment (e.g., iPad, Surveyor) is included in case of malfunction.

1. iPads (2) with QuickCapture and Survey 123 installed. Be sure iPad is fully charged.
2. GPS Surveyors (2), also known as Bad Elf Surveyors. Be sure GPS Surveyors are fully charged.
3. Monopods (2) fitted with a clamp bracket for iPad surface mount
4. Rolling cart with collection supplies:
   a. Two copies of the census block's **AssessmentForm.doc** on clipboards
   b. Writing instruments: colored pens, markers, and highlighters
   c. Long tweezers and grabbers (1 per team member)
   d. Rakes (2)
   e. Hoops (10)
   f. Copy of TEC Waste Pocket Guide
   g. Plastic containers in a cardboard carrier (Fig. 22). Label one set of containers per street with Street Letter and six categories of TEC waste (e.g., Street A, Butts Only):
      i. Cigarette Butts Only
      ii. Tobacco Products and Packaging (No Butts)
      iii. E-Cigarette Products and Packaging
      iv. Cannabis Products and Packaging
      v. Multiple Use
      vi. Unsure

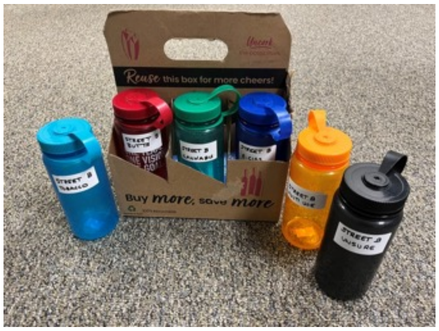

Fig. 22. Individual TEC waste collection containers in cardboard carrier.

5. 5-gallon plastic food storage bucket with airtight lid (2 per team)
6. Nitrile gloves (2 boxes per team)
7. Trash bags for general litter
8. Safety vests
9. First aid kit
10. Cleaning supplies in a 5-gallon plastic food storage bucket:
    h. Spray bottles with 70% isopropyl alcohol
    i. Hand sanitizer
    j. Paper towels
    k. Nitrile Gloves
    l. Trash bags

STEP 4: Set Up iPad and Bad Elf Surveyor at the Census Block

Use this step to set up equipment in the field.

1. Check the labels on the back of the iPad and Bad Elf Surveyor to make sure they are paired (i.e., have matching IDs). The iPad will not be able to “find” the Bad Elf Surveyor if the two devices are not paired.
2. Turn the Surveyor on and unlock the iPad.
3. Open QuickCapture on the iPad. QuickCapture should “find” the Bad Elf Surveyor. (Consult manufacturer’s instructions to trouble-shoot any problems with pairing these devices.)
4. Fully extend the legs of the monopod. Be sure to do this every time to have a consistent distance of 40” between the iPad and the ground.
5. Place the Surveyor between the two clamps on the top of the monopod. Clip the Surveyor’s lanyard to the lanyard on the top of the monopod for extra security.
6. Place the iPad inside the four clamps on the iPad surface mount on the monopod. Use caution: If the iPad’s protective case is very thick, it may need to be removed to fit the iPad into the iPad may need to be removed from its protective case to fit into the monopod clips. Be careful not to drop it! (Fig. 10.)

STEP 5: Equip Team Members at Census Block

Use this step to prepare the team members for data collection.

1. Identify tasks of each team member. Each team needs one person to be responsible for photographing and one for categorizing TEC waste. If there are multiple teams, assign each team to a different area and record boundaries on the map on the **AssessmentForm.doc.**
2. Distribute equipment and supplies. Each team needs an iPad and Surveyor on a monopod, a rolling cart with collection supplies, and a 5-gallon bucket for trash; each team member needs an orange vest and nitrile gloves.

STEP 6: Find and Identify TEC Waste in Regular Areas

Use this step to ensure that the entire surface of the assessment area is inspected, and TEC waste is found and identified.

1. Put on nitrile gloves. Make sure to have extra gloves to replace as necessary (torn, soiled). Do not touch TEC waste without wearing gloves and using a grabber.
2. Open QuickCapture, select your project, and enter the BlockID, Street Letter, and Pass Number (1); press done. [For Data Collection, the pass number is always 1.]
3. Check the accuracy of the GPS shown on the bottom of the iPad screen. Ideally, the GPS accuracy should be 3 feet or less.
4. Locate the northwest corner of Street A on the map on the **AssessmentForm.doc**.
5. Walk to the northwest corner of Street A. Begin observing at the border of Street A marked on the map on the **AssessmentForm.doc.**
6. Each team member observes for TEC waste as they slowly walk toward Street B (move in a clockwise direction), using either the “lawnmower” or “snowplow” method to ensure that TEC waste is not missed.

   a. Lawnmower: When using the lawnmower method, one person walks the length of the area being assessed, makes a close turn at the end, and walks back to the starting point parallel to the first pass, as one would when mowing the lawn. See Fig. 23.
   b. Snowplow: When using the snowplow method, each person is responsible for an area no more than 3 feet wide, directly in front of them. Several people can walk side-by-side, staying “in their lane,” observing the area directly in front of them. See Fig. 24.

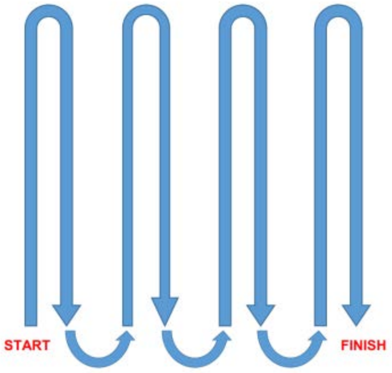

Fig. 23. Lawnmower Method. San Francisco Estuary Institute. https://www.sfei.org/sites/default/files/biblio_files/Trash%20Monitoring%20Playbook%202021%20rev2.pdf

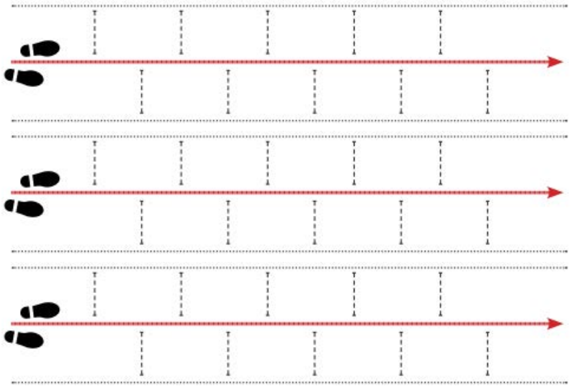

Fig. 24. Snowplow Method. San Francisco Estuary Institute. https://www.sfei.org/sites/default/files/biblio_files/Trash%20Monitoring%20Playbook%202021%20rev2.pdf

7. Use snowplow or lawnmower method. Depending on the width of the regular area, assign three (or more) staff to inspect a specific portion (e.g., sidewalk, curb strip, and gutter). If there are insufficient staff to use the snowplow method (i.e., area is too wide), use lawnmower method instead.
8. Observe from the inside edge of the sidewalk to the edge of the outside edge of the gutter where the concrete gutter meets the street surface. See Fig. 25.
   a. The inside edge of the sidewalk may have a hard boundary (e.g., private homes or property, or abutting an irregular area), or may extend further into functional sidewalk (e.g., a small walkable area used by pedestrians). Include TEC waste that is in the crack between the inside edge of the sidewalk and the boundary.

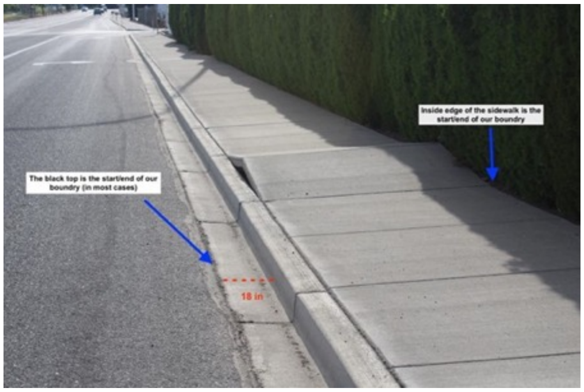

Fig. 25. Observe from inside edge of sidewalk to point where gutter meets the street surface. If there is no line indicating where the gutter meets the street surface, use a hoop to determine the boundary.

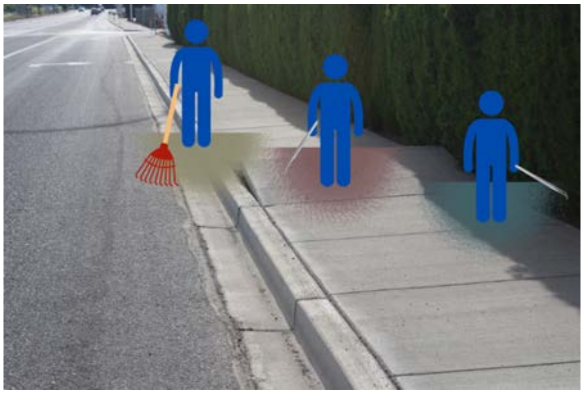

Fig. 26. A team of three using the snowplow method to observe the edge of the sidewalk to the edge of the gutter. Rake being used to remove debris from gutter and cracks in the paving.

b. The edge of the gutter is the line where the street surface (blacktop or concrete) meets the surface of the gutter (approximately 18”). If a storm drain extends beyond the edge of the gutter, it is included in the assessment area. If there is no line between the street surface and the gutter, use a hoop to determine the boundary.
c. Anything outside of these boundaries (even 1”), should not be counted. Adhering to this rule is critical for the safety of RAs, respect for private property, and data collection reliability.

9. Clear the area to remove debris. See Fig. 26.
    a. Use a rake to go through any fallen leaves or dirt in the gutter or on the sidewalk. Rake the debris into a thin layer and inspect it to see litter if any is there.
    b. Do not use a rake to remove fallen leaves or debris on landscaped curb strips. If a curb strip has mulch, rocks, plants, etc. in it, do not rake.
    c. Use tweezers or rakes to scrape debris out of the cracks in the concrete and inspect.
    d. If there are plants covering parts of the assessment area (e.g., ground cover extending out over the edge of the sidewalk), use a rake, grabber, or tweezers to gently lift the plants and check underneath.
10. As you walk, carefully look at the ground (area) directly in front of you. Pay close attention. Scan the area for litter of any type.
11. I nspect all the litter. Determine if:
    a. <u>It is obviously not TEC waste</u> (e.g., water bottle, food wrappers) pick it up and deposit it in the trash bucket.
    b. <u>It appears to be TEC waste</u> (e.g., a cigarette butt), visually inspect it from a close range. Some “obvious” TEC waste products turn out to be small stones, shells, leaves, nuts, or sticks. You may have to pick up the product to inspect it closely. To inspect closely:
        i. Use tweezers or grabbers to pick up the litter.
        ii. Examine it for text, logos, etc., to determine if it is, or could be, a piece of TEC waste. Some pieces are quite small!
        iii. If logos or brands are unfamiliar, use Google to help identify the product.
        iv. If you determine it is not TEC waste, discard it in a trash bucket.
        v. If you determine it is TEC waste, place it back on the ground where you found it.
        vi. Once you confirm it is TEC waste, place it back where you found it before proceeding to Step 7.
    c. <u>It may be TEC waste</u> (e.g., a small piece of paper), it needs to be inspected before being discarded. All smaller pieces of litter that may be TEC waste must be picked up and inspected, no matter how “sure” you are that it is not TEC waste. To inspect smaller pieces, such as torn bits of paper follow 11.b. above.

STEP 7: Record TEC Waste

Use this 4-part process to photograph, categorize, and count TEC waste in QuickCapture. The acronym HPPR was adopted to remember the steps to follow each time a piece of TEC waste was found: (1) **Hoop** it; (2) **Photograph**, categorize, and count it; (3) **Pick** it up and place in container, and (4) **Remove** hoop.

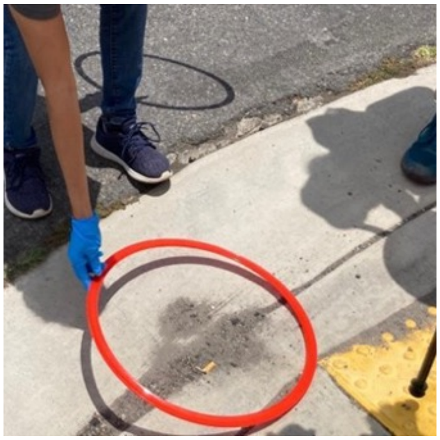
Fig. 27. “Hooping” a cigarette butt.

1. Place hoop around TEC waste (See Fig. 27)
    a. Place a hoop on the ground around the TEC waste. The hoop serves as a frame of reference to determine the size, shape, and location of the TEC waste.
    b. If the waste is in the gutter or in an unsafe area, use a grabber/tweezer to pick up and move it to the nearest safe spot (onto the curb or a similar safe spot).
    c. Alert team member who is recording data in QuickCapture that you have “hooped” some TEC waste.

   d. Once you have placed the hoop, do NOT leave the hooped product(s) until the waste has been recorded in QuickCapture and collected. This is to ensure that products are not miscounted.
2. Photograph, Categorize, and Count
   a. Categorize and count each type of TEC waste inside the hoop (See Appendix C for details):
      i. Cigarette butts - includes unsmoked portion of commercial cigarettes filtered or unfiltered, filters, paper covering of butts.
      ii. Other tobacco products - include all other products, smoked or smokeless, (i.e., cigars, cigar tips, cigarillos), advertising and packaging of all tobacco products.
      iii. E-cigarettes - includes all vape products (cannabis or nicotine), advertising, and packaging.
      iv. Cannabis - includes commercial cannabis blunts, edibles, advertising, and packaging.
      v. Multiple use - includes items that may be used for any smoked product, including hand rolled butts, rolling paper, lighters, torches, pipes.
      vi. Unsure - cannot determine definitively that the waste is NOT tobacco waste.
   b. Place the monopod as close to the side of the hoop as possible. The whole hoop should be in the image.
   c. Use QuickCapture to record the item.
      i. Select the type of TEC waste.
      ii. Take a picture of the waste item(s) inside the hoop. Check the picture to ensure the TEC waste is visible.
      iii. Enter the number of items in the photograph (Fig. 28).
   d. If there is more than one type of product inside the hoop, you will need to take one picture for each type and record the category and number of each type with its corresponding photograph.
      i. For each type of waste inside the hoop, repeat steps c-d for each type. For example, if there are 2 cigarette butts and 1 vape item inside the hoop, you would record Cigarette Butts Only, take the picture, enter the number 2. Then record E-cigarette, take the picture again, enter the number 1.
3. Pick-up the TEC waste. Use the grabbers/tweezers to pick up the litter and place it in the labeled bottle. See Fig. 29 and Fig. 30.
4. Remove hoop
   a. Pick up the hoop after placing the waste in the appropriate container.
   b. Remember to stay with the hoop from the time you have placed it on the ground until you place the TEC waste in its appropriate container. Prevent errors!

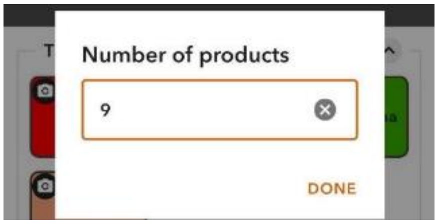

Fig. 28. After selecting the type of waste and taking the photo, enter the number items in the photo.

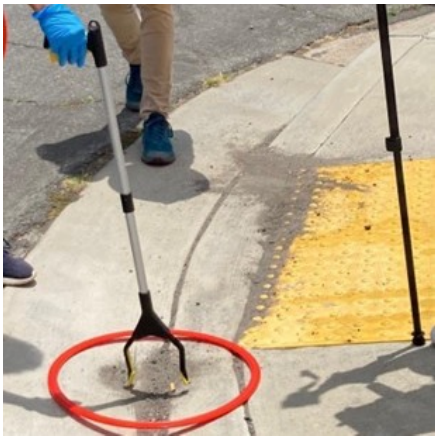
Fig. 29. Use a grabber to pick up TEC waste after it is recorded in QuickCapture.

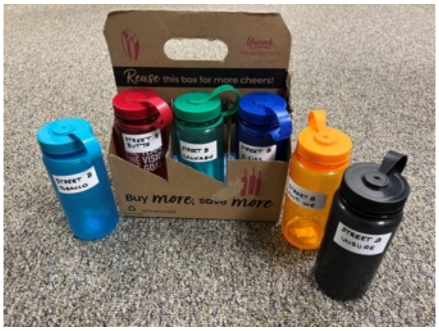
Fig. 30. After recording the type and number of waste items inside the hoop, place each piece of waste in the appropriate container by type.

STEP 8: Complete Assessment of Census Block

Use this step to complete the collection of TEC waste on each street on or inside the census block.

1. Repeat Steps 6 and 7 until you reach the end of Street A / the beginning of Street B.
   a. As you approach the end of Street A, check the census block's **AssessmentForm.doc** to ensure you do not go past the boundaries for Street A. Streets typically end at the diagonal from the inner corner to the outer corner as indicated on the map.
2. After completing Street A, make sure all collection containers used on Street A (Fig. 30) are closed securely. Switch out the set of containers for Street A for the set of empty containers for Street B.
3. Enter the BlockID, Street Letter, and Pass Number (1) for Street B.
4. Repeat until each street and alley has been completed. You should have a full set of collection containers for each street/alley on this census block. Make sure the lids are securely fastened! The TEC waste in these containers will be weighed in Step 12. It is very important that nothing is lost.

STEP 9: Quality Control

Use this step to collect data to estimate the percent of TEC waste missed by a team of trained research assistants using Steps 6-8.

1. Select a sample of streets for a second pass.
   a. 10% of census blocks should be selected for quality control.
   b. Within each census block, one street should be selected for a second pass.
2. The second pass should be done as soon as possible after the first pass is completed to avoid new TEC waste being deposited.
3. Enter the BlockID, Street Letter, and Pass Number (2); press done. [For second pass in Data Collection, the pass number is always 2.]
4. Switch out the set of containers with a set of empty containers for the second pass. Use a Sharpie to mark the labels on the containers "PASS TWO."
5. Repeat Steps 6-8 for the selected street.

STEP 10: Collect TEC Waste in Irregular Areas

Use this step to collect TEC waste in any irregular areas inside the block. <u>This step is only necessary if there are irregular areas inside the block.</u>

1. Walk to irregular area #1 (Consult **AssessmentForm.doc**.) If the irregular area is large, it should be divided into sections, with boundaries clearly established, and teams of project staff assigned to sections.
2. Switch out the set of containers with the set of empty containers for the irregular area (or sections). You will need one set of containers for each area (or section).
3. Use a Sharpie to clearly label containers should be labeled "Irregular Area."
4. Use caution when collecting TEC waste in irregular areas. Only assess if you can do so safely:
   a. In a recreational area, assess hiking paths and do not wander off.
   b. In parking lots, assess the perimeter including parking spots, pedestrian walkways within the parking lot, and any planters/dividers between parking stalls. Do not assess areas with moving traffic.
   c. In shopping plazas, assess the sidewalks and other accessible areas surrounding the storefronts (e.g., outdoor seating).
5. Within the boundaries of the irregular area or section, follow Step 6.
6. Place the TEC waste in the appropriate container for the irregular area or section and type of waste.
7. When Step 6 has been completed for all parts of the irregular area, count all TEC waste by type.
8. Open QuickCapture. Enter the block ID (100-160), street letter (A-[unknown]), and pass number (1). Enter and record total number of each type.
9. After recording total counts, discard the TEC waste collected in the irregular area. TEC waste collected in irregular areas is not weighed.

STEP 11: Prepare to Transport TEC Waste and Equipment

Use this step to prepare TEC waste and equipment for transport.

1. Reconvene at the initial meeting area.
2. In preparation for transport: remove all containers from the cardboard carriers and carefully store any containers with TEC waste in a 5 gallon plastic food storage bucket, and close with an airtight lid for later weighing. To clean equipment, soak with 75% isopropyl alcohol (spray or wipes) and dry with paper towels. Wear gloves for cleaning equipment! Put all trash into trash bags. If possible, find a public trash can at the data collection site to dispose of the collected trash and any TEC waste collected in irregular areas. If there is not a place to discard the trash at the site, place the trash bag in a drywall bucket with an airtight lid for transport.
3. Pack the cars and you are ready to return to the lab and begin Step 11.

STEP 12: Weigh TEC Waste

You will use Survey123 to record the weight of each type of TEC waste collected on each street.

1. Gather equipment:
    a. Kitchen scale that can measure from 0.1g to 600g with a readability of 0.01g, and a 500g calibration weight
    b. Containers of TEC waste (labeled with Street Letter, Pass Number, Waste Type)
    c. Large tweezers from rolling cart (See Step 2)
    d. Cleaning supplies (See Step 2)
    e. iPad with Survey123
2. Open Survey123. Starting with Street A, record the BlockID, Street Letter, Pass Number
3. Following manufacturer's instructions, calibrate the scale.
4. Record the calibrated weight in Survey123.
5. Weigh one container of TEC waste found on Street A (i.e., container with content).
    a. Record the weight by type in Survey123. (See Fig. 31.)
    b. Discard the waste in the trash.
    c. Weigh the empty container and record the weight by type in Survey123.
    d. Repeat 5.a. through 5.b. until each container of waste has been weighed and recorded (i.e., the container with TEC waste and the empty container).
6. Begin Street B. Record the BlockID, Street Letter, Pass Number
7. Repeat #5-#6 for each street.
8. Clean empty containers thoroughly with 70% isopropyl alcohol and alcohol wipes/paper towels.

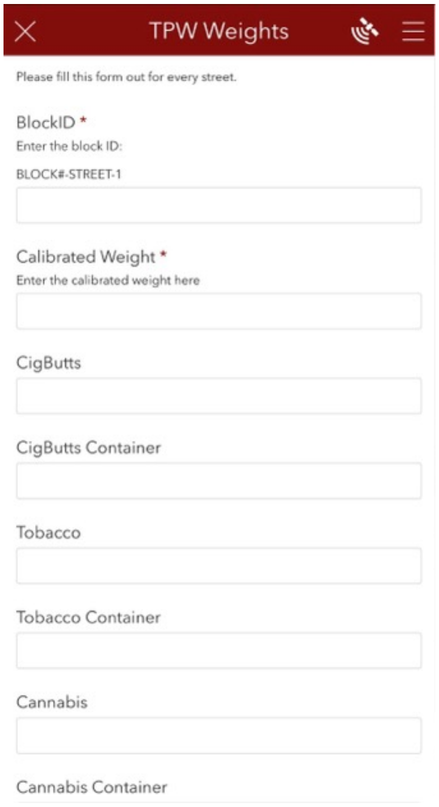

Fig. 31. Screenshot of Survey123 used to record weight of TECs waste by type

STEP 13: Complete Field Data Collection

Use this step to complete Field Data Collection.

1. Record date(s) data collection was started and completed on each street in **DataCollectionPlan.xls.** Typically, streets were completed in one day, however occasionally data collection required more than one day.

Part 4: Archival Data Collection

This is the fourth of six parts of the study protocol. The goals of Archival Data Collection are to:

- determine and record the surface area of the regular and irregular areas assessed on the census block;
- determine and record the amount of rainfall in the 30 days prior to data collection;
- determine and record the # of days between street sweeping and data collection on each street in the census block.

You will need access to ArcGIS, **AssessmentForm.doc** for each census block, and the **DataCollectionPlan.xls** to complete Archival Data Collection.

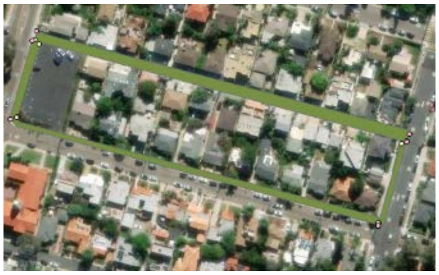

Fig. 32. Census block with polygons using two coordinates at the end of each street.

STEP 1: Determine Surface Area of Regular and Irregular Areas

Use this step to determine the surface area of the regular and irregular areas where data were collected.

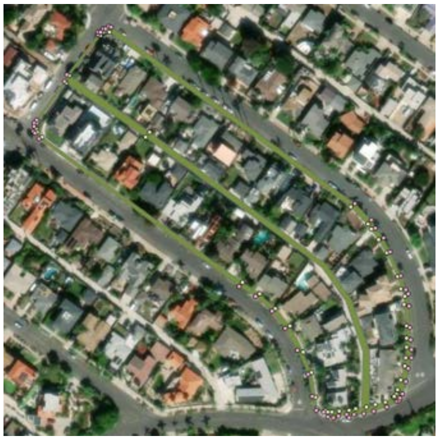

Fig. 33. Polygons on an irregularly shaped street with multiple coordinates.

1. Open ArcGIS Online, select your project, and select a census block and
2. Beginning with Street A, compare the placement of coordinates recorded on the map on the census block's **AssessmentForm.doc** with the coordinates on ArcGIS. Move the coordinate points in ArcGIS to the appropriate location if they are misplaced.
    a. After all the coordinate points have been correctly placed, use the coordinates to draw a polygon to account for Street A's surface area. See Fig. 32.
    b. If Street A does not follow a general shape of a rectangle, make sure to include the indentations necessary to follow the outline of the street. See Fig. 33.
3. Repeat #2 until polygons have been drawn for each street in the census block.
4. Beginning with Irregular Area #1, compare the placement of the perimeter recorded on the map on the census block's **AssessmentForm.doc** with the perimeter on ArcGIS. Adjust the perimeter as necessary. See Fig. 34.
5. Repeat #4 until all perimeters have been verified.
6. In ArcGIS, calculate the surface area of each polygon (i.e., each street and each irregular area will have a polygon). (See Fig. 35 and Fig. 36)
7. Record the surface area of each polygon into **DataCollectionPlan.xls**

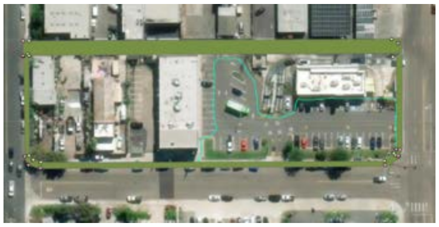

Fig. 34. Census block with irregular area perimeter marked in teal.

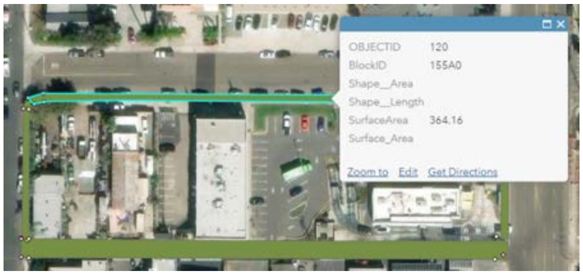

Fig. 35. Surface area of Regular Area calculated in ArcGIS.

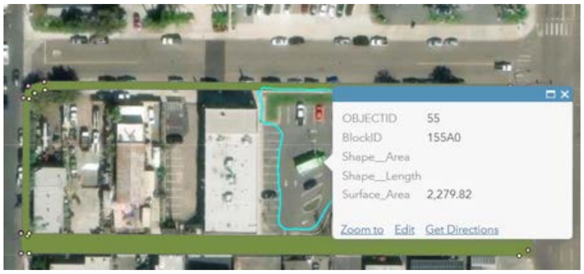

Fig. 36. Surface area of an Irregular Area calculated in ArcGIS.

STEP 2: Calculate Rainfall 30 Days Prior to Data Collection

Use this step to calculate the amount of precipitation in the census block during the 30 days prior to data collection. You will need access to Weather Underground (https://www.wunderground.com/) to complete this step.

1. Open **DataCollectionPlan.xls** and select a census block.
2. Open ArcGIS Online and locate the centroid of the census block (the latitude and longitude for each census block are recorded in the **DataCollectionPlan.xls**.
3. Locate the Weather Underground weather station that is closest to the centroid of the block. Google Maps may be helpful.
4. Confirm that the closest weather station has tracked precipitation for at least 1 month prior to the first date of data collection on that census block. See https://www.wunderground.com/ for details on precipitation tracking. If the closest weather station has not tracked precipitation for at least 1 month prior to the first date of data collection on that census block, select the next closest weather station. Repeat as necessary until you have found a weather station with precipitation for at least 1 month.
5. Using ArcGIS, measure the distance between the selected weather station and the census block centroid.
6. Beginning with Street A, use the **DataCollectionPlan.xls** to determine the first date of data collection on Street A. (If data collection on Street A took more than 1 day, use the first date).
7. Record the following data for Street A relative to the first date of data collection on that street:
   a. Date of most recent rainfall. Rainfall = precipitation amount greater than 0.00.
   b. Amount and date of rainfall recorded each day on the 30 days prior to data collection (do not count the day of data collection).
8. Use data collected in #6 to determine:
   a. Number of dry days (i.e., days with no precipitation recorded) between the day prior to data collection and the last rainfall.
   b. Date and amount of maximum precipitation on any one day in the 7 days prior to data collection.
   c. Date and amount of maximum precipitation on any one day in the 30 days prior to data collection.
   d. Total amount of precipitation recorded in the 7 days prior to data collection.
   e. Average precipitation in the 7 days prior to data collection.
   f. Total amount of precipitation recorded in the 30 days prior to data collection.
   g. Average precipitation in the 30 days prior to data collection.
9. Repeat # 6, 7, and 8 for each street on or within the census block.

STEP 3: Determine Date of Most Recent Street Sweeping

Use this step to calculate the number of days between the date of data collection and the date of the most recent street sweeping. You will need access to a calendar to complete this step.

1. Open **DataCollectionPlan.xls** and select a census block.
2. Beginning with Street A, use the **DataCollectionPlan.xls** to determine the date of data collection on Street A. If there are multiple dates (i.e., data collection on Street A took more than 1 day, use the final date).
3. Using the street sweeping schedule recorded in the **DataCollectionPlan.xls**, consult a calendar to determine the date of street sweeping prior to data collection. For example: If data were collected on 4/27/23 and the street sweeping schedule was the 4th Friday of even months, the date of street sweeping prior to data collection would have been in the previous even month (i.e., February) on the 4th Friday of that month, or 2/24/23.
4. Add street sweeping date for Street A to the **DataCollectionPlan.xls**
5. Repeat # 1-4 for each street on the census block.
6. Calculate the number of days between the street sweeping date and the data collection date for each street. Record in the **DataCollectionPlan.xls**.

Part 5: Data Management

The goal of Data Management is:

- upload and clean all data collected during Pre-Assessment, Active Assessment, and Data Collection

You will need access to ArcGIS, **AssessmentForm.doc** for each census block, and the **DataCollectionPlan.xls** to complete Data Management.

STEP 1: Upload and Clean Data

Use this step to ensure data have been uploaded correctly, any corrections noted on the block's **AssessmentForm.doc** have been addressed., and(3) that the data reflect what has been collected.

1. Following manufacturer's instructions, upload the data from QuickCapture and Survey123 on every device used to collect data.
2. Select a census block and open the census block's **AssessmentForm.doc** to check for any notes. These notes may include (but are not limited to) data that need to be edited (e.g., delete a data point, change a miscoded product to the correct product, add a Point of Interest) or provide information about any deviation encountered during data collection such as construction or other obstacles. Use the editing tool in ArcGIS to correct
    a. TEC waste items that were incorrectly categorized or counted,
    b. Inaccurate codes used for Point of Interest, and
    c. Incorrect location of data points. (If the GPS receiver experiences interference, data points may appear outside of the assessment area. These data points need to be relocated in ArcGIS inside the boundaries of the assessment area.)
3. Review data for potential errors related to network/uploading issues, including (but not limited to) duplicated data.
4. Extract the data from ArcGIS for analysis and review.

Part 6: Data Reporting

The goals of Data Reporting are:

- describe of census block characteristics;
- count TEC waste by type;
- calculate the density of TEC waste by type per meter squared (m^2).

STEP 1: Describe Census Block Characteristics

Use this step to ensure that data will be comparable across multiple projects and locations.

1. Include the surface area of the block and demographic and socioeconomic characteristics of residents in your report. See Table 1 for a list of characteristics to include.
2. As standard practice in reporting, always report mean, standard deviation, minimum, 1st quartile, median, 3rd quartile, and maximum.

Table 1. Census block characteristics.

Characteristics of Census Blocks and their Residents	Mean	SD	Min	Q1	Median	Q3	Max
Block Area (m^2)[a]							
Mean Population[a]							
Population Density (persons/km^2) [a]							
Median Age (years) [a]							
Female (%)[a]							
Race (%)[a]<br>White (non-Hispanic)<br>Black(non-Hispanic)<br>Asian<br>Hispanic							
Foreign Born (%)[b]							
Educational Attainment (% college) [b]							
Median Income ($)[b]							
Median Rent ($)[b]							
Median Home Value ($)[b]							
Poverty (%)[b]							
Public Assistance (%)[b]							
Unemployment (%)[b]							
Smoking Prevalence (%)[c]							
Raw Walkability Index[d]							
Normalized Walkability Index (scale of 0-1) [d]							

Notes. [a] Block-level data from U.S. Census [b] Tract-level data from American Community Survey. [c] tract-level data from the Behavioral Risk Factor Surveillance System. [d] EPA National Walkability Index.

STEP 2: Describe Survey Characteristics

Use this step to describe the characteristics of the area surveyed.

1. Survey characteristics include surface area and days since street sweeping. If more than one round of data collection occurred, also report the number of days between rounds. (See Table 2.)
2. Report the mean, standard deviation, minimum, 1st quartile, median, 3rd quartile, and maximum.

Table 2. Survey characteristics reported for two rounds of data collection.

Characteristics of TEC Waste Surveys	Min	Q1	Mdn	Q3	Max	Mean 95% CI	GMean 95% CI
Area Surveyed (m^2)[e]<br>Regular Area<br>Irregular Area<br>Total							
Days Between Collections Round 1 and Round 2[e]							
Days Since Last Street Sweeping[f]<br>Round 1<br>Round 2							
Days Since Last Rain Event[g]<br>Round 1<br>Round 2							

Legend: Min: minimum; Q1: first quartile; Mdn: Median; Q3: third quartile; Mean: arithmetic mean; GMean: geometric mean; CI confidence interval.
[e] GIS data collected for N=60 census blocks. [f] Street sweeping data obtained from local city governments. [g] Rainfall data obtained from the closest weather station.

STEP 3: Determine Count and Density of TEC Waste

Use this step to determine the number and density of TEC waste by type in the area surveyed.

1. Calculate the total count of each type of TEC waste located in the regular and irregular areas of each census block. (See Table 3.)
2. Report arithmetic and geometric means, their 95% confidence intervals, minimum, 1st quartile, median, 3rd quartile, and maximum.
3. Calculate the density of TEC waste per meter squared to compare TEC waste in different locations or during different timeframes.
4. Report density per 100 meter squared. Report arithmetic and geometric means, their 95% confidence intervals, minimum, 1st quartile, median, 3rd quartile, and maximum. (Use either the arithmetic or geometric mean.)

Table 3. Descriptive statistics and mean estimates of TEC waste counts per block and density per 100 m^2 by type of waste.

Type of TEC Waste	Min	Q1	Mdn	Q3	Max	Mean 95% CI	GMean 95% CI
Count per Block							
Cigarette Butts							
Other Tobacco Product							
Cannabis Product							
E-cigarette							
Dual-Use							
Unsure							
All TEC Waste							
Density (Count per $100m^2$)							
Cigarette Butts							
Other Tobacco Product							
Cannabis Product							
E-cigarette							
Dual-Use							
Unsure							
All TEC Waste							

Legend: Min: minimum; Q1: first quartile; Mdn: Median; Q3: third quartile; Mean: arithmetic mean; GMean: geometric mean; CI confidence interval

Appendix A: Acronyms and Terms

Acronym or Term	Definition
GIS	Geographic Information System
GPS	Global Positioning System
TEC	Tobacco, E-cigarette, and Cannabis
TPW	Tobacco Product Waste
Across the Block	Area directly across the street from the perimeter of the census block.
BadElf Surveyor	The GPS surveyor used to improve precision of geocoding of data. Link: https://bad-elf.com/products/be-gps-3300
BlockID	A three-digit block number that is assigned to each census block.
Census block	The smallest geographic area for which the Bureau of the Census collects decennial census data.
Census Block Perimeter	The outside edge of the census block is the point where the outside edge of the gutter meets the surface of the street, approximately 18" from the curb.
Curb	The stone or concrete edge of the curb strip or sidewalk.
Curb strip	The unpaved area between the outside edge of the sidewalk and curb.
Functional sidewalk	Paved or unpaved area adjacent to sidewalk, gutter, or curb that is regularly used by pedestrians.
Gutter	The concrete or stone area between the edge of the curb and the street surface, typically concrete and designed to carry water during rain events.
Inside edge of sidewalk	The inner edge of the sidewalk, bordering on private property.
Irregular area	Other publicly accessible areas within the census block, such as parking lots, hiking trails, or shopping plazas.
Lawnmower[1]	Walk in a continuous s-shaped pattern, as if you were mowing a lawn, observing the area directly in front of you. Typically one person walks from one end of the assessment area to the other, makes a close turn and walks back parallell to the first pass, repeating until the entire area is covered.
My Maps	Google Maps tool to create custom maps. https://www.google.com/maps/about/mymaps/
On the Block	Any area inside the perimeter of the census block.
Outside edge of sidewalk	The outer edge of the sidewalk, bordering the curb strip or, if there is no curb strip, the curb.
Pass	The number used to characterize the type and timing of the data being collected.

Points of Interest	Features that describe the census block, including land use, tobacco-related behaviors, and city services.
QuickCapture	App used to capture data in ArcGIS.
Regular area	Public sidewalks on the perimeter of or within the census block. The regular area extends from the inside edge of the sidewalk to the outside edge of the gutter, typically about 18” from the curb, including the curb strip and gutter.
Snowplow[1]	Walk in a straight line from one end of the assessment area to the other, observing an area directly in front of you approximately shoulder-width. Typically used in a wide area with several observers walking next to each other in a row, each person responsible for the area directly in front of them.
Street	The letter (e.g., A, B, C) assigned to each street, beginning with the northern most street and proceeding in a clockwise direction.
Survey123	App used to capture data in ArcGIS.

Appendix B: Points of Interest

LAND USE CATEGORIES	SUB-CATEGORIES	IMAGE
**Single Family Homes**<br>Residential buildings that do not share wall or walls with other residential buildings.	None	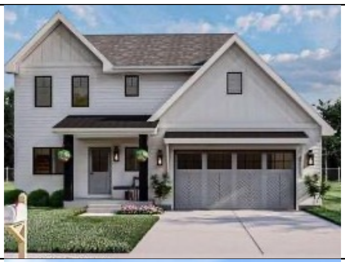
**Multiunit Housing**<br>Residential buildings with more than one unit that shares one or more walls with other units.	None	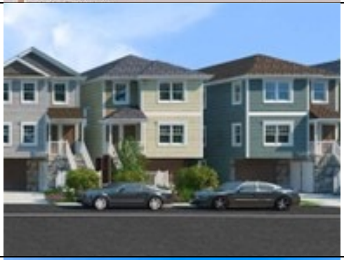
**Parks/Recreation**<br>Area set aside for recreation and/or for the protection of wildlife. Includes both natural and built environments.	None	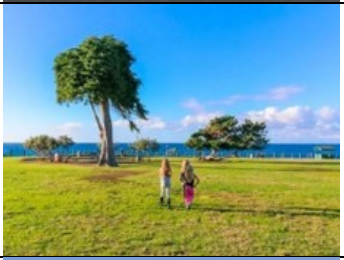
**Parking Lot**<br>Indoor or outdoor area designated for parking vehicles temporarily. May be public or private.	None	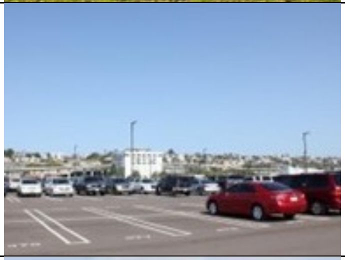
**Manufacturing**<br>Business devoted to design or production of products that will be sold.	None	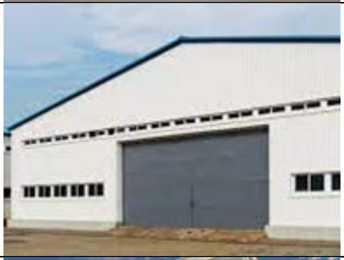
**Mixed Use**<br>A single building with multiple uses such as commercial, cultural, entertainment.	None	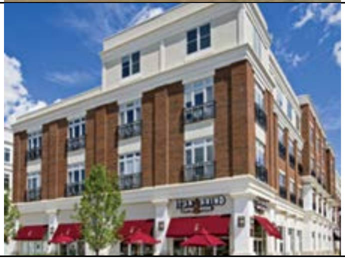

**Health Care**<br>Building designed to provide organized health care to individuals or a community.	**Clinic**<br>Offers a range of health care services (e.g., primary care, specialty care, radiology, laboratory, behavioral health)	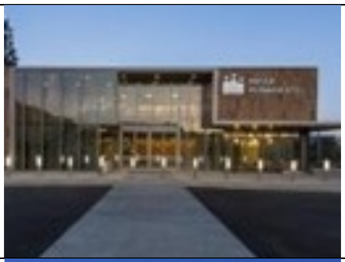
	**Provider Offices**<br>Individual or group professional practice.	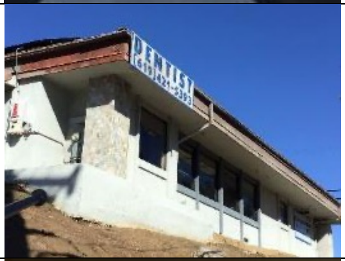
	**Behavioral Health**<br>Mental health services including substance use treatment.	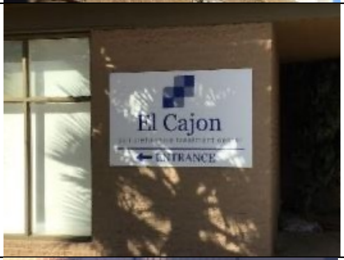
	**Other**<br>Health services such as physical therapy, dialysis, laboratory, nutrition counseling.	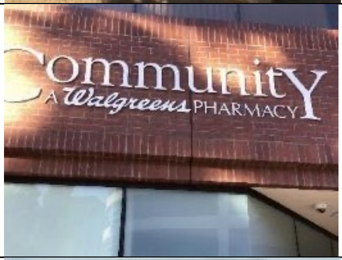
**Services**<br>Business offering professional services for personal, professional, or household needs.	**Personal**<br>Services offered for individual care such as hair salon or nail salon.	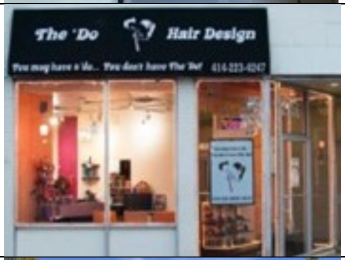
	**Professional**<br>Specialized services offered for business or personal needs, such as banking, legal services, tax preparation.	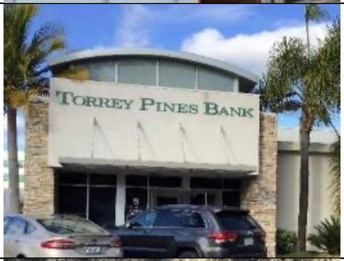
	**Trades**<br>Specialized services offered for vehicle or home repair/maintenance such as car repair, plumber, rug cleaning.	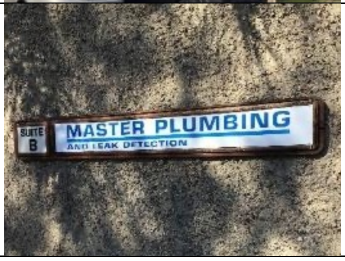

	**Other**	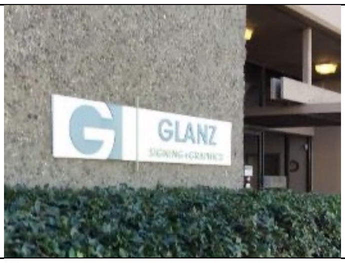
**Retail**<br>Business selling merchandise, including clothing, household goods, and groceries.	Tobacco/E-cig<br>Products for sale include commercial tobacco and/or vapes.	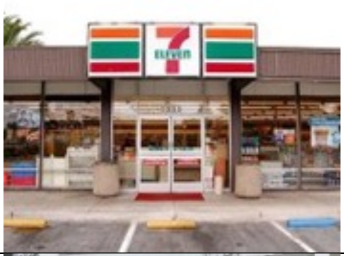
	Cannabis<br>Products for sale include cannabis.	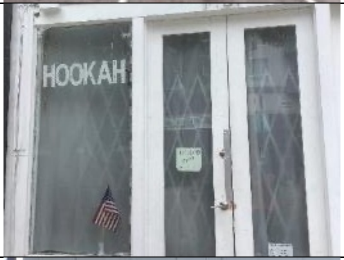
	Alcohol<br>Products for sale include alcohol.	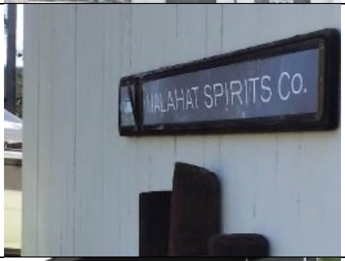
	None<br>Products for sale do NOT include commercial tobacco, vapes, cannabis, or alcohol.	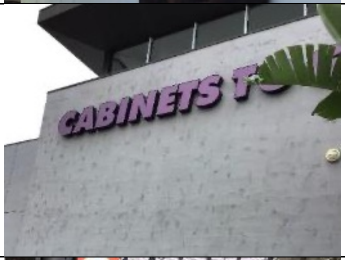
**Education**<br>Institution to educate the public, including training and certificate programs.	**Pre-school**	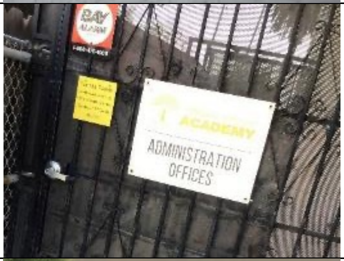
	**Elementary school**	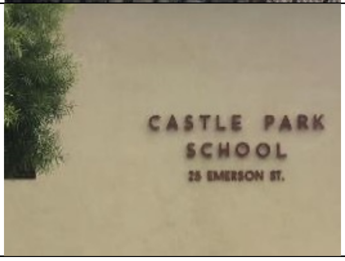

	**Middle school**	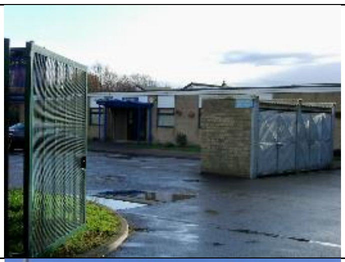
	**High School**	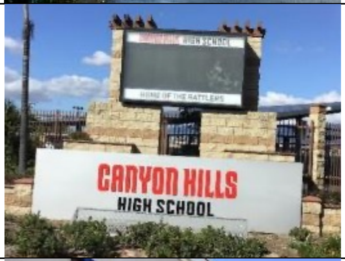
	**Post-High School**<br>Includes community colleges, universities, trade schools, and training programs.	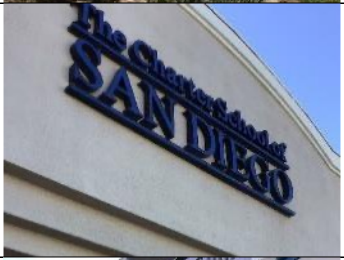
**Hospitality**<br>Business that offers entertainment, food, and/or housing. NOTE: Within each subcategory, record whether or not alcohol is served.	**Hotel**<br>Any business that allows rental of rooms for a temporary stay.	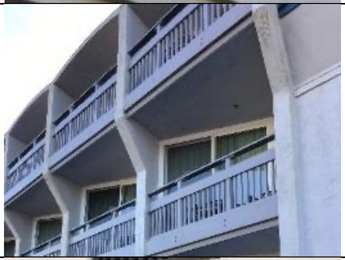
	**Bar/Restaurant**<br>A restaurant is a business that serves prepared food for in-person or take out with or without alcohol. A bar is a business that serves alcohol.	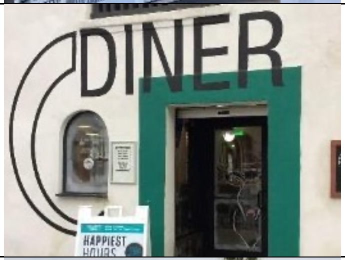
	**Coffee Shop**<br>A restaurant that primarily serves coffee and other drinks. May also serve food and/or alcohol.	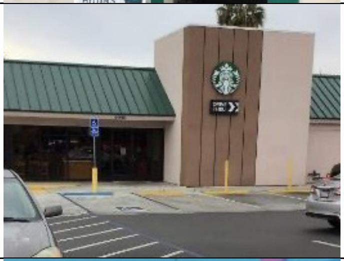
	**Entertainment**<br>A business that offers activities such as movie theater, concert venue, comedy club, or gym.	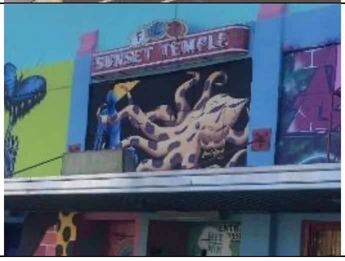

**Other**<br>Vacant lots, construction, and other types of land use that do not fit into existing categories.	None	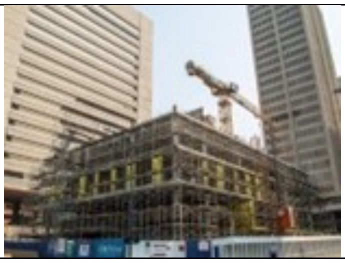
**TOBACCO-RELATED BEHAVIORS**	**SUB-CATEGORIES**	**IMAGE**
**Active Smoking**<br>Individual smoking or vaping any product.	None	
**Butt Receptacle**<br>Receptacle specifically for disposal of smoked products. May be included as part of a public trash can.	None	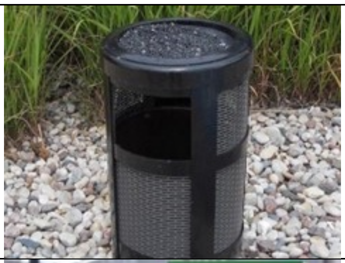
**Smoking Area Signage**<br>Designated area where smoking or vaping is allowed.	None	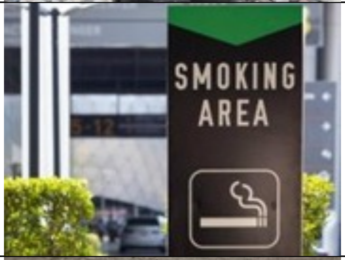
**No Smoking Signage**<br>Designated area where smoking or vaping is not allowed.	None	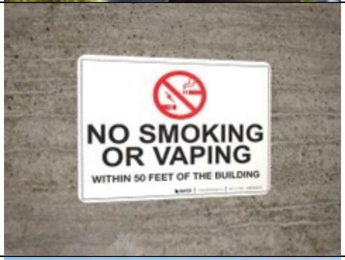
**Advertising**<br>Signs advertising any type of tobacco product, including vapes and cannabis, regardless of their location (e.g., storefront, billboards, stickers on bus stops)	None	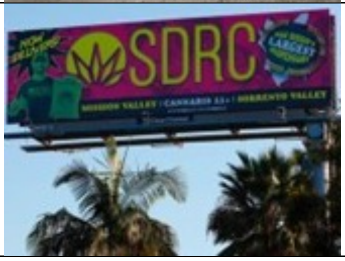
**CITY SERVICES**	**SUB-CATEGORIES**	**IMAGE**
**Bus Stop**<br>Marked by MTS sign, with or without a shelter or bench.	None	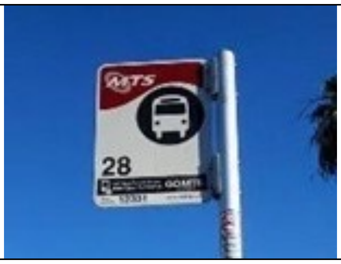

**Public Trash Cans**<br>Trash cans or dumpsters located on public sidewalks or alleys. Do not include household trash cans (even if people sometimes use them as if they were public).	None	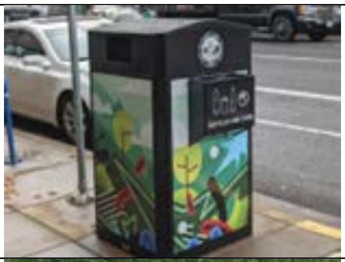
**Storm Drains**<br>Curbside grated areas along public sidewalks designed to capture excess water. Also include smaller grated areas on edges of parking lots or alleys.	None	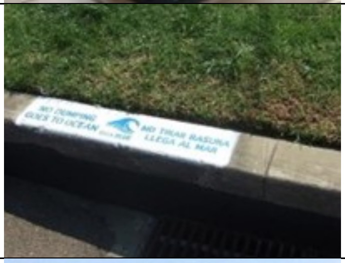
**Sensitive Habitat Signage**<br>Designated protected area within Coastal Zone of CA, described in the CA Coastal Act.	None	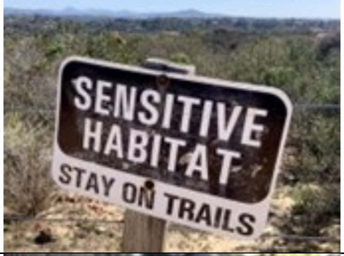
**Encampment**<br>Temporary shelter (e.g., tent, car, shopping cart) set up along street or sidewalk.	None	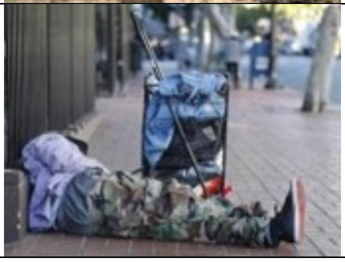

Appendix C: TEC Waste Identification Guide

Commercial Tobacco, Electronic Cigarettes, and Cannabis Waste Identification Guide

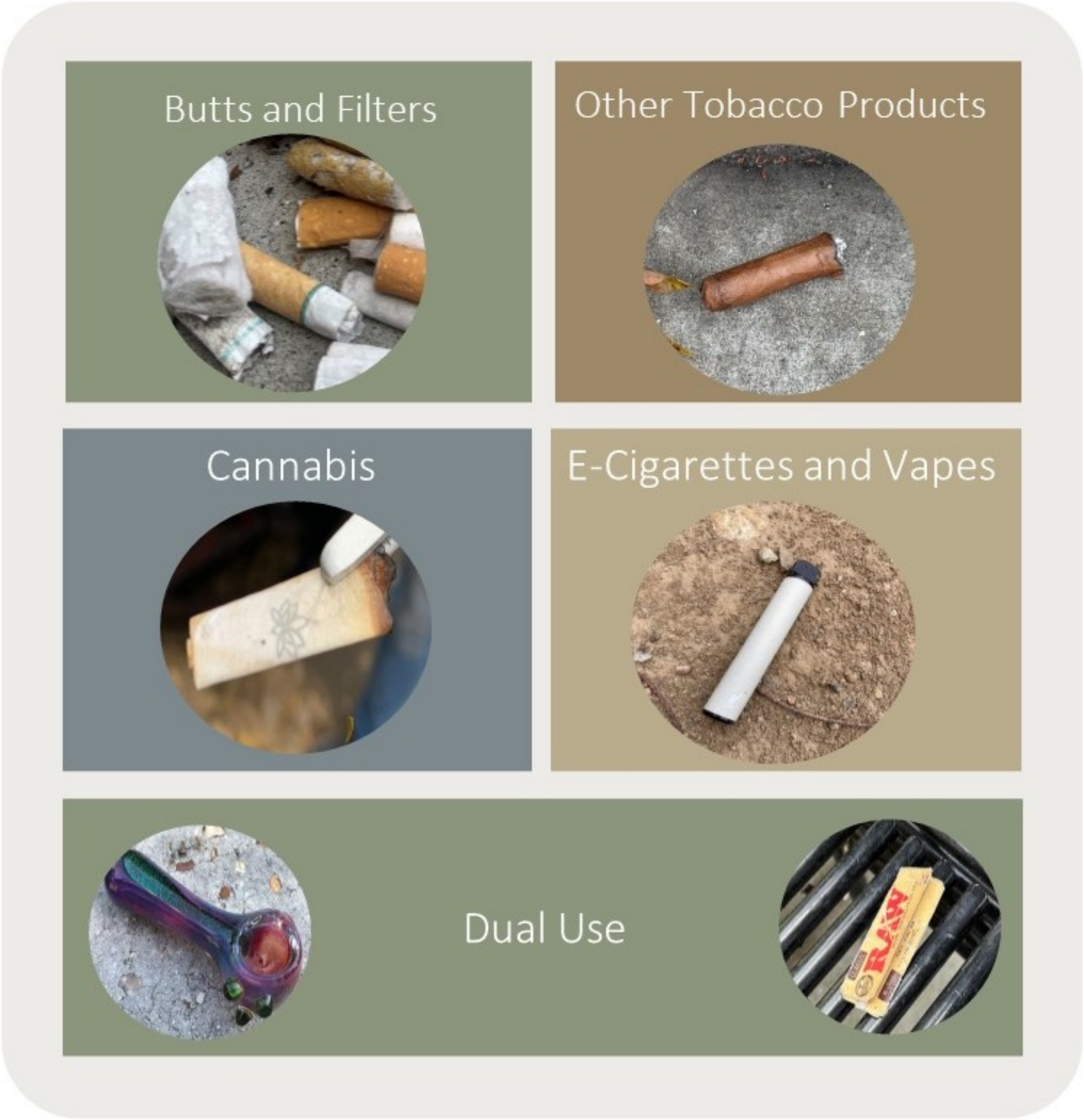

Commercial Butts and Filters

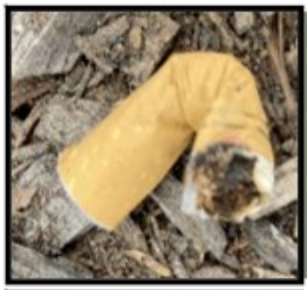

Filtered Cigarette Butt

Filter visible on one end, and tobacco on the other. Wrapped in paper imprinted with commercial tobacco branding

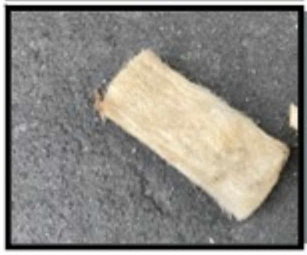

Intact Filter

Cellulose acetate, a type of plastic, filter with compact fibers. The paper covering has degraded

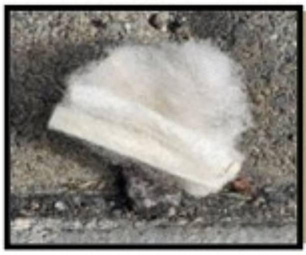

Degraded Filter:

Filter starting to degrade. Plastic fibers become loose or separated over time and look like cotton fluff.

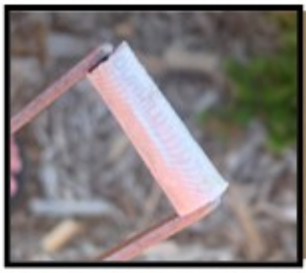

Unfiltered Cigarette Butt:

Tobacco visible on both ends. Wrapped in paper imprinted with commercial tobacco branding

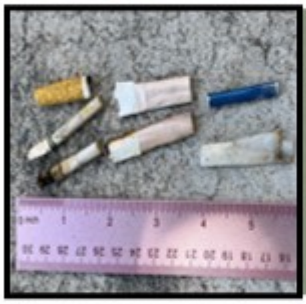

"Unique" Cigarette Butts:

Cigarette butts come in many different colors and sizes.

Other Tobacco Products

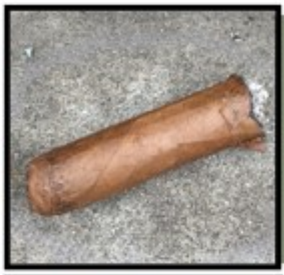

Cigars

Tobacco that is fermented and wrapped in a dried tobacco leaf or a material containing tobacco.

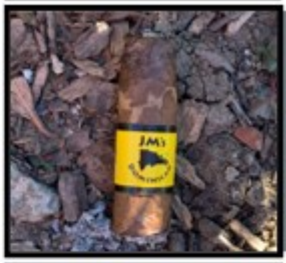

Cigar Bands

Paper or foil bands imprinted with commercial tobacco branding wrapped around one end of the cigar.

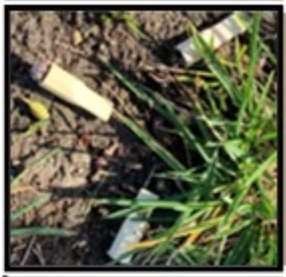

Plastic Cigar Tips

Plastic coverings or caps attached to the end of a cigar. Tips can sometimes retain traces of unsmoked tobacco.

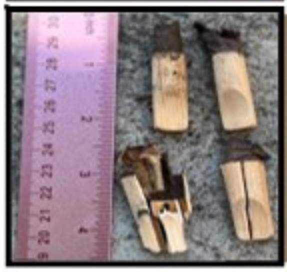

Wooden Cigar Tips

Wooden coverings or caps attached to the end of a cigar. Tips can sometimes retain traces of unsmoked tobacco.

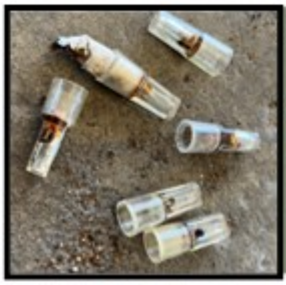

Other Holders

Plastic coverings or caps that can be used with cigarettes. Tips can sometimes retain traces of unsmoked tobacco.

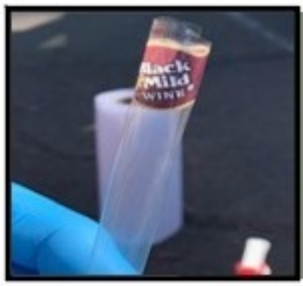

Product Packaging: Cigar Cellophane Wrappers

A transparent, thin sheet of cotton cellulose imprinted with commercial tobacco branding. Often long and slim in shape.

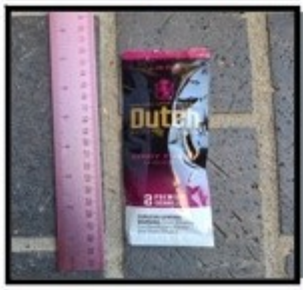

Product Packaging: Cigarillo Pouch

Compact and flexible plastic or foil bags used to package cigarillos. May have a re-sealable top. Imprinted with commercial tobacco branding.

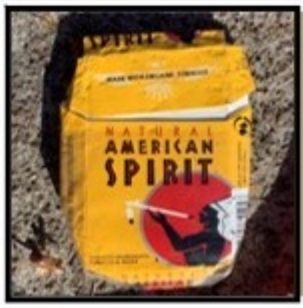

Product Packaging: Cigarette Pack

A rectangular paper or cardboard container imprinted with commercial tobacco branding used to hold cigarettes. It is often covered with plastic wrapping and has a foil lining.

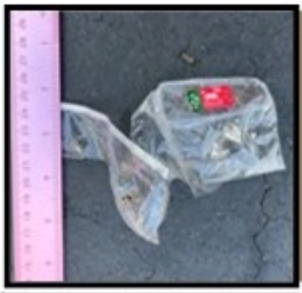

Product Packaging: Cigarette Pack Cellophane

The plastic covering of the cigarette packs. Typically has a gold or silver foil tear strip to aid in opening the pack. In CA carries the California tax label (shown on image).

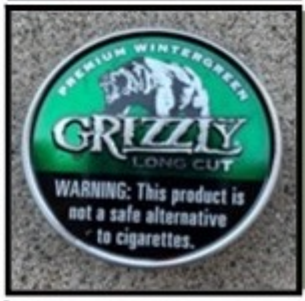

Product Packaging: Snus packaging

Small metal or plastic container with product branding. The containers often have a hinged lid, allowing easy access to the portioned snus inside.

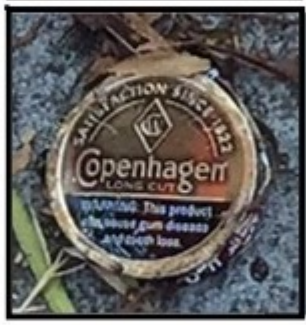

Product Packaging: Chewing or Spit Tobacco

Round or rectangular metal cans with product branding designed to hold cured tobacco in the form of loose leaf, plug or twist.

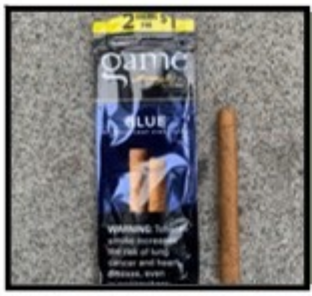

Cigarillos

Tobacco wrapped in a dried tobacco leaf or material containing tobacco. Smaller than regular cigars but typically larger than cigarettes. Often sold in packs of 2.

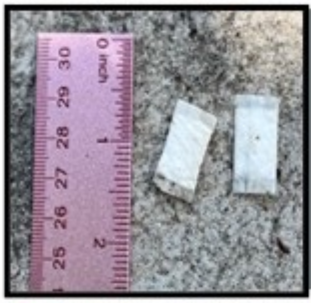

Smokeless Tobacco Product: Nicotine pouch

A small pouch that contains nicotine designed to be placed between the gum and lip. It is typically white in color.

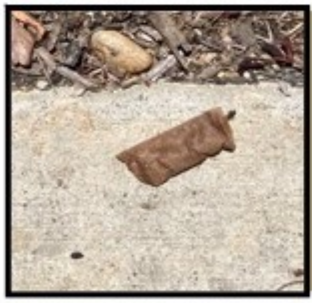

Smokeless Tobacco Product: Snus

A small pouch that contains processed tobacco designed to be placed between the gum and lip. It is typically a dark brown color, resembling a small tea bag.

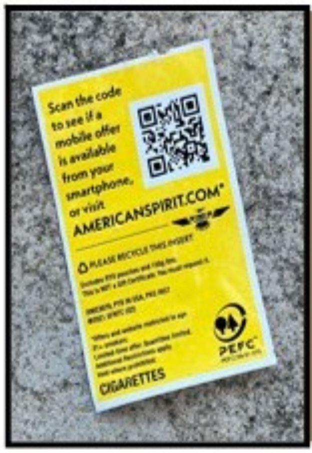

Tobacco Advertising

Any materials aimed at promoting tobacco use. Common materials include product discounts, frequent buyer cards, store openings.

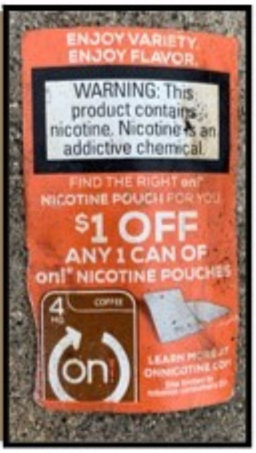

Cannabis

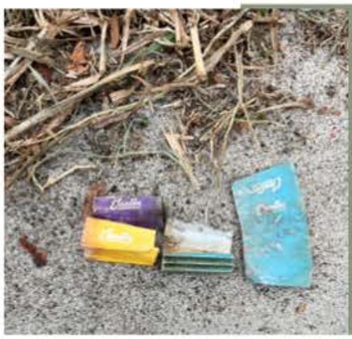

Pre-Rolled Cannabis "Butts"

Folded paper cylinder with commercial product branding. When purchased, a transparent tube filled with cannabis is attached to the paper cylinder.

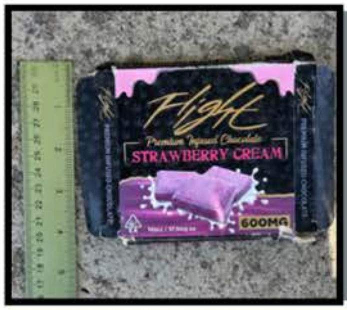

Cannabis Edibles

Edible items infused with cannabis. Cannabis-infused foods include baked goods, candy, chocolate, or potato chips. Can bear a resemblance to existing food items.

Cannabis Packaging

Packaging includes glass jars/bottles, resealable containers made of tin or plastic, plastic vials, tubes, wrappers, tin containers, mylar bags, or cardboard boxes with product branding and "cannabis", "marijuana", or "THC" on the label. In California, labels include a triangle with a marijuana plant and an exclamation mark.

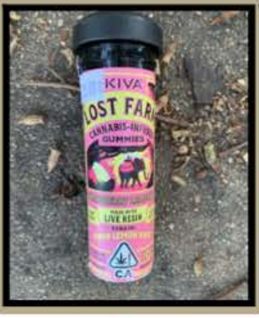

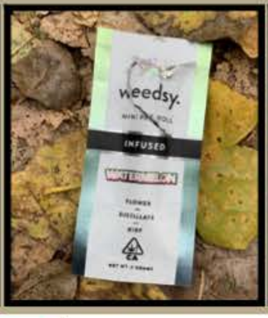

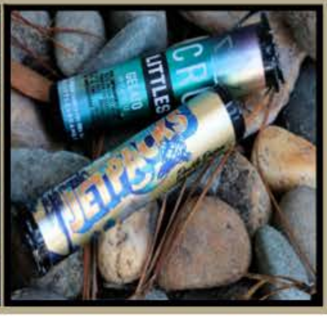

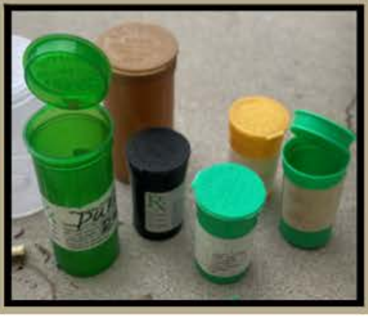

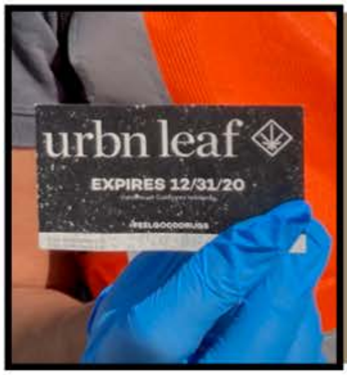

Cannabis Advertising:

Any materials aimed at promoting cannabis use. Common materials include product discounts, frequent buyer cards, store openings.

E-Cigarettes & Vapes

E-Cigarettes & Vapes

E-cigarettes (vapes) are devices that includes a battery, a cartridge or tank that holds nicotine or other substance, a heating element to heat the liquid and turn it into an aerosol of tiny particles (sometimes called a "vapor"), a mouthpiece or opening used to inhale the aerosol, and a rubber cap. Not all vapes contain nicotine; they may contain cannabis and other products.

E-cigarettes come in a wide variety of shapes, colors, and designs.

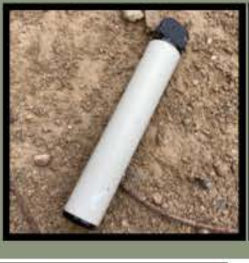
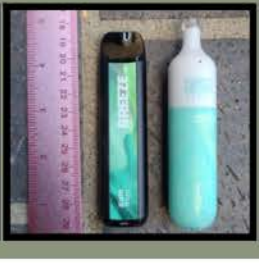
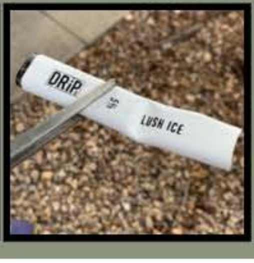

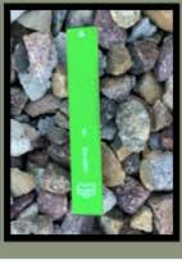
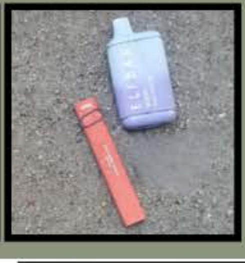

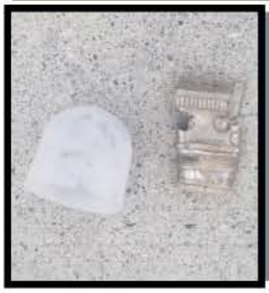

E-cigarette & Vape Caps

Protective plastic cover that fits over the mouthpiece or the top part of the device.

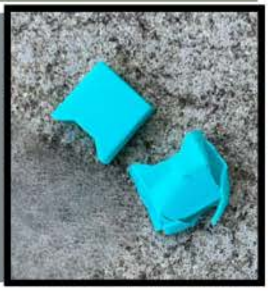

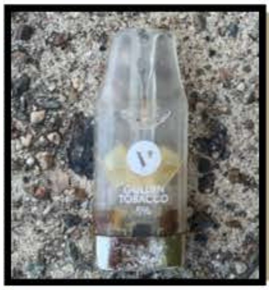

E-cigarette & Vape Mouthpiece

Cylindrical and tapered plastic or metal end of the device that resembles a small tube or funnel. The user places this in their mouth to inhale vapor.

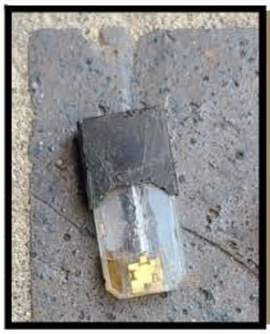

E-cigarette & Vape Pods

Pre-filled or refillable capsule-shaped cartridge that contains liquid with a metal rod (heating element) in the middle. Cannabis pods contain cannabis extracts or oils, while nicotine pods use e-liquid containing nicotine and other additives.

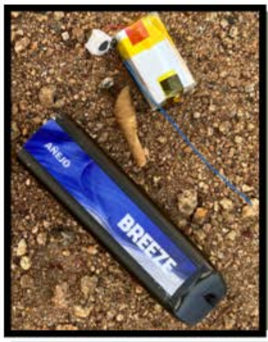

E-Cigarette & Vape Batteries

Batteries come in various sizes and shapes, ranging from cylindrical types to larger box-shaped designs. May be recharged and/or replaced. Located at the end of the device opposite of the mouthpiece.

E-cigarette & Vape Packaging

Cardboard or plastic container for the device and/or accompanying accessories (for example, refillable pods). Often features eye-catching graphics, branding, and product information.

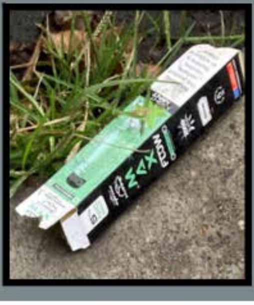

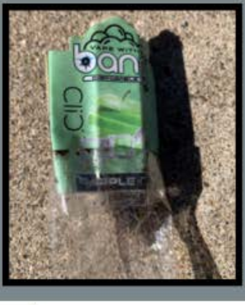

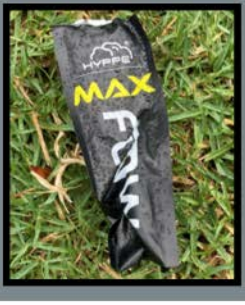

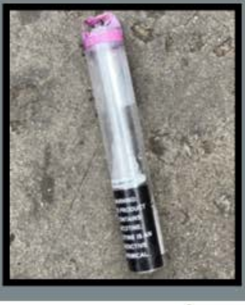

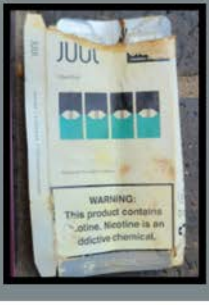

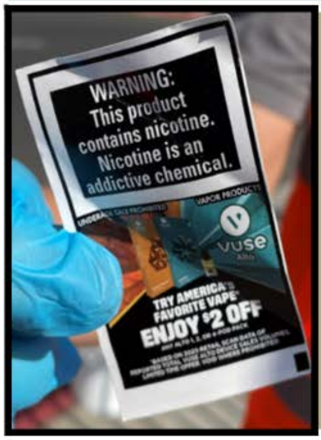

E-cigarette & Vape Advertising

Any materials aimed at promoting e-cigarette and vape use. Common materials include product discounts, frequent buyer cards, store openings.

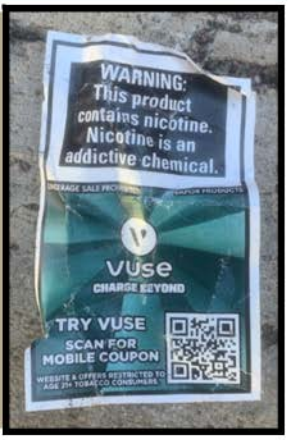

Dual Use

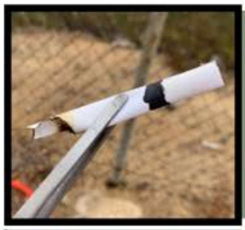

Hand Rolled Butts

Commercial rolling papers or other (i.e. store receipts) used to smoke tobacco or cannabis

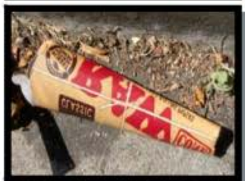

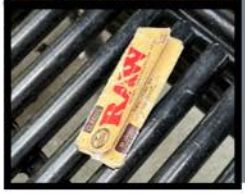

Rolling Paper

Thin, rectangular sheets made of various materials like rice, hemp, or wood pulp. They are usually translucent and are designed for rolling tobacco or cannabis into cigarettes or joints. Packaging for rolling papers is made of cardboard or plastic, depending on the brand.

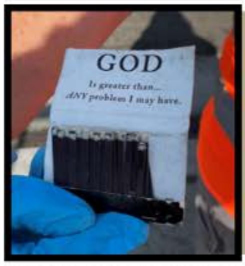

Matches

Typically comes in small, rectangular cardboard or paperboard boxes. The box may contain multiple paper or wooden matchsticks with red or white phosphorus tips.

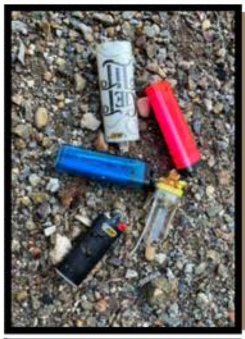

Lighters:

Come in various shapes, sizes, and materials. Typically made of plastic or metal and often have a rectangular or cylindrical shape.

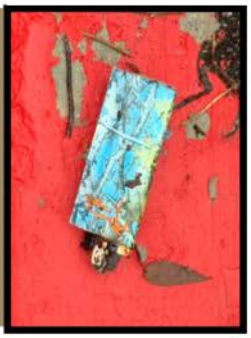

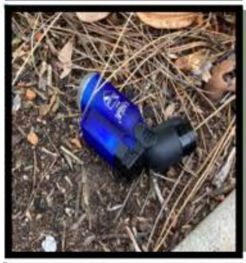

Torches:

A small, portable device that can generate a flame for ignition. Typically does not have commercial tobacco or cannabis branding.

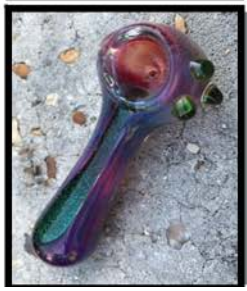

Pipes:

Commonly used for consuming tobacco and cannabis. It typically consists of a bowl, where the tobacco or cannabis is placed, and a stem with a mouthpiece. Can be made of glass, metal, or ceramic and comes in different shapes, sizes and colours.

Appendix D: Volunteer Training

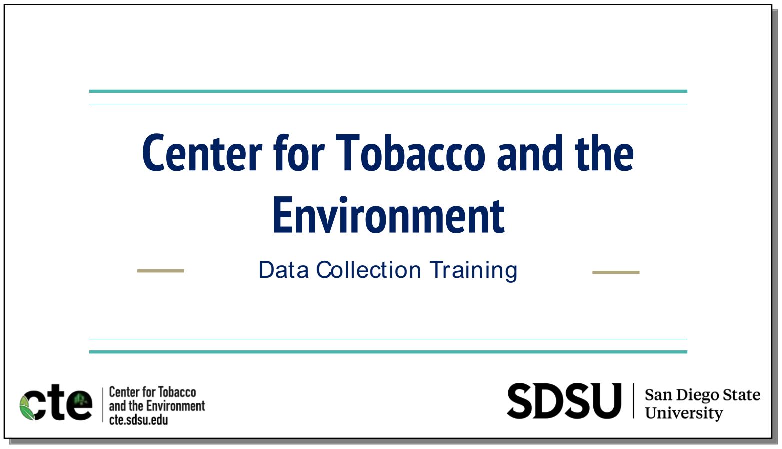

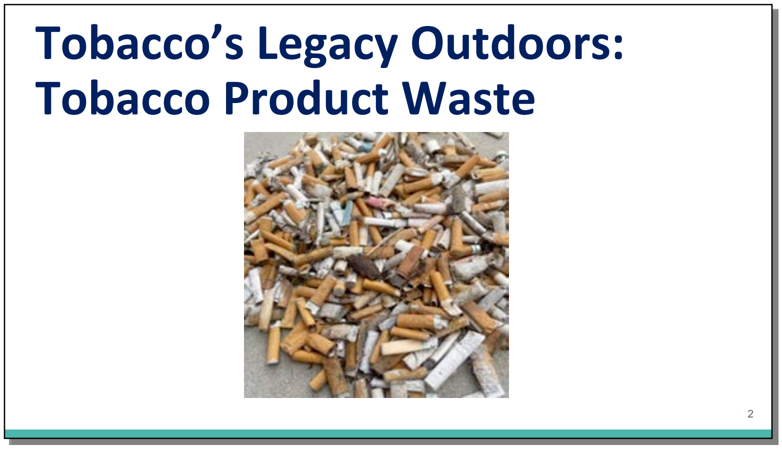

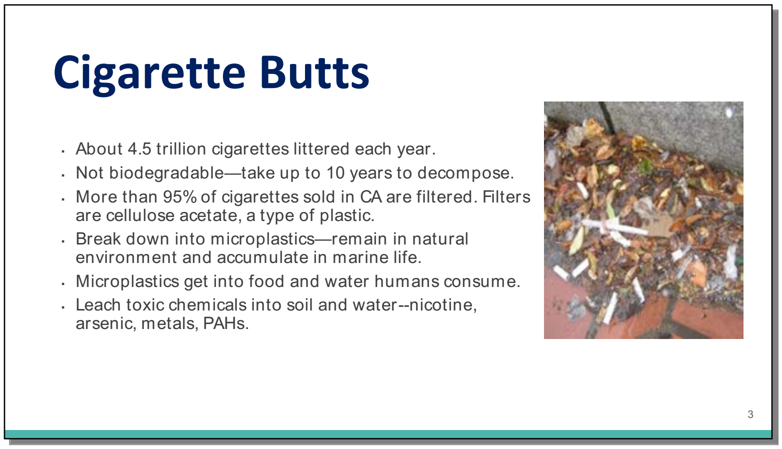

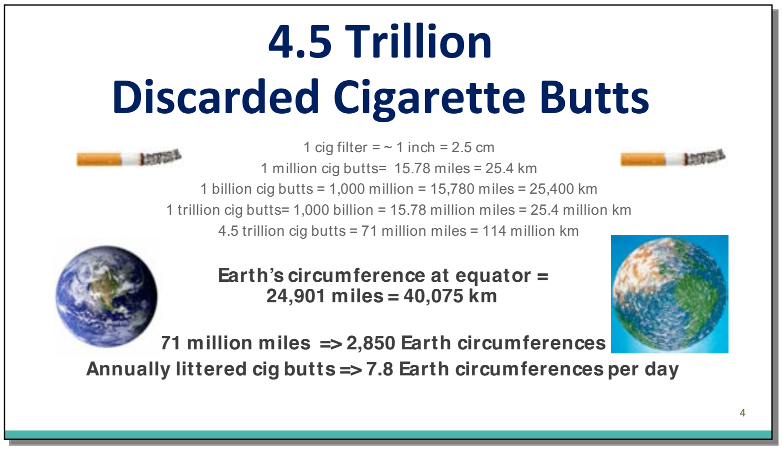
4.5 Trillion
Discarded Cigarette Butts
1 cig filter = ~ 1 inch = 2.5 cm
1 million cig butts= 15.78 miles = 25.4 km
1 billion cig butts = 1,000 million = 15,780 miles = 25,400 km
1 trillion cig butts= 1,000 billion = 15.78 million miles = 25.4 million km
4.5 trillion cig butts = 71 million miles = 114 million km
Earth's circumference at equator =
24,901 miles = 40,075 km
71 million miles => 2,850 Earth circumferences
Annually littered cig butts => 7.8 Earth circumferences per day
4

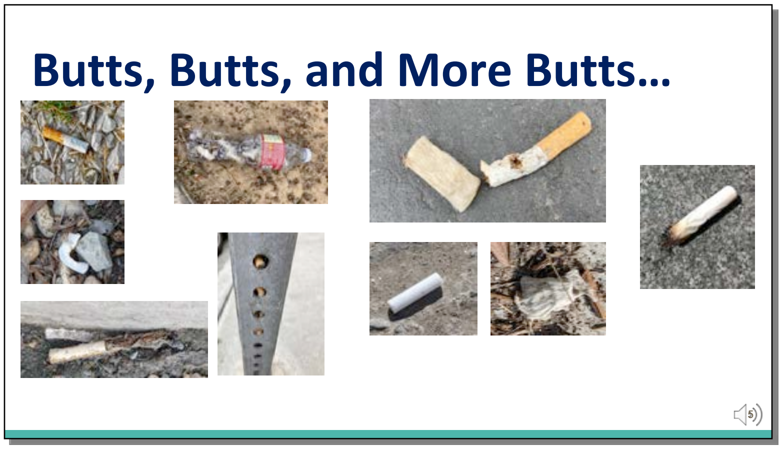
Butts, Butts, and More Butts…

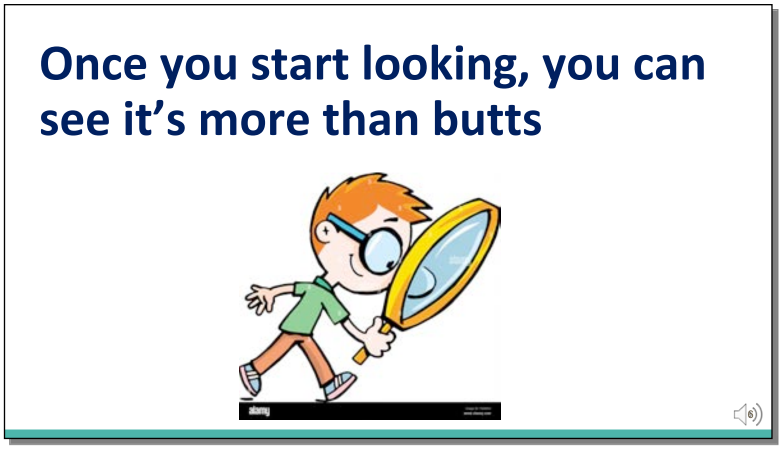
Once you start looking, you can
see it's more than butts

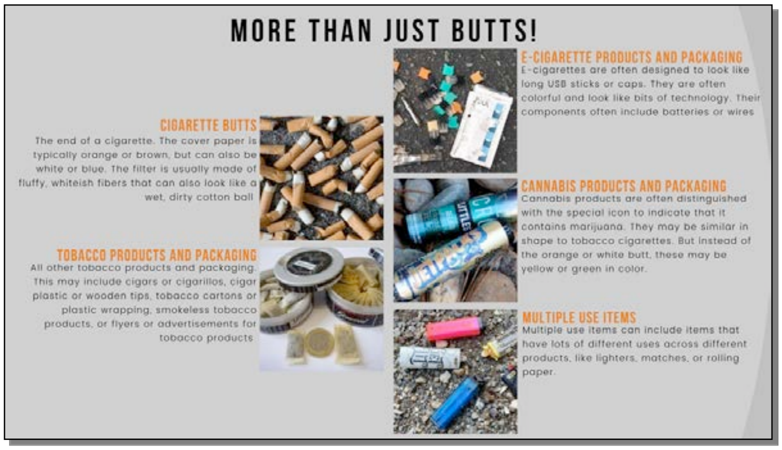
MORE THAN JUST BUTTS!
CIGARETTE BUTTS
The end of a cigarette. The cover paper is typically orange or brown, but can also be white or blue. The filter is usually made of fluffy, whiteish fibers that can also look like a wet, dirty cotton ball
TOBACCO PRODUCTS AND PACKAGING
All other tobacco products and packaging. This may include cigars or cigarillos, cigar plastic or wooden tips, tobacco cartons or plastic wrapping, smokeless tobacco products, or flyers or advertisements for tobacco products
E-CIGARETTE PRODUCTS AND PACKAGING
E-cigarettes are often designed to look like long USB sticks or caps. They are often colorful and look like bits of technology. Their components often include batteries or wires
CANNABIS PRODUCTS AND PACKAGING
Cannabis products are often distinguished with the special icon to indicate that it contains marijuana. They may be similar in shape to tobacco cigarettes. But instead of the orange or white butt, these may be yellow or green in color.
MULTIPLE USE ITEMS
Multiple use items can include items that have lots of different uses across different products, like lighters, matches, or rolling paper.

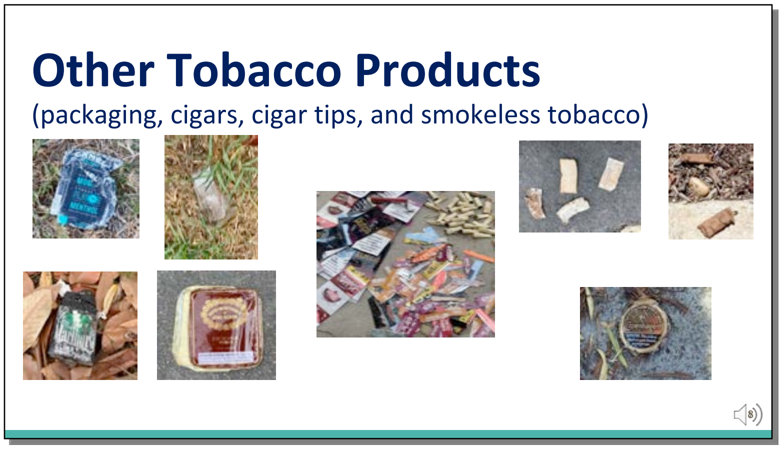
Other Tobacco Products
(packaging, cigars, cigar tips, and smokeless tobacco)

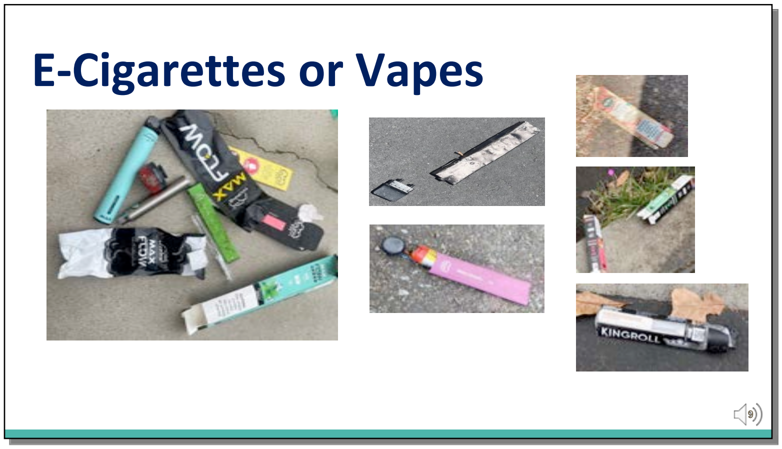
E-Cigarettes or Vapes
FLOW
MAX
KINGROLL

Cannabis

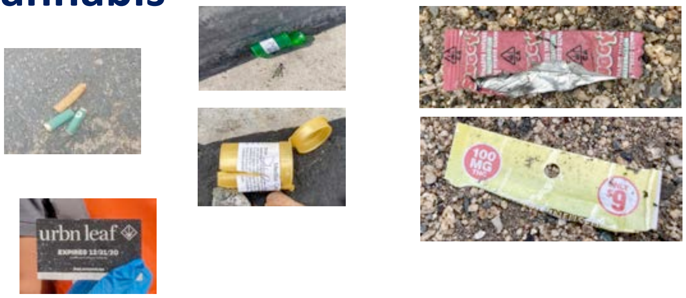

Multiple Use Products

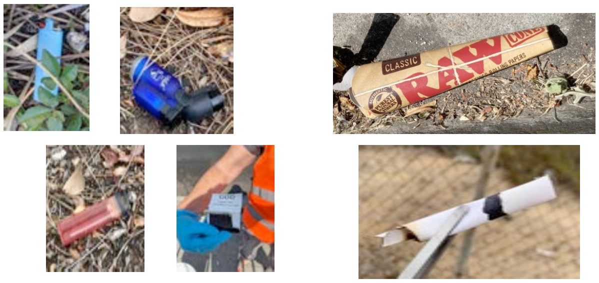

Hazards of Tobacco Waste

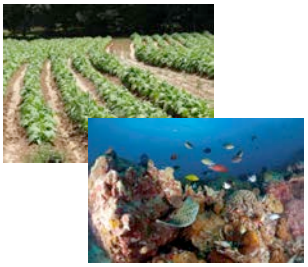
This Photo by Unknown Author is licensed under CC BY-SA

- Acetate filters in cigarettes
  - Filters degrade slowly and persist in the environment
  - Filters retain toxic tobacco smoke chemicals
  - Chemicals leach into soil and aquatic environments
- Electronic cigarettes (vapes)
  - Regulated biohazard
  - Regulated electronic hazard

California's EPA Restrictions

Cities must prevent all particles 5mm or larger (i.e., larger than ¼") from entering storm drains by 2030

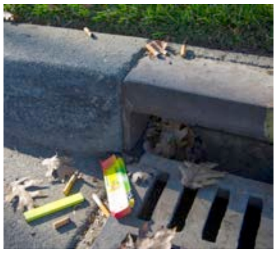

https://www.waterboards.ca.gov/water_issues/programs/stormwater/docs/trash_implementation/trash_amend.pdf

Water Quality Control Plan for Ocean Waters of California

13

Clean-up Costs Money!

- 30 large US cities
- Cost estimates ranged from $4-$90 million annually

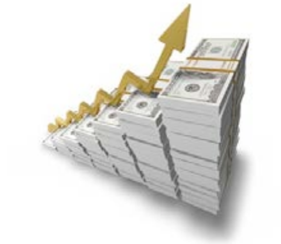

Online Simulation Model to Estimate the Total Costs of Tobacco Product Waste in Large U.S. Cities
J. E. Schneider, C. M. Scheibling, N. A. Peterson, P. Stigler Granados, L. Fulton and T. E. Novotny
International Journal of Environmental Research and Public Health 2020 Vol. 17 Issue 13 Pages 4705
Accession Number: doi:10.3390/ijerph17134705
https://www.mdpi.com/1660-4601/17/13/4705

14

One Minute Write

- Is it important to study tobacco product waste? Why or why not?
- If you were designing a study about tobacco product waste, what information would you want to collect and why?

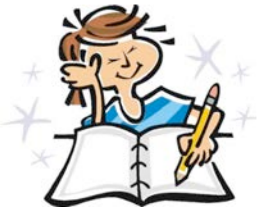

15

Study Design

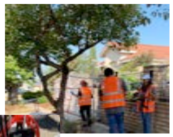

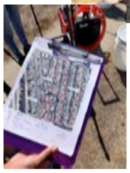

- **Random selection of 60 census blocks**
  - **Six** different categories of land use
  - **Two** socioeconomic levels
  - Total of 120 visits (visit each location twice)

16

Defining an Area

We collect data in public spaces only. We do not go onto private property.

"Regular areas" refer to public walkways on the perimeter or inside of a census block. The borders of regular areas are from the inside edge of the sidewalk to the gutter, including the grass or landscaped area between the sidewalk and gutter. Alleys are also defined as "regular areas".

- *In these areas, each piece of tobacco product waste is photographed where it is found.*

"Irregular areas" refer to public areas such as parking lots or picnic areas that are inside the perimeter.

- *In these areas, all tobacco product waste is collected and counted and the total of each type found in the space is recorded.*

17

Data Collection: Find and Identify

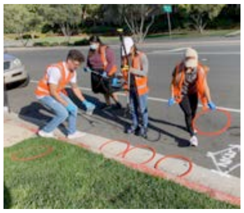

- Specific areas are assigned to each team member
- Rake debris that may hide tobacco product waste.
- Pick up all trash and examine it. Tobacco product waste may be disguised as regular trash, so everything needs to be inspected.
- To identify, refer to Tobacco Product Waste Identification Guide

18

Snowplow or Lawnmower?

When looking for tobacco product waste, we use two strategies to ensure we don't miss anything, the lawnmower and the snowplow!

Linear Pattern (aka Snowplow)

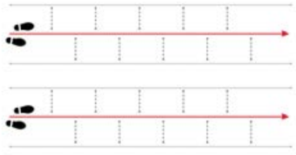

Lawnmower Pattern

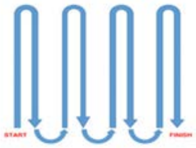

19

Data Collection Materials

- We will be using:
  - A grabber
  - Gloves
  - Collection bags/containers
- Make sure to bring:
  - Comfortable closed-toe walking shoes
  - Water
  - Sunscreen

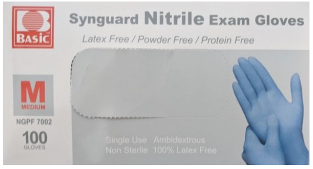

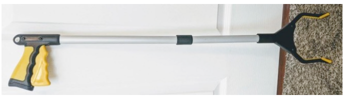

20

Data Collection: Photo and Code

If you have picked up the waste to identify it, place it back where you found it.
Place a hoop around the waste.
Position monopod next to hoop.
Using Ipad, take a photo (include entire hoop in photo)
Record type and #
If more than one type product waste take another photo, record type and #. Repeat until all waste recorded.
Pick up waste and place in appropriate carrier.
Remove hoop
STAY WITH YOUR HOOP UNTIL ALL STEPS ARE COMPLETED!

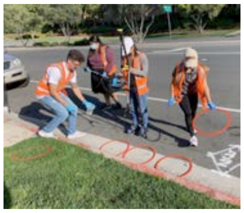

21

Data Collection: Pick up and Place

Pick up waste and place in appropriate collection container by type.
Remove hoop
STAY WITH YOUR HOOP UNTIL ALL STEPS ARE COMPLETED!

Data Collection: Quality Control

At the conclusion of data collection of each location, count all waste collected by type at that location.
Record count on paper data sheet.
Conduct a second pass on selected sites. Be sure to walk in the opposite direction as the first pass (you will see more waste this way!)

22

Data Tools: Maps and Arc GIS

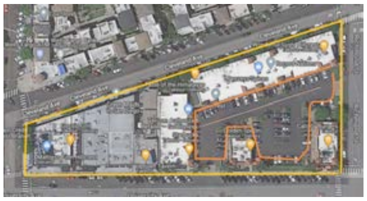

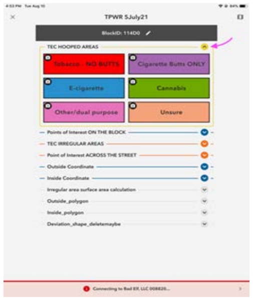

On Site Training

- Overview of data collection including:
  - Review of tobacco product waste identification
  - Safety protocols
- Demonstration:
  - "How to" find, count, categorize, and collect each type of waste
- A chance to practice:
  - Work alongside Research Assistants as part of a small team, each member is assigned specific tasks.

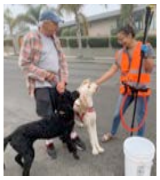

Data Collection Summary

1. Use the map and markings to make sure of the boundaries of the area you are assessing.
2. Use snowplow or lawnmower method so you do not miss any spots within the boundaries.
3. Walk slowly and look carefully for possible tobacco, e-cigarette, and marijuana waste.
4. If you see any kind of trash, examine it closely. Pick it up if necessary. Is it tobacco, e-cigarette, or marijuana waste?
5. If no, pick it up and throw it away
6. If yes, decide what kind of tobacco, e-cigarette, or marijuana waste it is.
7. Place a hoop around the waste.
8. Take a picture of the hooped area to geo-code the waste.
9. Record the # of items in the picture (for example, 3 cigarette butts).
10. If there is more than one type of waste in the hoop, repeat steps 7 and 8 until all waste is recorded. (If there are 2 types of waste in the hoop, you will need to take 2 pictures of the same hoop.)
11. Pick it up and put in collection container.
12. Count by location and type at conclusion of each site.
13. Make sure to pick up and discard any litter.

25

Cigarette Butts

How many cigarette butts can you find in this photo?

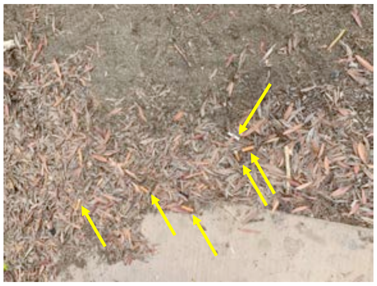

26

Safety First! Collecting Tobacco Product Waste

- It is very important that we aim to collect data as safely as possible
- Make sure to follow these steps when picking up tobacco, e-cigarette, and marijuana waste:
  1. Wear nitrile gloves on both hands. Replace your gloves if they get torn or dirty.
  2. Never pick up waste with your hands – always use a grabber to do so!
  3. Carefully place waste in the proper container – trash in the trash container, and tobacco product waste in the collection container
  4. When collecting, be sure to stay inside the boundary
  5. When finished, throw away both the trash container and the collection containers in an outdoor trash can. Do not bring any trash into your car or home!

27

Safety First! Take Care

To prevent injury (and in case of emergency):

- Bring a photo ID, just in case of emergency
- Wear long pants and comfortable walking shoes (no flip flops)
- Use sun protection (hat, sunglasses, sunscreen)
- Carry water, your phone, and ID in your pocket
- Wear the reflective vest and nitrile gloves (provided)

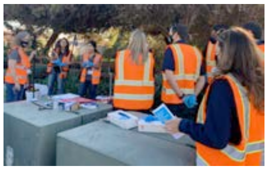

28

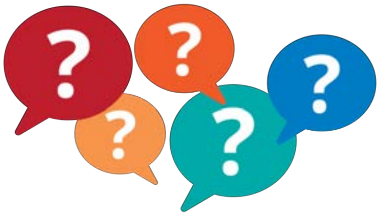

Questions? Contact Kris Tran at ktran14@sdsu.edu

29

Thank You!

30
