# Supplementary material for: Estimating the accumulation and re-accumulation of commercial tobacco, electronic cigarette, and cannabis waste based on a stratified random sample of census blocks
Source: PLoS One. 2025 Jan 6;20(1):e0313241. doi: 10.1371/journal.pone.0313241 (PMC11703088; doi:10.1371/journal.pone.0313241)
Supplement: S1 Fig — (PDF) [file pone.0313241.s003.pdf]

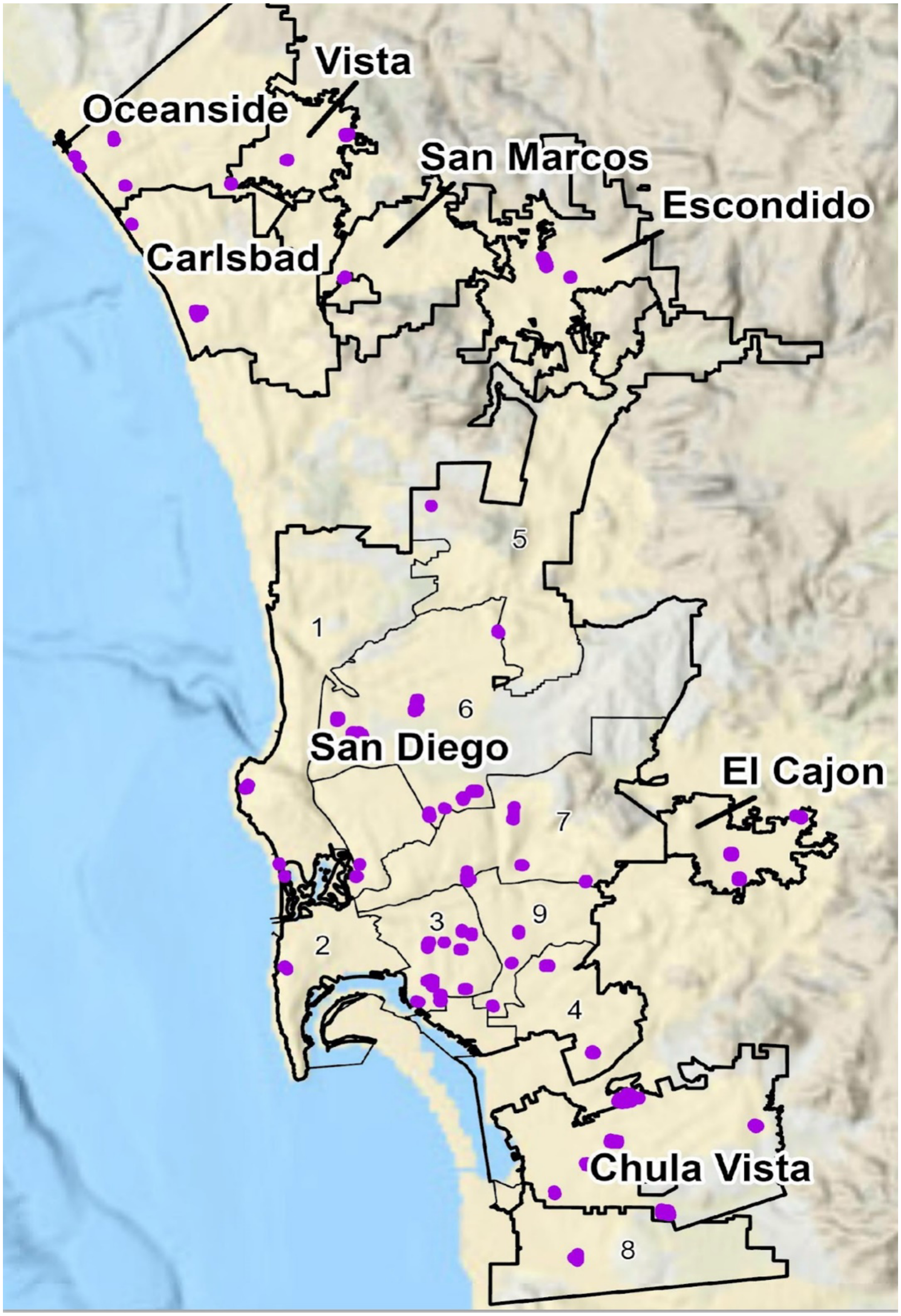

S3 Figure. The map shows the location of the 60 census blocks randomly selected from all census blocks representing the eight largest cities of San Diego County. The numbers 1 to 8 refer to the council districts of the City of San Diego, California.
