# Supplementary material for: Estimating the accumulation and re-accumulation of commercial tobacco, electronic cigarette, and cannabis waste based on a stratified random sample of census blocks
Source: PLoS One. 2025 Jan 6;20(1):e0313241. doi: 10.1371/journal.pone.0313241 (PMC11703088; doi:10.1371/journal.pone.0313241)
Supplement: S2 Fig — (PDF) [file pone.0313241.s004.pdf]

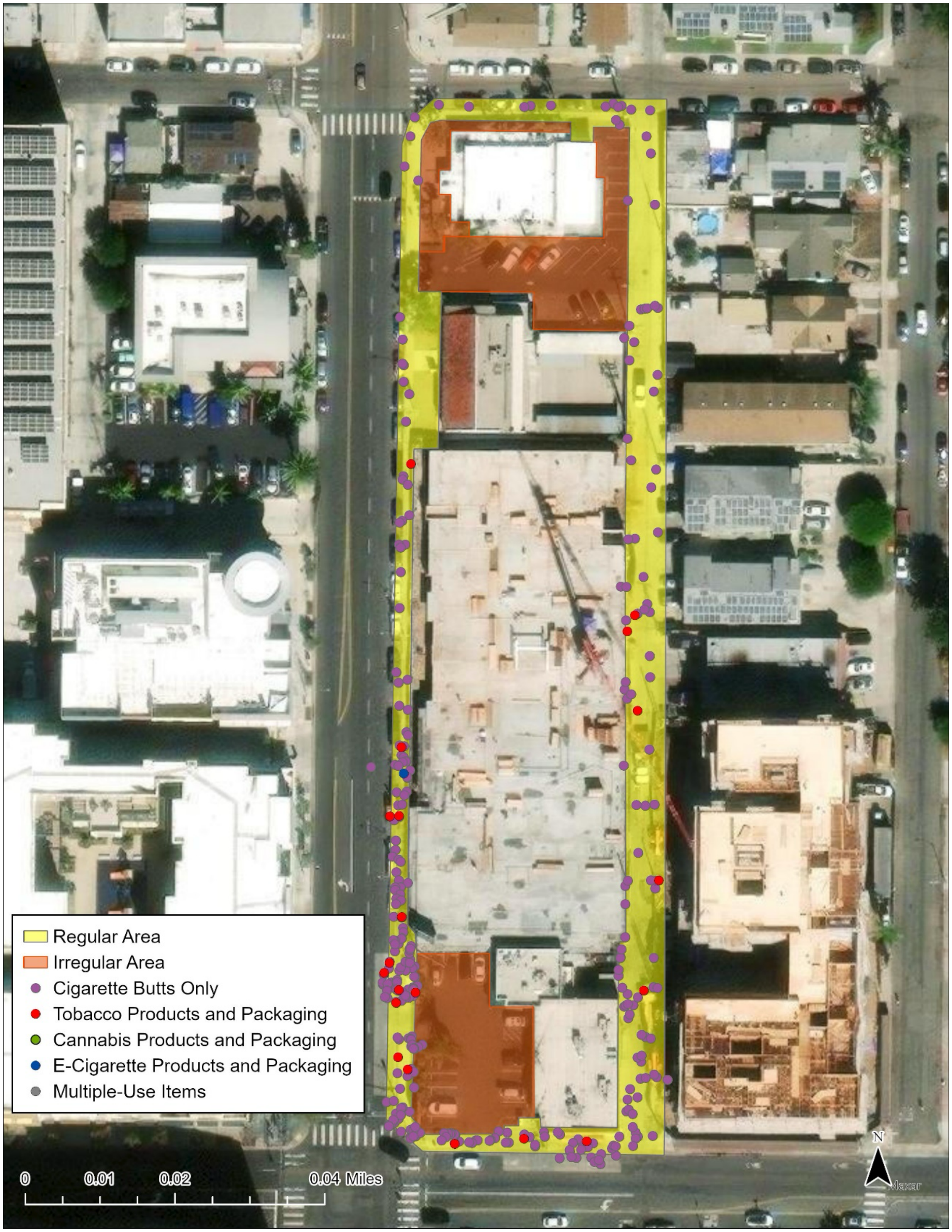

S4 Figure. This example block is located in the City of San Diego. The map shows tobacco product waste (small colored circles) located in regular (highlighted in yellow) and irregular (highlighted in orange) areas that were surveyed for the present study by research staff.
